# Modified Alliance-Focused Training with Doubling as an integrative approach to improve therapists’ competencies in dealing with alliance ruptures and prevent negative outcomes in psychotherapy for depression: study protocol of a randomised controlled multicentre trial

**DOI:** 10.1136/bmjopen-2024-098343

**Published:** 2025-07-16

**Authors:** Antje Gumz, Denise Kästner, Laurence Reuter, Carmen Martinez Moura, Klarissa Ehlers, Anne Daubmann, Catherine F Eubanks, John Christopher Muran, Timothy Anderson, Ramona Stöckl, Georg Schwanitz, Lena Stegemann, Lana Rohr, Ulrike Willutzki, Frank Jacobi, Antonia Zapf

**Affiliations:** 1 Department of Psychosomatics and Psychotherapy, Psychologische Hochschule Berlin, Berlin, Germany; 2 Institute of Medical Biometry and Epidemiology, University Medical Center Hamburg-Eppendorf, Hamburg, Germany; 3 Derner School of Psychology, Adelphi University, Garden City, New York, USA; 4 Psychotherapy Research Program, Icahn School of Medicine at Mount Sinai, New York, New York, USA; 5 Department of Psychology, Ohio University, Athens, Ohio, USA; 6 Clinical Trial Office, Charité Universitätsmedizin Berlin, Berlin, Germany; 7 Department of Psychology and Psychotherapy, University Witten/Herdecke, Witten, Germany; 8 Department of Clinical Psychology and Psychotherapy, Psychologische Hochschule Berlin, Berlin, Germany

**Keywords:** Depression & mood disorders, Clinical Trial, EDUCATION & TRAINING (see Medical Education & Training), Psychosocial Intervention

## Abstract

**Introduction:**

Alliance ruptures constitute a high risk of premature treatment termination and poor psychotherapy outcome. The Alliance-Focused Training (AFT) is a promising transtheoretical approach to enhance therapists’ skills in dealing with alliance ruptures.

**Methods and analysis:**

To evaluate the effectiveness of Modified AFT with doubling (MAFT-D), a randomised, patient and evaluator-blinded, multicentre trial was designed comparing MAFT-D (delivered to trainee therapists and supervisors) and psychotherapy training/treatment as usual (TAU) for therapists (n=120) and their patients with depressive disorders (n=240). A total of 17 cooperating centres, each offering either cognitive-behavioural or psychodynamic psychotherapy training, will contribute to recruitment. Stratification by centre (both for therapists and patients) and hence therapeutic approach (cognitive-behavioural vs psychodynamic psychotherapies), and by comorbid personality disorder (yes vs no, for patients) will be carried out. The two hierarchically ordered primary hypotheses are: In MAFT-D compared with TAU, a stronger reduction of depressive symptoms and a lower rate of patient dropout is expected from baseline to 20 weeks after baseline. Follow-up assessments are planned at 35 weeks, 20 months and 36 months postbaseline to evaluate the persistence of effects. Secondary patient-related and therapist-related outcomes as well as predictors, moderators and mediators of change will be investigated. Mixed models with repeated measures will be used for the primary analyses.

**Ethics and dissemination:**

Ethical approvals were obtained by the institutional ethics review board of the main study centre as well as by review boards in each federal state where one or more cooperating centres are located (secondary votes). Following the Consolidated Standards of Reporting Trials statement for non-pharmacological trials, results will be reported in peer-reviewed scientific journals and disseminated to patient organisations and media.

**Trial registration number:**

DRKS00014842; https://drks.de/search/de/trial/DRKS00014842.

**STRENGTHS AND LIMITATIONS OF THIS STUDY**

- This large randomised controlled multicentre trial evaluates the effects of Modified Alliance-Focused Training with doubling (MAFT-D) (delivered to trainee therapists and supervisors) from both therapist and patient perspectives, ensuring a comprehensive assessment of its impact.
- Involving multiple psychotherapy training institutes not only ensures generalisability but may also facilitate transfer into clinical training and practice.
- By focusing on depression while stratifying for comorbid personality disorders, the study also stands to clarify potential differential effects of MAFT-D depending on patients’ psychopathological characteristics.
- The supervision frequency (one session per four therapy sessions), aligned with local real-world practices, and the primary measurement point (20 weeks), chosen for comparability with other trials, together pose a risk of insufficient dose, with effects potentially becoming apparent only at later follow-ups.

## Background

 Depressive disorders are among the most common mental disorders^1^ and the leading cause for disability worldwide.^2^ The course of the disorder is often recurring or chronic, and the consequences for the individual and the society can be severe.^1^ The guideline-recommended treatments for depression are pharmacotherapy, psychotherapy or a combination of both (depending on severity and chronicity). Cognitive-behavioural and psychodynamic psychotherapies (CBT and PDT) are evidence-based and comparably effective.^13^,^5^ However, unsatisfactory outcomes in depression treatments are frequent (up to 41% of patients do not reliably improve, up to 1/3 drop out).^6^,^8^ Thus, factors closely linked to outcome and dropout need to be addressed. Here, the evidence clearly points to the importance of common factors (relevant across therapeutic approaches), especially the therapeutic alliance.^9^,^12^

Alliance ruptures (ie, periods of tension or breakdown of the collaborative relationship between patient and therapist) inevitably occur in the course of any psychotherapy.^13^ Across various therapeutic approaches, there is an ever-growing wealth of evidence establishing a link between alliance ruptures and treatment outcome, whereas the therapists’ ability to repair ruptures has been demonstrated to improve outcome and to prevent dropout.^14^,^27^ However, therapists often fail to notice ruptures or lack the skills to deal with them constructively.^1328^,^32^

Consequently, it is of crucial importance to improve the competence of therapists in dealing with alliance ruptures. This is in line with current recommendations according to which psychotherapy trainings should give more emphasis to common therapy principles like improving the alliance.^33^,^35^ Despite the common occurrence of alliance ruptures and the serious implications resulting from failures to resolve them, the curricula of psychotherapy training do not systematically address the topic as of yet.

Various therapist trainings focus on therapeutic relational skills and/or the process of alliance rupture and repair. Among them, the most prominent is Alliance-Focused Training (AFT^36 37^), which follows a transtheoretical approach and is grounded in an evidence-based model developed by Safran, Muran and Eubanks for successfully resolving alliance ruptures.^13 36^ Its main objective is to strengthen three skills: (1) to sensitively notice alliance ruptures, (2) to tolerate difficult affects and (3) to meta-communicate about them in a helpful way. Video-recorded sessions and role plays are fundamental elements of AFT.^37^,^39^ Building on our pilot study (funded by the Heigl Foundation), we made several modifications to the training, the most significant of which was the incorporation of the psychodrama technique of doubling after role plays to enhance the focus on affective communication and to foster a secure, non-judgemental atmosphere in both training and supervision^40 41^ (details see section ‘Experimental intervention’).

Previous research emphasises the potential of therapist training programmes targeting the therapeutic alliance in general, and of AFT specifically.^20 42 43^ A meta-analysis of empirically supported relationship factors^44^ describes the systematic resolution of alliance ruptures as one (out of three) of the most promising relationship elements that warrant further research. Two recent studies demonstrated an effect of AFT on therapists’ skills.^42 43^ Evidence on patient outcomes appears promising regarding the reduction of dropout rates but remains unclear in terms of symptom improvement.^20 22^ Overall, the evidence on the effectiveness of training focusing on the alliance seems still rather limited. The studies included in the existing reviews and meta-analyses^20^,^22^ were highly heterogeneous with respect to the investigated patient populations, treatment lengths, scope and content of the training intervention. Moreover, the sample size of most studies was small. Promising results were found with respect to patients with depressive disorders (evidence base: study 1: n=31 intervention group (IG) 1; n=34 IG 2; n=38 control group (CG); 8 sessions problem solving therapy^45^; study 2: n=11 IG, n=11 CG, 16 sessions cognitive therapy^46^).

Despite the central role of relationship problems among patients with depression^47^,^49^ and the high proportion of negative or insufficient therapeutic outcomes, there is no large-scale trial investigating the benefits of training with a focus on the therapeutic alliance in depression. More studies are needed to investigate the effectiveness of AFT and to link the increase in therapist competence to outcome.^50^

This is a central aim of the present study. While it is a common assumption that improvements in therapist skills lead to better therapy outcomes, few studies evaluating therapist trainings have actually assessed patient outcomes. Instead, most prior research has measured therapist skill improvements as the primary outcome, often relying on self-assessments or evaluations by external observers.^43^ In contrast, this study focuses on patient outcomes as the primary endpoint—specifically, the patients treated by therapists who received the training. While this approach aligns more directly with the ultimate goal of therapist training, it has been under-represented in previous research.

Against this background, our study intends to investigate the benefits of our modified form of AFT (MAFT-D) versus psychotherapy treatment as usual (TAU) for patients with depressive disorders. The decision to investigate patients with depressive disorders is based on the facts that (a) to date there is no large-scale study investigating the effects of AFT in depression, (b) depressive disorders are highly prevalent and debilitating, (c) interpersonal problems are key factors in models on the development and maintenance of depression^48 49^ and (d) negative outcomes in depression treatments are particularly frequent.^6^,^8^ Notably, AFT has so far been more widely studied in the context of personality disorders,^43 51^ with limited evidence available for patients with depression. This study focuses on patients with depression but includes those with comorbid personality disorders, stratifying for this variable. Thus, the study also stands to clarify potential differential effects of MAFT-D depending on patients’ psychopathological characteristics, contributing to a more nuanced understanding of alliance-focused approaches across various disorders.

The project investigates CBT and PDT therapists, because of the transtheoretical nature of AFT, the great significance of common factors, and the urgent need for evidence-supported methods in psychotherapy training. Training and therapy under routine conditions represent the reference for efficacy and safety for the experimental intervention because CBT and PDT are established evidence-based treatments for depression, and psychotherapy training at the state-approved institutes can also be considered as the current gold standard.

Additionally, the project intends to investigate mediators of MAFT-D effectiveness as well as further evidence-based mediators of change, as well as predictors and moderators of therapy success. The consideration and differentiation of patient and therapist effects, as well as various common factors measured from different perspectives (patients, therapist and observer), may contribute to a better understanding of change mechanisms in psychotherapy overall, which constitutes a critical step towards more effective treatments.

## Methods

### Study design

**Figure 1:**
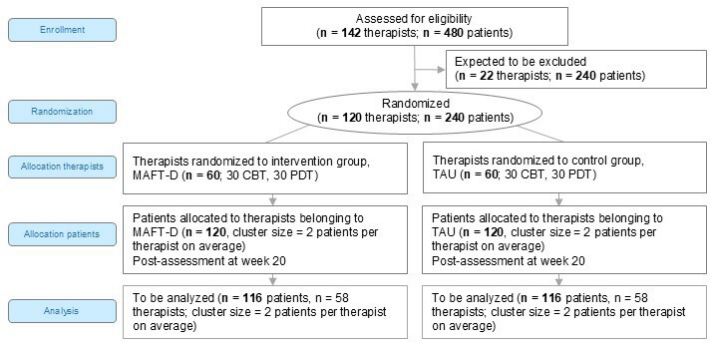
Trial design. In general, recruitment at a given centre will be concluded once the target number of patients per centre has been reached. Minor deviations in the number of study patients treated by each therapist (ie, one or three instead of two) are allowed, for example, due to the therapist’s availability and number of open treatment slots. To ensure that the calculated sample size is achieved, up to five additional units (ie, 40 therapists, 80 patients, 10 supervisors) may be recruited. CBT, cognitive–behavioural therapy; MAFT-D, Modified Alliance-Focused Training with Doubling; PDT, psychodynamic therapy; TAU, psychotherapy treatment as usual.

Patients, their trainee therapists and therapists’ supervisors will take part in the randomised controlled multicentre trial. The trial intends to investigate the benefits of MAFT-D versus TAU for patients with depressive disorders, stratified by the cooperating centre (psychotherapy training institute), subsequently by therapeutic approach (CBT vs PDT) and comorbid personality disorder (SCID-5-PD, yes vs no), as well as their trainee therapists, stratified by cooperating centre (psychotherapy training institute) and therapeutic approach (CBT vs PDT). Therapists and supervisors belonging to the IG will receive MAFT-D. Therapists and supervisors belonging to the CG will receive/conduct psychotherapy TAU. Figure 1 illustrates the study design. The trial is registered at the German Clinical Trial Register (DRKS00014842).

### Hypotheses and research questions

The two hierarchically ordered primary hypotheses are: In MAFT-D compared with TAU, a stronger reduction of patient-rated depressive symptoms (first primary endpoint) and a lower rate of patient dropout (second primary endpoint) is expected from baseline to 20 weeks after baseline. The study’s hierarchical testing framework first assesses symptom reduction, followed by dropout rates. While this sequence reasonably suggests that dropout rate effects might be less relevant without significant symptom reduction, we would like to point out that one could also argue that both hypotheses hold independent value and could merit separate evaluation, given their relevance to treatment quality. While the time point (after 20 weeks) was chosen carefully to ensure comparability with other studies, it carries the risk that the intervention dose might be insufficient at this stage, with effects potentially becoming evident only at later follow-ups.

Secondary patient-related hypotheses are: In MAFT-D compared with TAU, stronger reduction of patient-rated depressive symptoms after 35 weeks, and 20 and 36 months, lower rate of dropout at week 35 and month 20 and 36, stronger reduction of observer-rated depressive symptoms, and patient-rated anxiety, somatic complaints, personality structure deficits, interpersonal problems, and quality of life after 20 and 35 weeks, and 20 and 36 months.

Secondary therapist-related hypotheses are: In MAFT-D compared with TAU, stronger increase in observer-rated interpersonal skills after 35 weeks, and therapist-rated therapeutic skills, trait-like relational manner in therapy, emotional suppression, alexithymic traits (difficulties identifying and describing feelings) and satisfaction with supervision after 20 and 35 weeks, and 20 and 36 months.

The hypotheses regarding mediators of MAFT-D effectiveness are: MAFT-D generates more favourable outcomes via an improved therapeutic alliance and increased therapist skills to deal with alliance ruptures. Corresponding mediator variables are: (1) therapeutic alliance,^52^ (2) ratio of unresolved/resolved alliance ruptures,^22^ (3) convergence of patients’ and therapists’ alliance ratings over time,^14 53 54^ (4) interventions referring to the therapeutic relationship in the here and now,^13^ (5) nondirective supportive therapist’s techniques,^55 56^ (6) therapists’ competence in session (empathy) and^57^ (7) adherence to MAFT-D.^13 22^

Subsidiary research questions concern predictors, moderators and mediators of (a) therapeutic macro and micro outcomes (eg, dropout, alliance), (b) the effectiveness of MAFT-D (eg, presence of a personality disorder or therapeutic orientation as moderators of MAFT-D effectiveness), (c) observer-rated interpersonal therapeutic skills and skills development and (d) the successful resolution of alliance ruptures.

### Methods against bias

Randomisation and stratification: Therapists are randomly assigned by a 1:1 ratio to receive MAFT-D versus TAU stratified by study centre and approach (CBT/PDT). Patients are randomly assigned to MAFT-D versus TAU stratified by study centre, subsequently by approach (CBT/PDT), and comorbid personality disorder (SCID-5-PD, yes vs no). Since it emerged that each cooperating centre offers training in only one therapeutic approach, stratification by therapeutic approach proved to be practically redundant. Block randomisation with variable block length is used. Patients are assigned to therapists appropriate to the random group. Randomisations will be performed centrally by Professor Zapf’s group, University Medical Center Hamburg Eppendorf. Allocation is done via the electronic case report forms (eCRF) (implemented in secuTrial).

Blinding: Therapists will be aware of allocation, patients will be blinded. Interviewers, raters and persons conducting the statistical analyses will be blinded. There will be strictly separate role assignments in the study team (raters/interviewers vs unblinded study team members). Raters/interviewers will work physically separated from unblinded staff.

Allegiance bias: Principal investigator (PI), research group members and national or international collaborators are PDT, CBT or systemic therapists, recruiting centres are from both therapeutic approaches, statisticians have no affiliation to a therapeutic approach.

Contamination bias: All MAFT-D supervisors and therapists will be asked to treat the contents of the intervention highly confidential and to give a standardised response to respective questions. MAFT-D therapists will receive a two-staged informed consent (second stage: description of MAFT-D, after randomisation). Part of the intervention (workshop) will be offered to TAU-therapists at the end of follow-up. Supervisors in the IG will document the total number of cases discussed during each supervision session and the number in which MAFT-D was used. The supervisors are also asked retrospectively about the perceived usefulness of individual MAFT-D elements. Additionally, the number of hours therapists spent engaging with crucial elements of the intervention—such as observing role plays, participating in role plays, watching video-recorded therapy sessions of other therapists and reviewing their own video-recorded sessions—is included in the ‘basic characteristics, experience and practice’ section of the assessment battery. Therapists complete this section at all fixed measurement points (BA, Post, FUP 1, 2, 3). Differences between therapists in the IG and the CG regarding these items will be analysed. To estimate the extent of contamination bias, an adherence measure score (Beth Israel Fidelity Scale, BIFS^58^) will be applied to the video-recorded therapy sessions and analysed as a dependent variable using a linear mixed model.

Other biases: Data monitoring will be conducted by the Clinical Study Office Charité Berlin (CTO). In order to minimise potential bias due to video recordings, all therapy sessions will be recorded. The number of theoretical hours and supervision sessions completed will be compared for MAFT-D and TAU therapists. The use of additional treatments of the patients is monitored and analysed (Client Sociodemographic and Service Receipt Inventory, CSSRI^59^). Additional treatments that could influence outcomes, such as newly prescribed antidepressants or psychopharmacological medications, the repeated use of outpatient psychotherapeutic or psychological services (eg, couples counselling exceeding four sessions), or psychiatric or psychosomatic day-patient or inpatient stays, are considered protocol violations. Therapists’ training preferences are analysed as efficiency modifiers. To assure generalisability, we use a multicentre design. To assess selection bias, we will document ineligible persons among patients (basic demographic information, reason for non-participation). Cases of withdrawal who allow the use of their data will be compared with participants who do not drop out regarding baseline differences. We will document reasons for study discontinuation. Incentives are used to reduce missing values in other outcomes (eg, €150 dropout postassessment).

### Inclusion and exclusion criteria for patients

Inclusion criteria: (a) Agreement to start CBT or PDT outpatient therapy, weekly 50 min sessions; (b) Age ≥18 years; (c) Present DSM-5 diagnosis of depressive and related disorders (major depressive disorder—single or recurrent episode; persistent depressive disorder, other specified depressive disorder). Operationalisation: Structured Clinical Interview for DSM-5 Disorders, Clinician Version, SCID-5-CV,^60 61^ conducted by trained raters. The SCID-5-CV shows excellent to satisfactory inter-rater reliability, via phone and face-to-face^62 63^; (d) Reaching or exceeding the cut-off value indicating moderate depression severity. Operationalisation: Beck Depression Inventory II, BDI-II,^64 65^ score≥20^1 66^ and (e) Signed informed consent form.

Rationale for the criteria: Depression is the most common mental disorder in Germany, associated with considerable costs.^67^ Psychotherapy is recommended for patients with moderate or severe depression.^1^ Interpersonal problems are key factors in models on the development and maintenance of depression.^47^,^4968^

Exclusion criteria: (a) Current or past diagnosis of a psychotic or bipolar disorder; (b) Substance dependency disorder (current or during the last 12 months); SCID-5-CV at time point of screening; (c) Acute suicidal plans at time point of screening or suicide attempts within the last 6 months; modified (last 6 months) Colombia Suicide Severity Rating Scale,^69^ which is a reliable and valid instrument for identification of suicide risk^69 70^; (d) Psychopharmacotherapy other than antidepressants; change of antidepressant regimen during the previous month or during study participation; modified CSSRI,^59^ which is one of the most widely used instruments for a standardised assessment of healthcare utilisation (including medications; medication classification will be based on ATC-codes)^59^; (e) Planned concurrent psychological treatments (Self-help groups can be continued.); CSSRI and (f) Insufficient command of the German language, below level B2.

Rationale for the criteria: Severe comorbidities are excluded for reasons of patients’ safety. Concomitant treatments are limited to minimise performance bias. The number of exclusion criteria is kept low to ensure representativeness for routine care.

### Inclusion criteria for therapists and supervisors

Therapists: (a) graduated psychologist or licensed physician; (b) in advanced CBT or PDT training (with a licence to practice outpatient therapy under supervision, which is usually obtained after 2 years of training); (c) capacity to start treatment with two new patients and (d) capacity to visit one of the workshops offered for study therapists.

Supervisors: (a) approved by state and cooperating institute, (b) no scheduling conflicts (eg, workshop dates, regular dates for group supervision) and (c) agreement to decline requests for supervision from study therapists of the other condition during the study period.

There are no exclusion criteria for therapists and supervisors.

### Cooperating study centres

The main study centre is at the Department of Psychosomatics and Psychotherapy, Psychologische Hochschule Berlin where study coordination and data collection take place. The cooperating centres across Germany are state-approved, well-established psychotherapy training institutes offering courses in individual outpatient psychotherapy (CBT or PDT). By involving numerous institutes, the study ensures generalisability within a naturalistic setting. Furthermore, the integration of MAFT-D into various existing training and continued education programmes could be facilitated in case of positive evaluation results.

The cooperating centres support the recruitment of participants and are responsible for the organisation and carrying out of the study-independent regular therapy training and patient treatments. The following centres agreed to cooperate (alphabetically ordered, therapy approach and person responsible in brackets):

1. Akademie für Psychotherapie Erfurt (PDT; Professor M. Geyer).
2. Berliner Akademie für Psychotherapie/Masterstudiengang Verhaltenstherapie, Psychologische Hochschule Berlin (CBT; Professor F. Jacobi).
3. Centrum für Integrative Psychologie Bamberg (CBT; Dr. J. Siegl).
4. DGVT-Ausbildungszentrum Dortmund/Universität Witten/Herdecke (CBT; Professor U. Willutzki, A. Radix).
5. Dresdner Institut für Psychodynamische Psychotherapie (PDT; Dr C. Schilling, Dr S. Seifert).
6. IFT—Psychotherapeutische Ambulanz, München (CBT; S. Gmeinwieser).
7. Institut für Psychotherapie und Angewandte Psychoanalyse, Jena (PDT; Dr U. Wutzler, M. Lüneberg).
8. Institut für Psychotherapie, Universitätsklinikum Hamburg-Eppendorf (PDT; Professor K. M. Reininger).
9. Köln-Bonner Akademie für Verhaltenstherapie, Bonn (CBT; Dr L. Miebach).
10. Leipziger Ausbildungsinstitut für Psychologische Psychotherapie und Universität Leipzig (CBT; Professor C. Exner, Dr S. Koranyi).
11. Masterstudiengang Psychodynamische Psychotherapie, Psychologische Hochschule Berlin (PDT; Professor A. Gumz).
12. ppt-Institut für Psychologische Psychotherapie und Beratung Berlin (PDT; Dr L. Hauten, E. Stahl, A. Rehbein).
13. Sächsisches Institut für Psychoanalyse und Psychotherapie, Leipzig (PDT; Professor K. v. Klitzing, Dr O. Krauß, Dr Anja Schmitt, Maria Johne).
14. Saarländisches Institut für Tiefenpsychologisch fundierte Psychotherapie, Saarbrücken (PDT; Dr E. Hahn, W. Bauer-Neustädter).
15. Weiterbildungsstudiengang Psychodynamische Psychotherapie, Klinik und Poliklinik für Psychosomatische Medizin und Psychotherapie, Universitätsmedizin der JGU Mainz (PDT; Professor M. Beutel).
16. Zentrum für Ausbildung in Psychologischer Psychotherapie, Friedrich-Alexander-Universität Erlangen-Nürnberg (CBT; Professor M. Berking, Dr K. Zierhut).
17. Zentrums für Psychologische Psychotherapie der Universität Greifswald (CBT; Professor E.-L. Brakemeier, M. Tewes).

Other centres will be involved in the event of recruitment difficulties.

### Recruitment

Recruitment will take place at the cooperating study centres (see above). Additional centres will be involved in the event of recruitment difficulties. Recruitment started in July 2024 and is ongoing.

The standard group size for group supervision is four participants, which applies to the study and also to routine therapy training. Consequently, units of eight therapists must usually be recruited per institute. Some institutes allow smaller group sizes. However, a group consisting of only two participants on a long-term basis (more than 4 months) is considered a protocol violation. Combining study therapists from different training institutes at one location in a supervision group is permitted. The groups can also be filled with non-study participants. Changing the format from group to individual supervision is generally not permitted within the context of the study. Recruitment methods for therapists include project promotion through email, advertisement in lectures/seminars. Patients are recruited from the regular outpatient pool. Recruitment period is 21 months for patients and 16 months for therapists. A key challenge in this study is the time-sensitive recruitment of integrated units comprising patients, therapists and supervisors. Due to the interdependence of these participants, unexpected, short-notice dropouts may jeopardise not only the involvement of the individual concerned but also the participation of the entire unit. To ensure the required sample size is reached, we plan to recruit up to 5 additional units (ie, 40 therapists, 80 patients and 10 supervisors) as a buffer for potential dropouts. Patients, therapists and supervisors will receive incentives for study-related expenses (assessments, video recordings and travel).

### Allocating patients to study therapists

The assignment of a patient to a study therapist is done by the main study coordinator. Besides the randomisation result, time availability (for the weekly sessions), other preferences (eg, gender of the therapist, treatment location) of both the patient and the therapist, and the therapist’s current study case load will be taken into account. Due to feasibility reasons, we refrained from considering further factors (eg, balancing patients with or without personality disorders at the therapist level). In rare cases, a randomised patient may not be allocated to a study therapist. This occurs when all therapy slots in the respective study group are filled, or when no available therapist can agree with the patient on a suitable time for their weekly appointments. After allocation, the main study coordinator informs the local study coordinator, and the respective therapist will receive the patient files and all relevant information and documents for the process assessments. The therapist is instructed that they should offer an appointment as soon as possible and start treatment.

### Patient treatments

Basic conditions of treatments will be equal between MAFT-D and TAU. Patients will receive weekly 50 min sessions (regular frequency and length). Postassessment will take place at week 20, first follow-up at week 35, second and third follow-up after 20 and 36 months. Treatments can be short-term or long-term depending on the patient’s and therapist’s agreement and the sessions granted by payers. According to usual care, all treatments must begin with probatory sessions (2–4 sessions before official start of therapy). Following this, the number of sessions requested by therapists and patients, and covered by payers in Germany, varies, ranging from at least 12 to up to 80 therapy sessions for CBT and up to 100 for PDT. All sessions will be video recorded. Therapists will treat two study patients on average. The costs of treatments are fully covered by statutory health insurances in Germany.

### Psychotherapy training

TAU-D and MAFT-D therapists will continue to participate in their regular training courses. Group supervisions of treatments will be carried out by experienced supervisors either with MAFT-D specific focus or according to the usual procedure. Supervision frequency and format (one session for every four therapy sessions, in person, additional individual supervision is permitted in accordance with the regulations of the specific institute in the event of crises or for questions regarding the report for the health insurance company) represents the common standard and legal requirement for trainee therapists in our country. This frequency might deviate from other clinical trials or the clinical practice in other regions. The supervision frequency may influence outcomes but also reflects local real-world practice conditions, adding ecological validity to the study. The supervision groups are fixed, and in order to participate, the therapist must have already started at least one study therapy. Group supervisions will occur monthly or biweekly, with patients being discussed for varying amounts of time (10–50 min per case). Supervisors will receive regular supervision fees. The hours of the workshop for the MAFT-D group will replace other non-mandatory courses of the regular training.

### Experimental intervention

Therapists randomised to MAFT-D will receive a 2-day workshop (approx. 15 teaching units, 11.5 hours) followed by group supervision carried out by supervisors who will also be trained in MAFT-D. The initial workshops will take place in person (for therapists and supervisors separately). Central elements are a theoretical introduction (150–180 min) and role plays based on individual case examples (approx. 45 min per participant). The number of lecturers depends on the overall group size (one lecturer and max. eight therapists per role play subgroup). In the case of unexpected absences (eg, illness) and for therapists who are unable to attend at the originally planned workshop dates, catch-up workshops will be offered. Catch-up workshops consist of smaller groups and are shorter (approx. 10 teaching units, 7.5 hours) but are more intense due to more frequent participation in role plays.

AFT is a transtheoretical training concept initially developed by Eubanks-Carter *et al*^36^ focusing on alliance ruptures and aiming at enhancing therapists’ interpersonal skills to (a) sensitively recognise alliance ruptures, (b) tolerate negative affects and (c) meta-communicate using video-recorded sessions, role plays and mindfulness exercises.^13 36 40 41^ We have translated the AFT into German and tested and modified it in pilot studies.^37 40 41^ Our modifications concern^31 71 72^: (a) strong standardisation of the supervision process, (b) verbalisation of the affective experience exclusively via ‘doubling’ (psychodrama technique) immediately after the role plays, (c) structured focussing on therapist‘s own contribution (vulnerabilities, biographical relationship experiences; based on pre work^72 73^) and (d) active role of supervisors in practising meta-communication (‘prompting’). The modifications were made because, in our experience, there is a tendency in supervision groups to rationalise, intellectualise and cognitively exchange concepts and strategies, which can also conceal difficulties in opening up affectively (resistance to the perception of subjective or objective countertransference^73 74^) or ‘hiding’ of alexithymic tendencies (ie, difficulties in perceiving and expressing emotions^75^). The modifications aim to enhance the focus on the perception and verbalisation of emotions, minimise intellectualising after role plays, and foster a more non-judgemental atmosphere within the supervision group. Supervision follows the following standardised procedure (duration approx. 45 min per patient): (A) Mindfulness exercise (to increase awareness and openness to one’s own emotional experience), 3–4 min; (B) Introduction of the patient and characteristics of the therapeutic relationship with this patient (B1: verbally, B2: video sequence from last therapy session, B3: ‘screenplay’ of an alliance rupture), 8–10 min; (C) First role-play and ‘doubling’ (C1: Supervisee in therapist role, supervision group member in patient role, C2: ‘Doubling’, psychodrama technique, to deepen the affective insight and enable access to blind spots), 7–9 min; (D) Second role-play and ‘doubling’ (D1: Supervisee in patient role, further supervision group member in therapist role, D2: ‘Doubling’, D3: Supervisor asks group members who were in the patient role how they felt), 10–13 min; (E) Third role-play and own contributions (E1: Supervisee in therapist role, tries out new behaviour, practises meta-communication, uses suggestions from the supervisor—prompting, E2: Supervisor asks what the rupture may have to do with supervisee’s vulnerabilities, whether own biographical issues may have been ‘triggered’, which feelings may be difficult to bear due to personal experiences), 6–8 min.

Supervisors belonging to MAFT-D will receive a 2-day workshop before the start of study therapies (same contents as for therapists but separately), followed by a monthly 2-hour online training to refine MAFT-D-specific supervision skills and discuss questions.

To ensure consistency across centres, MAFT-D therapists of different centres will be trained together. The same applies to supervisors. There will be a fixed group of lecturers (experienced psychotherapists and experienced in conducting MAFT-D-specific workshops and supervision).

### Sample size calculation, number of study participants

Depressive symptoms: There are no prior studies that closely align with our study in terms of study population, intervention and measurement instruments. In our view, the chosen reference Bambling *et al*^45^) represents the best available compromise, as it examines a population of depressed patients, employs an alliance-focused supervision approach and uses the BDI-II as a measurement instrument. Accordingly, we assume a higher reduction in BDIII after 20 weeks in MAFT-D compared with TAU of 4.0 BDI-II units with a SD of 7.3 BDI-II units. To detect such an effect with a type I error of .05 (two-sided hypothesis), a power of .90, an intracluster correlation coefficient of 0.05, and a cluster size of 2 patients per therapist on average, we need 76 therapists and 152 patients in total (38 therapists and 76 patients per group, PASS 15, tests for two means in a cluster-randomised design).

Treatment dropout: We expect that MAFT-D will reduce the treatment dropout rate after 20 weeks from 30% to 12.5%, corresponding to an OR of 3.0. The expected dropout rate of 30% for TAU is a conservative estimate based on a recent meta-analysis (35% dropout)^7^ and numbers in our own clinic (n=3.357 depressed patients; 33% dropout). Previous studies on different AFT approaches reported a difference in treatment dropout rates of 17.5% or greater (Bambling *et al*: IG1=6.1%, IG2=3%, CG=35%; Constantino *et al*: IG=0%, CG=27.3%; Muran *et al*: IG=20%, CG1=37%, CG2=46%).^45 46 76^ With identical design effects, 58 therapists and 116 patients per group (116 therapists, 232 patients in total) are needed to detect this difference (PASS 15, test for two proportions in a cluster-randomised design). We expect that 3% of the randomised sample are not part of the full analysis set (oriented on numbers from the trial of Schramm *et al*^77^) due to the lack of any data postrandomisation.^78^ Thus, based on the larger sample size required for dropout, a sample of 120 therapists and 240 patients in total has to be enrolled.

Based on previous psychotherapy trials,^77 79^ we expect a loss to follow-up of 15% (of therapists with their patients) at postassessment in BDI-II measurement. With the planned number of sample size and the assumed loss to follow-up, we can prove the assumed effect. We assume a non-compliance rate for the (per protocol, PP) BDI-II analysis (eg, failure to complete 12 sessions within 20 weeks, change in antidepressant medication, use of additional treatments) of 35%.

We expect that 20% of patients and therapists decline study participation, and 15% of therapists and 50% of patients do not meet inclusion criteria. Consequently, 178 therapists and 600 patients need to be initially invited in order to assess eligibility in 142 therapists and 480 patients, in order for 120 therapists and 240 patients to participate in the study and finally analyse 116 therapists and 232 patients. To ensure that the target sample size is achieved, up to five additional units may be recruited as a buffer (ie, 40 therapists, 80 patients, 10 supervisors). This precaution is necessary due to the interdependent nature of the units, where unexpected short-notice dropouts could compromise the participation of entire units.

### Endpoints, variables and instruments

Primary endpoints: Change in patient-rated depressive symptoms will be measured using the total score of (BDI-II, change=difference from baseline to 20 weeks after baseline),^66 80^ which is one of the most commonly used self-rating scales to assess depressive symptoms and is particularly recommended for monitoring the severity of depressive symptoms over time in clinical populations.^1^ Items are rated from 0 ‘not present’ to 3 ‘severe’. The total score is the sum of all 21 items (0–63). Good psychometric properties (reliability, validity, sensitivity to change) were demonstrated for the German version.^66^

Premature therapy dropout is a dichotomous variable determined by patient interview. It is defined as the patient’s decision to end therapy contrary to the initial agreement with the therapist, excluding premature terminations unrelated to therapy quality, such as those due to serious illness of the patient or therapist or moving to a different city. In case patients cannot be reached, interviews will be conducted with the therapist, and the patient is classified as a dropout.

#### Patient-related secondary endpoints

- Change in patient-rated depressive symptoms (BDI-II total score) and therapy dropout at the remaining measuring times.

Relating to the difference from baseline to 20 and 35 weeks, 20 and 36 months after baseline:

- Observer-rated depressive symptoms (Hamilton Rating Scale for Depression,^81 82^ total score of the 21-item version). Excellent inter-rater reliability (ICC, intraclass correlation coefficient=0.95), Cronbach’s α: 0.78.
- Patient-rated anxiety (Generalised Anxiety Disorder Scale-7,^83^ total score, rated on a 4-point Likert scale, 0 ‘not at all’ to 3 ‘nearly every day’). Cronbach’s α: 0.92, good test–retest reliability (ICC=0.83).^83^
- Patient-rated somatic symptoms (Patient Health Questionnaire-15,^84^ total score, ratings of problems in the last 4 weeks, 0=‘not bothered at all’ to 2 ‘very bothered’). Cronbach’s α: 0.80; validity was proven.^85^
- Patient-rated personality structure deficits (Operationalised Psychodynamic Diagnosis-Structure Questionnaire, short version,^86^ total score, 12 items assessing personality functioning, rated on a 4-point Likert scale, 0 ‘does not apply at all’ to 4 ‘does apply completely’). Cronbach’s α: 0.89.^86^
- Patient-rated interpersonal problems (Inventory of Interpersonal Problems-Revised, IIP-32,^87^ total score, 32 items scored on a 5-point Likert scale). Adequate internal consistency was demonstrated. Cronbach’s α of the subscales: 0.68–0.90.^88^
- Patient-rated quality of life (WHO Quality of Life, short version, WHOQOL–BREF,^89^ 5-point Likert scale, 1 ‘not at all’ to 5 ‘completely/to an extreme amount’). We will use two dimensions: physical and psychological quality of life. Cronbach’s α of the subscales: 0.57–0.88.

#### Therapist-related secondary endpoints

- Difference in observer-rated interpersonal skills from baseline to week 35 after baseline of the first patient (Facilitative Interpersonal Skills (FIS) Performance Test, FIS,^90^,^93^ total score, eight items measuring general interpersonal skills based on verbal responses to six video clips displaying challenging therapy situations—verbal fluency; hope; persuasiveness; emotional expression; warmth, acceptance and understanding; empathy; alliance bond capacity and rupture-repair responsiveness), 1 ‘low level’ to 5 ‘high level’ of skills). The German FIS version shows good inter-rater reliability (total score ICC=0.81). Cronbach’α: 0.95–0.96.^75 91^
- Therapist-rated retrospective satisfaction with supervision at week 20, 35, months 20, 36 after baseline of the first patient (modified version of the Client Satisfaction Questionnaire-8,^94^ total score, measuring satisfaction with supervision instead of treatment, CSQ-8-mod).^40^ Cronbach’α: 0.87–0.93, good concurrent validity for the total score.^95^

Relating to difference from baseline to 20 and 35 weeks, 20 and 36 months after baseline (all therapist rated):

- Trait-like relational manner and therapeutic competence score (relational manner—trait section (35 items) and skills change index section (10 items) of the Adapted Trainee Current Practice Report which is originally used in the SPRISTAD trial and currently undergoing psychometric evaluation^96^).
- Emotional suppression (Emotional Regulation Questionnaire subscale score,^97^ 4 items, 1 ‘totally disagree’ to 7 ‘totally agree’). Cronbach’s α suppression subscale: 0.74.
- Alexithymia (Toronto Alexithymia Scale,^98^ total score and the difficulties (a) identifying and (b) describing feelings subscales, 5-point Likert scale, 1 ‘does not apply at all or not true at all’ to 5 ‘completely true’). Cronbach’s α: 0.80–0.86.^98^

#### Variables to assess potential mediators for M-AFT-D effectiveness

- Average level of therapeutic alliance across sessions (German short revised version of the Working Alliance Inventory, WAI-SR,^99^,^101^ assessed after each session, 12 items). Cronbach’s α for the total score: >0.90. Convergent validity is established.^100^
- Convergence of patient’s and therapist’s alliance ratings over time (patient and therapist versions of the WAI-SR, total scores of session ratings; see above).
- Ratio of unresolved/resolved alliance ruptures (Post-Session-Questionnaire–modified version, PSQ-mod).^102 103^ The first item asks whether a ‘problem or tension’ occurred in the relationship between patient and therapist during the session. If answered with ‘yes’, the intensity of the rupture and the degree to which it could be solved is specified. Despite the simple nature of the items, there are clear indications of their validity.^102 103^
- Therapists’ usage of interventions referring to the therapeutic relationship in the here and now as well as usage of nondirective supportive techniques (mean of the categories ‘repeating, paraphrasing, summarising’, ‘expression of emotional sympathy’, ‘validation’); Psychodynamic Intervention List, PIL, observer-ratings based on transcribed therapy sessions on the level of verbal statements,^104^ 0 ‘category characteristics do not apply at all’ to 5 ‘category characteristics completely apply’. Satisfactory reliability and convergent validity; inter-rater reliabilities ICC ‘expression of emotional sympathy’ 0.69, ICC ‘validation’: 0.68, ICC ‘repeating, paraphrasing, summarising’: 0.80, ICC ‘referring to the therapeutic relationship’: 0.78, ICC topic ‘therapist’: 0.87.^56 104^
- Therapists’ empathy (Empathy Scale^105^), total score, 10-items, observer-ratings of video-taped sessions, 0 ’no agreement at all’ to 3 ‘very strong agreement’. Cronbach’s α: 0.86.^106^
- Therapists’ basic communication skills (Clinical Communication Skills Scale–short version;^107^ total score, 14 items, observer-ratings of video-taped sessions, 4-point scale ‘not at all appropriately’ to ‘entirely appropriately’). Reliability and validity were demonstrated.^107^
- Therapists’ adherence to AFT, CBT, PDT (BIFS^58^), observer-ratings of video-taped sessions, 12 items each (three subscales). Good psychometric properties, criterion validity and adequate inter-rater agreement have been demonstrated repeatedly.^108^

#### Variables for subsidiary research questions

- Mediators of change (patient-rated, Mediators of change in Psychotherapy Inventory^109^), after each session, 22 items and 5 subscales: quality of the therapeutic relationship (7 items), patients’ perception and expression of feelings and thoughts (5 items), patients’ becoming conscious of biographical experiences (4 items), gaining a new view on problems (4 items) and patients’ self-disclosure (2 items). A five-factor structure with good psychometric properties could be found.^109^
- Parenting behaviour of therapist’s parents (German translation of the Measure of Parental Style Questionnaire, Fragebogen dysfunktionaler elterlicher Beziehungsstile,^110 111^ parenting style of father and mother, 15 items each, 1 ’does not apply at all’ to 4 ’does completely apply’). Cronbach’s α: 0.48–0.93.^111^
- Therapists’ personality traits (Big Five Inventory, short version,^112^ measuring extraversion, agreeableness, conscientiousness, emotional stability, openness, 10 items). Cronbach’s α: 0.49–0.62.^113^
- Therapists’ interpersonal problems (IIP-32, see patient outcomes).
- Therapists’ sensitivity to aversive interpersonal behaviour (Interpersonal Sensitivities Circumplex German Questionnaire,^114^ 40 items assessing the extent to which individuals are bothered by others’ interpersonal behaviours, 1 ’not at all, never’ to 8 ’extremely, always’). Cronbach’s α: 0.74–0.89.
- Therapists’ abilities to mentalise (Certainty About Mental States Questionnaire,^115^ 20 items, 0 ‘never’ to 1 ‘always’, measuring the perceived capacity to understand mental states of the self and others). McDonald’s Ω: 0.88–0.91, good validity and test–retest reliability.^115^
- Therapist’s dispositional fear of negative evaluation (Fear of Negative Evaluation Scale, FNE,^116^ German version–Skala Angst vor negativer Bewertung,^117^ 5 items, 1 ‘almost never applies’ to 4 ‘almost always true’). Cronbach’s α: 0.84–0.94.
- Therapists: Relational manner–state,^96^ 35 items, see therapist-related endpoints.
- Therapists’ perception of own competence and skills (FIS, self-rating; see therapist-related endpoints).
- Patients’ therapy expectations and therapy evaluation, PATHEV,^118^ 11 items, 1 ’not true at all’ to 5 ’completely true’, three subscales: improvement, fear of change, suitability. Cronbach’s α: 0.73–0.89.
- Patients’ early depressive symptom changes (pre to fifth session, BDI-II,^66 80^ see above).
- Distinctive internal reactions (Impact Message Inventory,^119^,^121^ observer-ratings, 64 items, 1 ’not true at all’ to 4 ’absolutely true’, dimensions: affiliation and dominance. Cronbach’s α: 0.84–0.89, inter-rater reliability, ICC: 0.30–0.58.
- Planned for the following funding period: Rupture Resolution Rating System,^122^ observer ratings, presence of a rupture and its clarity and intensity, 5-point Likert scale. Inter-rater reliability withdrawal rupture ICC: 0.85, confrontation ruptures ICC: 0.98.^123^
- Selected sessions will be analysed in terms of nonverbal behaviour and linguistic variables.
- (Note: It would have been valuable to video-record not only the therapy sessions but also the supervision sessions, as this could have provided deeper insights into adherence to MAFT-D and the rupture-repair processes occurring during supervision. However, due to the already highly complex study design, we decided not to include this component in the current study.

### Measurement time points

The data collection is summarised in table 1.

**Table 1:** Data and measurement time points

Informant	Instrument	No. of items	Measurement points
Patient	BDI-II	21	Screening, BA, after the fifth therapy session, Post, FUP 1, 2, 3
	SCID-5-SPQ	106	Screening
	GAD-7	7	BA, Post, FUP 1, 2, 3
	PHQ-15	15	BA, Post, FUP 1, 2, 3
	OPD-SQS	12	BA, Post, FUP 1, 2, 3
	IIP-32	64	BA, Post, FUP 1, 2, 3
	WHOQOL-BREF	16	BA, Post, FUP 1, 2, 3
	PATHEV	11	BA
	Basic characteristics	app. 40	BA
	WAI-SR	12	After every session
	PSQ-Mod	3	After every session
	MOCPI	22	After every session
	Symptom severity VAS	1	After every session
	additional treatments	3	After every session
	INEP	21	Post, FUP 1, 2, 3
Observer interviews and ratings	SCID-5-CV	Adaptive	Screening
	SCID-5-PD	Adaptive	Screening or BA (depending on interview duration)
	adapted CSSRI	Adaptive	Screening, Post, FUP 1, 2, 3
	CSSR-S	23	Screening
	GRID-HAM-D-21	21	BA, Post, FUP 1, 2, 3
	Dropout-Interview	3	Flexibly
	BIFS-AFT	12	One session each of session 1-4/5-10, 11–16, 17–20 (if available)
	PIL	37	Sessions selected based on process data
	Empathy Scale	10	Sessions selected based on process data
	CCSS-S	14	Sessions selected based on process data
	IMI-R	64	Sessions selected based on process data
	3RS	22	Sessions selected based on process data
	FIS	6*9	BA, FUP1
Therapist	Basic characteristics, experience, practice	40	BA, Post, FUP 1, 2, 3
	Skills Change Index	10	BA, Post, FUP 1, 2, 3
	Relational Manner	35	BA, Post, FUP 1, 2, 3
	ERQ	10	BA, Post, FUP 1, 2, 3
	TAS-20	20	BA, Post, FUP 1, 2, 3
	FDEB	30	BA
	BFI-10	10	BA
	ISC-G	40	BA
	IIP-32	32	BA
	CAMSQ	20	BA
	SANB	5	BA
	FIS self-rating	9	BA, FUP 1
	CSQ-8-mod	8	Post, FUP 1, 2, 3
	WAI-SR	12	After every session
	PSQ-Mod	3	After every session
	Report on important events	Adaptive	flexibly
	Relational manner – state	35	After the fifth session
Supervisor	Basic characteristics, experience, practice	app. 10	BA
	Attendance sheet	4-5	After every supervision

BA, baseline assessment; BDI-II, Beck Depression Inventory–II^66 80^; BFI-10, Big Five Inventory–10^112^; BIFS, Beth Israel Fidelity Scale^58^; CAMSQ, Certainty about Mental States Questionnaire^115^; CCSS-S, Clinical Communication Skills Scale–short^107^; CSQ-8, Client Satisfaction Questionnaire-8, modified to supervision satisfaction^94^; CSSRI, Client Sociodemographic and Service Receipt Inventory^59^; CSSR-S, Colombia Suicide Severity Rating Scale^69^; ERQ, Emotion Regulation Questionnaire^97^; Empathy Scale^105^; FDEB, Fragebogen dysfunktionaler elterlicher Beziehungsstile^110^; FIS, Facilitative Interpersonal Skills^90^; FUP, follow up assessment; GAD-7, Generalised Anxiety Disorder Scale-7^83^; GRID-HAM-D, GRID Hamilton Rating Scale for Depression^81 82^; IIP-32, Inventory of Interpersonal Problems-32^87^; IMI-R, Impact Message Inventory^126^; INEP, Inventory for the assessment of Negative Effects of Psychotherapy^125^; ISC-G, Interpersonal Sensitivities Circumplex–German^114^; MOCPI, Mediators of Change in Psychotherapy Inventory^109^; OPD, operationalised psychodynamic diagnosis; OPD-SQS, OPD-Structure Questionnaire, short version^86^; PATHEV, Patientenfragebogen zur Therapieerwartung und Therapieevaluation^118^; PHQ-15, Patient Health Questionnaire-15^84^; PIL, Psychodynamic Interventions List^104^; Post, post-assessment; PSQ-Mod, Post-Session-Questionnaire, modified version^102 103^; Relational Manner–state and trait, and the Skills Change Index are both part of the Trainee Current Practice Report originally used in the SPRISTAD trial and currently under psychometric evaluation^127^; PSQ-Mod, Post-Session-Questionnaire, modified version^102 103^; 3RS, Rupture Resolution Rating System^123^; SANB, Skala Angst vor negativer Bewertung^128^; SCID-CV/PD, Structured Clinical Interview for DSM-5 Disorders, Clinician Version/ Personality Disorders^60 61^; TAS-20, Toronto Alexithymia Scale 20; VAS, Visual analogue scale^129^; WAI-SR, Working Alliance Inventory, short revised^100^; WHOQOL-BREF, WHO Quality of Life, short version^89^.

The number of items in the report on important events depends on which event (eg, study dropout, therapy dropout, hospitalisation) occurred. Important events should be reported as soon as possible, and the reporting is independent from scheduled sessions.

### Statistical analyses

Results are reported according to the Consolidated Standards of Reporting Trials (CONSORT) statement.^124^ Analyses are carried out with standard software: SPSS, V.29 or newer (IBM), SAS, V.9.4 or newer (SAS Institute), or R (V.4.3.3). A statistical analysis plan that contains the details of all planned analyses will be finalised before database lock.

Descriptive data are presented overall and separated by randomised group. As descriptive statistics, mean, SD, median, first and third quartile or minimum and maximum are calculated for continuous variables. For categorical variables, absolute and relative frequencies are reported.

First primary outcome (hypothesis I): We use a linear mixed model with repeated measures with changes from baseline as dependent variable, IG, therapeutic approach (CBT vs PDT), time and comorbid SCID5 personality disorder (yes vs no) as fixed effects, baseline BDI-II score as covariate, time by IG interaction, and cooperation centre, supervision group (if possible), therapist (=cluster) and patient as nested random effects. The interaction term is eliminated from the model if the corresponding interaction p value is >0.15. Measurement points up to and including the first follow-up (within the current funding period) will be considered. The contrast of the IG after 20 weeks is the result of the primary analysis. This result is considered in a confirmatory manner according to the approach described below. If the model fails to converge due to the complex cluster structure and variance components are estimated as zero, we will simplify the model stepwise by removing random effects.

Second primary outcome (hypothesis II): We perform a mixed logistic regression with the binary variable patient dropout after 20 weeks (yes vs no) as dependent variable, IG, therapeutic approach (CBT vs PDT) and comorbid SCID-5 personality disorder (yes vs no) as fixed effects, and cooperation centre, supervision group (if possible), and therapist as nested random effects.

The two primary hypotheses are hierarchically ordered and tested one after the other at the two-sided 5% level. Hypothesis II is only tested if hypothesis I could be rejected. If hypothesis I cannot be rejected, hypothesis II is no longer examined confirmatory but exploratory. This procedure ensures a family-wise type I error of 5%.

These analyses will be conducted either in the full analysis set, which is as complete as possible and as close as possible to the intention-to-treat ideal of including all randomised participants,^118^ or in the sample excluding randomised participants who did not receive a single dose (ie, one therapy session). Missing values are implicitly imputed by the chosen model. This allows for a (modified) intention-to-treat analysis, which results in unbiased estimations under the missing-at-random assumption. Missing values for treatment dropout can only be due to both patient and therapist leaving the study.

Sensitivity analysis for primary endpoints: To investigate the robustness of the results of the primary analyses, we will conduct sensitivity analyses with multiple imputation by chained equations as the imputation strategy. Additionally, primary analyses are repeated in the PP sample. Definition PP sample: patients who receive therapy by trained or non-trained and accordingly supervised therapists as intended by their group allocation without protocol deviations. Protocol violations include: (a) patient’s use of non-allowed medications (eg, newly prescribed antidepressant) or not allowed concurrent psychological treatments, (b) less than 12 completed therapy sessions at the end of week 20 (not relevant with respect to the outcome therapy dropout), (c) participants belonging to the IG, but not receiving the intervention in the intended dose, (d) non-compliant completion of assessments (eg, too early or too late). It will be documented (a) whether the protocol violation occurred during or after the study therapy or overlapping and (b) the measurement time point(s) for which the protocol violation is relevant.

Secondary outcomes: Secondary outcomes are examined exploratorily with analogous methods appropriate for the scale level (ie, mixed linear, logistic, ordinal, or Poisson/negative binomial regression). The contrast of the IG versus CG at week 35 of BDI-II changes from baseline of the model of the first primary outcome is used as a result of secondary outcome analysis. Analyses will be conducted up to and including the first follow-up (week 35) and will be repeated at a later time to include the subsequent measurement points (ie, follow-ups 2 and 3).

Mediators: In path models, we test the hypotheses that the patient’s outcome is mediated by the average level of therapeutic alliance across sessions (WAI patient-rated); ratio of unresolved/resolved alliance ruptures (PSQ-Mod patient-rated; categorised in case of low variance); convergence of the patient’s and therapist’s alliance ratings over time (WAI patient-rated/therapist-rated); therapists’ usage of interventions referring to the therapeutic relationship in the here and now; therapists’ usage of nondirective supportive techniques (validation, expressing emotional sympathy, repeating, paraphrasing, summarising; PIL); therapists’ competence in session (empathy); therapists’ adherence to AFT techniques (BIFS-Subscale AFT). We will test for the presence of collider bias in these mediators (eg, using directed acyclic graphs), and, if detected, apply appropriate statistical methods to address it.

Additional analyses: (a) To test whether MAFT-D is equally effective in CBT and PDT, we examine the interaction between IG and therapeutic approach. Prior to this analysis, we examine baseline differences in clinically and theoretically relevant variables (depression severity, chronicity, comorbidity) between therapeutic approaches (CBT/PDT). Variables for which we detect clinically relevant group differences will be added as covariates. Cooperation centre will be included as a random effect. (b) We examine the adherence measure BIFS (adherence with AFT-techniques, observer-rated) as an outcome, with the same approach as in primary analyses (extent of contamination bias). (c) Safety endpoints are determined using frequency tables and, if possible, (mixed) logistic or linear regressions to compare the event frequencies/groups. (d) Subsidiary research questions concern predictors, moderators, and/or mediators (patient-related, therapist-related, patient-therapist match-related and process-related variables) of (a) therapeutic macro and micro outcomes (eg, dropout, alliance), (b) the effectiveness of MAFT-D (eg, presence of a personality or therapeutic orientation as moderators of MAFT-D effectiveness), (c) observer-rated interpersonal therapeutic skills and skills development and (d) the successful resolution of alliance ruptures.

Interim analyses are not planned. For secondary outcomes and analyses, missing values are not imputed and no adjustment for multiple testing is conducted.

### Ethics and dissemination

The study is being conducted in compliance with the protocol, Good Clinical Practice, the Declaration of Helsinki and the CONSORT statement.^125^ All study patients, therapists and supervisors need to provide written informed consent to the main study team prior to their inclusion (see onlinesupplemental materials 1^4^: Example of Patient Consent Form; Example of Therapist Consent Form a/b; Example of Supervisor Consent Form). Study participation is completely voluntary and can be cancelled at any time without negative consequences. Patients who terminate study participation can continue their treatment. The study was approved by the Ethics Committee of the Psychologische Hochschule Berlin (EK2024/11). Additionally, we obtained secondary votes in each federal state where one or more study centres are located. Any major modifications to the study protocol will be covered by amendments and communicated to the relevant parties (eg, Data Safety and Monitoring Board, DSMB, see below). All personal data collected as part of the study, following the consent of the study participant, are subject to confidentiality and the provisions of the General Data Protection Regulation. All questionnaire, interview and rating data are collected pseudonymously within the secuTrial eCRF and REDCap surveys. This means that the participant’s name and all direct identifiers are replaced with a pseudonym, making reidentification outside of the study no longer possible. Access to the code key list that enables the association between study data and personal information of the study participant is limited to study staff explicitly authorised by the PI. The code key list is stored securely and separately from all other study data (eg, questionnaire data). The individual code key will be permanently erased following the completion of data collection and after the obligatory archiving time frame.

There is subject insurance for study patients. They receive the insurance confirmation and insurance conditions together with the study information. Assessments might be experienced as time-consuming. We do not expect other risks or adverse events due to study participation in patients or therapists. Positive effects resulting from treatments in both groups can be expected. There is no placebo or waiting list group. Both evidence-based treatments will be conducted according to state-of-the-art CBT/PDT and will be supervised by certified experienced psychotherapists. Frequency and dose of supervision correspond to usual standards.

Results will be published in peer-reviewed scientific journals following the ICMJE guidelines and disseminated to the general public, patient organisations and media.

Public involvement: Implications and recommendations for psychotherapy studies and further psychotherapy training are to be derived from the project results. These proposals will be discussed with relevant stakeholders (eg, the clinical directors of the training institutes).

### Quality assurance and safety

During the trial, quality control and quality assurance will be ensured through central monitoring based on the risk assessment of the study according to ICH Good Clinical Practice. The CTO will perform coordination, implementation and conduction of monitoring at the main study centre following its applicable Standard Operating Procedures (SOPs). Monitoring will be performed according to SOPs and study-specific requirements in accordance with the patient recruitment. The monitoring strategy will be defined in a monitoring plan with special attention to critical data and processes. The verification will focus on the key study data, for example, signed informed consent, adherence to inclusion and exclusion criteria and participant safety or adverse events. All the other data are checked on the basis of a representative sample, for example, documentation on primary objectives. Unclear and incomplete data will trigger increasing in-depth monitoring of the respective data.

Data management creates a data validation plan, containing additional logic checks for plausibility and consistency not available within the eCRF. Query analysis will be performed in SAS on an up-to-date export from the CRF. Found inconsistencies, implausible or missing data will be flagged by a data management query within the CRF. Possibly false information can be corrected through directly changing values within the eCRF or must be flagged as accepted failures or false positives. Changes within the documented information will be tracked via the audit trail, which is a core function of the eCRF.

After the last participant had its last visit, all queries and all centres were closed, and the database will be locked. Electronic documentation includes all exported files (SPSS, SAS, CSV, EXCEL), SAS scripts, data protocol and the closed database.

An independent DSMB will be regularly provided with all safety aspects of the trial and will review the safety data. Members of the DSMB are: Professor Dr Christiane Steinert, International Psychoanalytic University, Berlin, licensed psychotherapist; Professor Dr Elmar Brähler, Department of Psychosomatic Medicine and Psychotherapy, University Medical Center Mainz, experienced researcher and DSMB member in former psychotherapy trials and Dr Theresa Keller, Charité – Universitätsmedizin Berlin, independent statistician. At annual intervals, a meeting of the board will be scheduled to review whether the recruitment plan is on target, to ensure compliance with ethical principles, adherence to protocol, to check data quality and accuracy, and to advise whether to continue, modify or stop the trial. The DSMB will serve as a link to the funding organisation and provide them with information and advice. In the event of respective advice from the DSMB, the steering committee will finally decide whether to stop the trial (members: Professor A. Gumz, PI; Professor A. Zapf, statistician, Professor S. Singer—Institute for Medical Biostatistics, Epidemiology and Informatics, University Medical Center of the Johannes Gutenberg University Mainz, independent member and Professor L. White—Clinical Child and Adolescent Psychology and Psychotherapy, Bremen University, independent member).

Important events (eg, therapy dropouts, hospitalisation) can be reported by the therapist at any time. The study team will be alerted about incoming reports via email. Supervisors will be available at all study centres to decide about crisis interventions (eg, need for referrals). We will assess side effects/risks with questionnaires and interviews.

## Supplementary material

10.1136/bmjopen-2024-098343 online supplemental file 1

10.1136/bmjopen-2024-098343 online supplemental file 2

10.1136/bmjopen-2024-098343 online supplemental file 3

10.1136/bmjopen-2024-098343 online supplemental file 4
