# Supplementary material for: Modified Alliance-Focused Training with Doubling as an integrative approach to improve therapists’ competencies in dealing with alliance ruptures and prevent negative outcomes in psychotherapy for depression: study protocol of a randomised controlled multicentre trial
Source: BMJ Open. 2025 Jul 16;15(7):e098343. doi: 10.1136/bmjopen-2024-098343 (PMC12273124; doi:10.1136/bmjopen-2024-098343)
Supplement: online supplemental file 1 [file bmjopen-15-7-s001.docx]

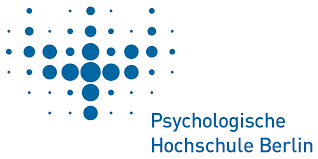
Prof. Dr. Antje Gumz

Professur für Psychosomatik und Psychotherapie

Psychologische Hochschule Berlin (PHB)

Am Köllnischen Park 2

10179 Berlin

**Prüfstelle:** Köln-Bonner Akademie für Verhaltenstherapie (KBAV), Wenzelgasse 35

53111 Bonn, Dr. phil. Lisa Miebach, ***************

**Zentrales Studienzentrum:** Professur für Psychosomatik und Psychotherapie, Psychologische Hochschule Berlin (PHB), Am Köllnischen Park 2, 10179 Berlin, a.gumz@phb.de

**Prüfer:** Prof. Dr. Antje Gumz

**Sponsor der klinischen Studie:** Psychologische Hochschule Berlin (PHB), Am Köllnischen Park 2, 10179 Berlin

DRKS number: DRKS00014842

**Studieninformation**

**Randomisiert kontrollierte Multicenter-Studie zur Therapieausbildung**

Projektnummer 504346851

Sehr geehrte Patientin, sehr geehrter Patient,

wir möchten Sie fragen, ob Sie bereit sind, an der nachfolgend beschriebenen klinischen Studie teilzunehmen.

Die klinische Studie, die wir Ihnen hier vorstellen, wurde gemäß der berufsrechtlichen Vorgabe (§15 der Berufsordnung für nordrheinische Ärztinnen und Ärzte) beraten, und erhebt keine berufsrechtlichen oder berufsethischen Bedenken und stimmt somit der Durchführung der Studie zu.

Diese klinische Studie wird an mehreren Orten und verschiedenen Psychotherapieausbildungsinstituten durchgeführt; es sollen insgesamt ungefähr 240 Patienten und 120 Therapeuten daran teilnehmen. Die Studie wird durch den oben genannten Sponsor veranlasst und finanziert.

**Ihre Teilnahme an dieser klinischen Studie ist freiwillig.** Sie werden in die Studie also nur dann einbezogen, wenn Sie dazu schriftlich Ihre Einwilligung erklären. Sofern Sie nicht teilnehmen oder später ausscheiden möchten, erwachsen Ihnen daraus keine Nachteile.

Der nachfolgende Text soll Ihnen die Ziele und den Ablauf erläutern. Der Text ist in drei Teile gegliedert:

- Kurzdarstellung der Studie.
- Teil I: Informationen zum Studienablauf einschließlich der damit zusammenhängenden gesundheitlichen Gesichtspunkte.
- Teil II: spezifische Informationen zum Datenschutz

Neben dieser schriftlichen Information hatten Sie bereits ein Aufklärungsgespräch mit einer oder einem Studienverantwortlichen an dem Institut, an dem Sie behandelt werden. Sie haben des Weiteren die Möglichkeit, ein persönliches Gespräch mit dem Studienteam in Berlin oder ggf. ein zweites Gespräch mit dem oder der Studienverantwortlichen an dem Institut, an dem Sie behandelt werden, vor Ort zu vereinbaren.

Bitte zögern Sie nicht, alle Punkte anzusprechen, die Ihnen unklar sind. Bei offen Fragen oder Schwierigkeiten können sie sich vor und während Studienteilnahme jederzeit an das Studienteam (unter [studie@phb.de](mailto:studie@phb.de)), die Studienleiterin, Prof. A. Gumz ([a.gumz@phb.de](mailto:a.gumz@phb.de)) oder die Studienverantwortlichen an dem Institut, an dem Sie behandelt werden sollen (Liste mit Namen und Kontaktdaten beiliegend) wenden. Sie können gerne Sätze/Abschnitte markieren, die Sie nicht verstanden haben, um sie mit dem Studienteam, der Studienleiterin oder der Ansprechpartnerin am Institut zu besprechen. Sie haben ausreichend Bedenkzeit, um über Ihre Teilnahme zu entscheiden

**Kurzdarstellung der Studie**

**Grund für die Studie**: Ein zu hoher Anteil an Patienten mit Depressionen profitiert nicht ausreichend von der Psychotherapie und viele brechen ihre Psychotherapie vorzeitig ab. Um die Ergebnisse von Psychotherapie weiter zu verbessern, ist es entscheidend, die Faktoren, die zum Erfolg beitragen, zu kennen und an ihnen anzusetzen. Ausgehend von bereits bekannten Einflussfaktoren, möchten wir in unserer Studie untersuchen, wie sich Veränderung in Psychotherapieprozessen ereignet, welches therapeutische Vorgehen und welche Patienten- und Therapeuteneigenschaften dazu beitragen. Wir prüfen, wie sich hilfreiche von weniger hilfreichen Therapiesitzungen unterscheiden und betrachten dabei Merkmale der therapeutischen Beziehungsgestaltung, die Eigenschaften der Therapeuten und Patienten, die angewandten Techniken sowie sprachliche und nonverbale Merkmale (z.B. Stimme oder Bewegungsverhalten). Zudem prüfen wir einen neuen Ausbildungsansatz für Therapeuten, der einen spezifischen Fokus auf einen der bekanntesten Einflussfaktoren für erfolgreiche Psychotherapien, die therapeutische Beziehung, legt. Dieser neue Ausbildungsansatz soll mit der regulären Therapieausbildung verglichen werden. In die Studie werden Patienten mit Depressionen, Therapeuten in Ausbildung und deren Supervisoren (Ausbilder) eingeschlossen. Mit den Ergebnissen dieser Studie erhoffen wir uns, einen Beitrag zur Verbesserung der Qualität der Therapieausbildung und der ambulanten Depressionsbehandlung zu leisten.

**Studienablauf:** An der klinischen Studie nehmen Patienten mit der Diagnose einer depressiven Störung, Therapeuten in Ausbildung und Supervisoren teil. Studienpatienten sind erwachsene Patienten mit einer depressiven Störung, die eine ambulante Psychotherapie (Verhaltenstherapie oder tiefenpsychologisch fundierte Therapie) beginnen möchten.

Wir werden vor Therapiebeginn prüfen, ob Sie die Kriterien für ein Studienteilnahme erfüllen (mit Fragebögen und einem Telefoninterview). Bei Eignung für die Studie findet eine weitere Erhebung mit Fragebögen und Telefoninterview statt. Anschließend werden Sie nach einem Zufallsprinzip (sogenannte Randomisierung) einer der beiden Studiengruppen zugeordnet (Gruppe 1: Therapie bei Therapeuten mit regulärer Therapieausbildung, Gruppe 2: Therapie bei Therapeuten, deren Ausbildung den neuen Ansatz enthält). Die Therapiesitzungen finden, wie üblich, wöchentlich für 50 Minuten statt. Die Dauer der Therapie (Kurzzeit- oder Langzeittherapie) legt Ihre Therapeutin bzw. Ihr Therapeut in Absprache mit ihrer Supervisorin bzw. ihrem Supervisor (Ausbilder) und Ihnen gemäß üblichem Vorgehen fest. Alle Therapiesitzungen werden videoaufgezeichnet. Nach jeder geplanten und durchgeführten Therapiesitzung füllen Sie einen Kurzfragebogen aus. Einmalig nach der 5. Woche ist der Fragebogen ein klein wenig länger. Die Kurzfragebögen können je nach Ihrer vorab angegebenen Vorliebe mit PC, Tablet oder auf Papier ausgefüllt werden. Nach 20 Wochen, 35 Wochen, 20 Monaten und 36 Monaten nach der Randomisierung findet eine weitere Erhebung mit Fragebögen und Telefoninterview statt. Die Fragebögen können im PC, Tablet oder auf Papier ausgefüllt werden. Die genannten studienbedingten Maßnahmen erfordern einen zusätzlichen Zeitaufwand von (ca. 6-8 Minuten) bei jedem Behandlungstermin. Auch bei Ihrer Therapeutin bzw. Ihrem Therapeuten werden über den Therapieverlauf hinweg Fragebogendaten erhoben.

**Möglicher Nutzen für Sie:**  Es ist zu erwarten, dass sich die Therapien in beiden Studiengruppen positiv auswirken. Beide evidenzbasierten Behandlungen werden nach üblichen Standards und nach dem neuesten Stand wissenschaftlicher Erkenntnisse durchgeführt und von zertifizierten erfahrenen Psychotherapeuten supervidiert. Wir erhoffen uns aufgrund bisheriger Forschungsergebnisse, dass ein neuer Ausbildungsansatz für Therapeuten im Vergleich zur regulären Psychotherapieausbildung die Therapieergebnisse der Patienten weiter verbessern kann. Das ist jedoch bisher nicht nachgewiesen. Viele Patienten finden es interessant, mit Hilfe von Fragebögen über die eigenen Muster der Beziehungsgestaltung, über sich selbst und über die Therapie und die erzielten Veränderungen nachzudenken. In jedem Falle können die Fragebögen und Interviews dabei helfen, die eigene Wahrnehmung zu schärfen und bestimmte Punkte, die mit einer erfolgreichen Therapie oft in Zusammenhang stehen, kontinuierlich unter die Lupe zu nehmen. Auch die an der Studie teilnehmenden Therapeuten füllen Fragebögen zu Ihnen und zur Therapie über den Therapieverlauf hinweg aus. Dies kann Ihre Therapie insgesamt unterstützen und intensivieren. Für Ihren Aufwand erhalten Sie eine Entschädigung von bis zu 280 € (inkl. MwSt.). Details zur Aufwandsentschädigung finden Sie unter I. 8.

Mit Ihrer Studienteilnahme unterstützen Sie Psychotherapieforschung, die langfristig möglicherweise dazu beiträgt, die ambulante Depressionsbehandlung zu verbessern.

**Risiken und Belastungen:** Die studienbedingten Erhebungen und die Datenerhebung können als zeitaufwändig empfunden werden. Das Nachdenken mit Hilfe von Fragebögen über die eigenen Muster der Beziehungsgestaltung, über sich selbst, die Therapie und die erzielten Veränderungen kann auch ein wenig anstrengend oder emotional aufwühlend erlebt werden. Wir erwarten keine weiteren Risiken oder unerwünschten Ereignisse aufgrund der Studienteilnahme.

**Freiwilligkeit:** Es ist Ihre freie Entscheidung, ob Sie an dieser Studie teilnehmen möchten oder nicht. Sie werden nur dann einbezogen, wenn Sie dazu schriftlich Ihre Einwilligung erkläre. Sie können jederzeit, auch ohne Angabe von Gründen, Ihre Einwilligung mündlich oder schriftlich widerrufen.

Zusätzlich zur schriftlichen Information wurden Sie mündlich aufgeklärt. Bei offen Fragen oder Schwierigkeiten können sie sich vor und während Studienteilnahme jederzeit an das Studienteam (unter [studie@phb.de](mailto:studie@phb.de)), die Studienleiterin, Prof. A. Gumz ([a.gumz@phb.de](mailto:a.gumz@phb.de)) oder die Studienverantwortlichen an Ihrem Institut (Liste mit Namen und Kontaktdaten beiliegend) wenden. Sie haben ausreichend Bedenkzeit, um sich für oder gegen eine Teilnahme zu entscheiden. Wenn Sie sich für die Teilnahme entscheiden, füllen Sie bitte die Einwilligungserklärung aus.

# **Teil I: Informationen zum Ablauf der klinischen Studie**

**I. 1. Warum wird diese Studie durchgeführt?**

Depressive Störungen gehören zu den häufigsten Erkrankungen. Der Verlauf der Erkrankung ist häufig wiederkehrend oder chronisch und die Folgen für den Einzelnen und die Gesellschaft können schwerwiegend sein. Verhaltenstherapien und tiefenpsychologisch fundierte Psychotherapien sind wissenschaftlich geprüfte, wirksame Therapieverfahren.

Trotz der generellen guten Wirksamkeit dieser Therapieverfahren, gibt es viele depressive Patienten, die nicht ausreichend von ihrer Psychotherapie profitieren und viele Patienten brechen ihre Psychotherapie vorzeitig ab. Damit noch mehr Patienten von einer Psychotherapie profitieren, ist es wichtig zu untersuchen, welche Faktoren zum Therapieerfolg beitragen. Ob eine Therapie wirksam ist, hängt von verschiedenen therapieprozessbezogenen, patientenseitigen und therapeutenseitigen Faktoren ab. Ein bekanntermaßen entscheidender Faktor ist, ob es gelingt, eine hilfreiche und vertrauensvolle Therapiebeziehung herzustellen und aufrechtzuerhalten.

Wir möchten in unserer Studie daher auch untersuchen, wie ein neuer Trainings- und Supervisionsansatz mit einem spezifischen Fokus auf der Herstellung oder Aufrechterhaltung einer guten therapeutischen Beziehung im Vergleich zur regulären Psychotherapieausbildung wirkt. Ob dieser Ansatz wirksam ist, prüfen wir anhand der Veränderung depressiver Symptome und der Anzahl vorzeitiger Therapieabbrüche. Wir erwarten, dass in der Gruppe mit dem neuen Trainingsansatz nach 20 Wochen, 35 Wochen, 20 Monaten und 36 Monaten, die depressiven Symptome stärker zurückgehen und weniger Therapien abgebrochen werden als in der Gruppe mit der üblichen Ausbildung. Zusätzlich prüfen wir die Veränderung weiterer psychischer Faktoren wie Ängstlichkeit, körperliche Beschwerden, zwischenmenschliche Probleme und Lebensqualität bei den Patienten sowie auf Therapeutenseite verschiedene Aspekte der therapeutischen Kompetenz und Zufriedenheit mit bestimmten Ausbildungselementen. Gleichzeitig erforschen wir, wie sich therapeutische Veränderung in den Therapieprozessen konkret ereignet, welches therapeutische Vorgehen und welche Patienten- und Therapeutenmerkmale mit besserem Therapieerfolg einhergehen. Wir prüfen, wie sich hilfreiche Sitzungen von weniger hilfreichen Sitzungen unterscheiden. Dabei betrachten wir Merkmale der therapeutischen Beziehungsgestaltung, Eigenschaften und angewandte Techniken der Therapeuten sowie sprachliche und nonverbale Merkmale (z.B. Stimme oder Bewegungsverhalten).

Entsprechend untersuchen wir in unserer Studie nicht nur Patienten. In unserer Studie wirken neben den Patienten auch Therapeuten in Ausbildung mit und deren Supervisoren (Ausbilder), die die Therapeuten, wie im Ausbildungskontext üblich, bei ihren Therapien unterstützen und beraten.

Von der Durchführung der vorgesehenen Studie erhoffen wir uns, einen Beitrag zur Qualität der Psychotherapieausbildung und hierüber zur Verbesserung der ambulanten Depressionsbehandlung zu leisten.

**I. 2. Kann ich mir aussuchen, welcher Studiengruppe ich angehören werde?**

Alle Patienten in der Studie erhalten wissenschaftlich geprüfte, wirksame Therapieverfahren (Verhaltenstherapien oder tiefenpsychologisch fundierte Psychotherapie). Im Rahmen der Studie wird ein neuer Psychotherapieausbildungsansatz verglichen mit der üblichen Psychotherapieausbildung. Zu diesem Zwecke findet eine Randomisierung statt. Sowohl die Studientherapeuten, als auch die Studienpatienten werden randomisiert. Das bedeutet, dass sie nach einem Zufallsverfahren einer von zwei Studiengruppen zugeordnet werden. Das Verfahren wird Randomisierung genannt. Es ist vergleichbar mit dem Werfen einer Münze. In der Studiengruppe 1 befinden sich Patienten, die eine Therapie bei Therapeuten bekommen, deren Ausbildung einen neuen Ausbildungsansatz enthält, in Gruppe 2 befinden sich Patienten, die eine Therapie bei Therapeuten bekommen, deren Ausbildung wie üblich erfolgt.

Zur objektiven Gewinnung von Studiendaten ist es notwendig, dass weder Sie noch die Studienmitarbeiterinnen, die in die Erhebung Ihrer Studiendaten einbezogen sind, wissen, in welcher Studiengruppe Sie sich befinden (dieses Verfahren wird als „Verblindung“ bezeichnet). Sollte es aus Sicherheitsgründen notwendig sein, kann unverzüglich festgestellt werden, welcher Studiengruppe Sie angehören.

**I. 3. Wie ist der Ablauf der Studie und was muss ich bei Teilnahme beachten?**

An der klinischen Studie nehmen erwachsene Patienten mit der Diagnose einer depressiven Störung, Therapeuten in Ausbildung und Supervisoren teil. Patienten mit einer depressiven Störung können teilnehmen, wenn sie eine ambulante Psychotherapie (Verhaltenstherapie oder tiefenpsychologisch fundierte Therapie) beginnen möchten.

Innerhalb einer Zeitspanne von 36 Monaten findet insgesamt sechs Mal eine Erhebung (mit Fragebogen und/oder Telefoninterview) statt. Zusätzlich werden nach jeder Therapiesitzung Fragebogendaten erhoben (ca. 6-8 Minuten).

Der Ablauf der Studie erfolgt in folgenden Schritten:

1. Im ersten Schritt bitten wir Sie nach dem Lesen dieser Informationen, die Einwilligungserklärungen zu unterzeichnen, wenn Sie mit einer Teilnahme einverstanden sind.

2. Anschließend prüft das Studienteam in einer Vorerhebung, ob Sie für die Studie geeignet sind. Füllen Sie dazu bitte die beiden beiliegenden Fragebögen **nach** dem Unterschreiben der Einwilligungserklärung aus. Sie schicken die Fragebögen sowie ein Exemplar der Einwilligungserklärung im vorbereiteten Umschlag an das zentrale Studienzentrum in Berlin oder Sie geben den Fragebogen im Umschlag bei der, dem Studienverantwortlichen an dem Institut, an dem Sie behandelt werden sollen, ab. Die Studienmitarbeiter prüfen anhand der Fragebögen, ob Sie für die Studie grundlegend geeignet sind. Bei Eignung wird das Studienteam Sie kontaktieren, um einen Termin für ein Telefoninterview zu vereinbaren. Das Studienteam kontaktiert Sie nur auf den Wegen, die Sie auf dem der Einverständniserklärung beiliegenden Formular als Möglichkeit angeboten haben. In dem Telefoninterview wird die Eignung für die Studie weiter geprüft. Wenn wir feststellen, dass Sie für die Studie nicht in Frage kommen sollten, kann es sein, dass das Interview nur relativ kurz dauern wird. Wenn Sie für die Studie in Frage kommen, dauert das Interview ungefähr eine bis maximal anderthalb Stunden. Von den Ergebnissen der Vorerhebung hängt ab, ob Sie weiter an dieser Studie teilnehmen.

2. Bei Studieneignung findet eine weitere Erhebung mit Fragebögen und einem weiteren etwas kürzeren Telefoninterview statt.

3. Anschließend findet die Randomisierung statt. Sie werden einer der beiden Studiengruppen zufällig zugeordnet. Nach der Zuordnung zu einer Studiengruppe werden Sie einem Studientherapeuten bzw. einer Studientherapeutin, der bzw. die derselben Studiengruppe angehört und der, die einen freien Therapieplatz hat zugeordnet. In seltenen Fällen kann es vorkommen, dass kein passender freier Therapieplatz mehr zur Verfügung steht. Sollte dies passieren, werden wir Ihnen und den Verantwortlichen am Institut schnellstmöglich Bescheid geben, so dass Sie einen Therapieplatz am Institut außerhalb der Studie erhalten können. Wenn die Vermittlung innerhalb der Studie klappt, verabredet die Studientherapeutin bzw. der Studientherapeut mit Ihnen, wann die erste Therapiesitzung stattfinden kann. Die Therapien finden, wie üblich, wöchentlich für 50 Minuten statt. Die Dauer der Therapie (Kurzzeittherapie oder Langzeittherapie) legt ihre Therapeutin bzw. ihr Therapeut in Absprache mit Ihnen und dem Studiensupervisor bzw. der Studiensupervisorin (Ausbilderin) gemäß üblichem Vorgehen fest.

4. Alle Therapiesitzungen werden videoaufgezeichnet. Ihre Therapeutin bzw. Ihr Therapeut wird für die Videoaufzeichnung und -speicherung verantwortlich sein. Die Videoaufzeichnung erfolgt bevorzugt mit einer Kamera. Ein anderes aufnahmefähiges Gerät (Handy oder Laptop) darf ausschließlich unter folgenden Bedingungen genutzt werden: Die Geräte dürfen während der Aufnahme keine Verbindung zum Internet haben und die Verbindung zum Internet darf erst wiederhergestellt werden, wenn die Therapiesitzung auf die hierfür vorbereiteten gesicherten, verschlüsselten Datenträger überspielt wurde und auf dem Gerät vollständig gelöscht wurde. Die Speicherung und Übertragung der Videos, die für unsere Studie verwendet und ausgewertet werden, erfolgt durch Ihren Studientherapeuten an einem Computer des Instituts. Auf diesem Computer hat das zentrale Studienteam einen privaten Tunnel (SSL-VPN) eingerichtet. Das Video wird in verschlüsselter Form über diesen privaten Tunnel an die Studienzentrale der Psychologischen Hochschule Berlin (PHB) übertragen. Die VPN-Verbindungen werden vom Studienteam vorbereitet, Ihre Therapeutin bzw. Ihr Therapeut erhält ein individuelles Passwort. Die Videoaufzeichnungen können von den Therapeuten in der Supervision genutzt werden. Diese werden auf verschlüsselten externen Datenträgern gespeichert und nach der Nutzung vollständig gelöscht.

Es ist ein übliches Vorgehen in Psychotherapien, dass Therapeuten (auch erfahrene Therapeuten) sich von erfahrenen Kollegen supervidieren lassen. Supervisionen tragen zur Qualität der Therapien bei. Supervision bedeutet, dass die Therapiesitzungen regelmäßig im Kreis von Fachkollegen besprochen werden, um mögliche Probleme oder Hindernisse frühzeitig zu erkennen und über deren Lösung zu beraten.

Einige Sitzungen aus Ihrem Therapieverlauf werden zusätzlich für Forschungszwecke genutzt. Anhand dieser Sitzungen werden wissenschaftliche Mitarbeiter unserer Forschungsgruppe, die der Verschwiegenheit verpflichtet sind, zum einen einschätzen, inwieweit Therapeuten ihr Vorgehen im Sinne des neuen Ansatzes ausgerichtet haben, d.h., inwieweit sie anders vorgehen als die Therapeuten der Studiengruppe mit üblicher Therapieausbildung. Dies dient der Einschätzung der Qualität der Intervention und der Studie. Außerdem möchten wir einzelne Sitzungen auf Basis der Fragebogenantworten von Ihnen und Ihrer Therapeutin bzw. Ihrem Therapeuten auswählen und Merkmale der therapeutischen Beziehungsgestaltung, therapeutische Eigenschaften und Techniken sowie sprachliche und nonverbale Merkmale (z.B. Stimme oder Bewegungsverhalten) untersuchen. Diese Erhebungen dienen dazu, unsere Fragestellungen zu Einflussfaktoren auf den Therapieerfolg zu beantworten. Die Videoaufzeichnungen können zu Beginn ungewohnt sein. Erfahrungsgemäß setzt ein Gewöhnungseffekt recht schnell innerhalb der ersten Stunden ein.

5. Nach jeder Therapiesitzung füllen Sie einen Kurzfragebogen aus (6 bis 8 Minuten). Einmalig nach der 5. Woche bitten wir Sie, einen zusätzlichen Fragebogen auszufüllen (ca. 5 Minuten). Diesen können Sie über Ihr Smartphone ausfüllen. Sie erhalten zu Beginn der Therapie einen Link von Ihrem Studientherapeuten über den Sie den Fragebogen erreichen können.

6. Jeweils 20 Wochen, 35 Wochen, 20 Monate und 36 Monate nach der Randomisierung findet eine weitere ausführlichere Erhebung mit Fragebögen (ca. 30-50 Minuten) und ein Telefoninterview (ca. 15-20 Minuten) statt. Mit Ihrer Einwilligung werden Sie an die Termine vorab erinnert (2 Wochen vorher). Diese Erhebungen finden unabhängig von den Therapiesitzungen statt und Sie können sie je nach Ihrer Präferenz online oder auf Papier ausfüllen. Sie können die Erhebungen an einem Ort Ihrer Wahl durchführen. Wir bitten Sie sicherzustellen, dass eine ruhige Atmosphäre herrscht und Sie für die Dauer der Erhebungen ungestört sind. Die Erhebungen mittels Fragebögen finden unabhängig von Ihrer Therapie statt. Das heißt, auch wenn Ihre Therapie abgeschlossen ist, werden die Befragungen fortgeführt.

Auch die Studientherapeuten nehmen über den Therapieverlauf hinweg an einer Fragebogenerhebung teil.

Wichtig ist zu ergänzen: **Die Therapeuten erhalten keinen Einblick in die Daten der Patienten und die Patienten erhalten keinen Einblick in die Daten der Therapeuten! Die einzige Ausnahme davon ist, wenn eine akute Gefahr für Selbst- oder Fremdgefährdung festgestellt werden sollte.**

**I. 4. Welchen persönlichen Nutzen habe ich von der Teilnahme an der Studie?**

Wir erhoffen uns aufgrund bisheriger Forschungsergebnisse, dass ein neuer Ausbildungsansatz im Vergleich zur regulären Psychotherapieausbildung die Therapieergebnisse der Patienten zusätzlich verbessern kann. Das ist jedoch bisher nicht nachgewiesen. Wenn Sie bei einer Therapeutin bzw. einem Therapeuten in Behandlung sein werden, die bzw. der mit der neuen Methode ausgebildet wird, so kann dies möglicherweise dazu beitragen, dass Ihre Therapien erfolgreicher verlaufen. Da die Wirksamkeit der Methode noch nicht erwiesen ist, ist es jedoch auch möglich, dass Sie durch Ihre Teilnahme an dieser Stelle keinen erhofften Nutzen haben. Es ist zu erwarten, dass sich die Therapien in beiden Studiengruppen positiv auswirken. Beide evidenzbasierten Behandlungen werden nach üblichen Standards und nach dem neuesten Stand wissenschaftlicher Erkenntnisse durchgeführt und von zertifizierten erfahrenen Psychotherapeuten supervidiert.

Viele Patienten finden es interessant, mit Hilfe von Fragebögen über die eigenen Muster der Beziehungsgestaltung, über sich selbst und über die Therapie und die erzielten Veränderungen nachzudenken. In jedem Falle können die Fragebögen und Interviews dabei helfen, die eigene Wahrnehmung zu schärfen und bestimmte Punkte, die mit einer erfolgreichen Therapie oft in Zusammenhang stehen, kontinuierlich unter die Lupe zu nehmen. Auch die an der Studie teilnehmenden Therapeuten füllen Fragebögen zu Ihnen und zur Therapie über den Therapieverlauf hinweg aus. Dies kann Ihre Therapie insgesamt unterstützen und intensivieren. Für Ihren Aufwand erhalten Sie eine Entschädigung von bis zu 280 €. Details zur Aufwandsentschädigung finden Sie unter I. 8.

Mit Ihrer Studienteilnahme unterstützen Sie Psychotherapie- und Ausbildungsforschung, die langfristig möglicherweise dazu beiträgt, die ambulante Depressionsbehandlung zu verbessern.

1. **5. Welche gesundheitlichen Risiken und Belastungen sind mit der Teilnahme an der Studie verbunden?**

Die studienbedingten Erhebungen und die Datenerhebung können als zeitaufwändig empfunden werden. Das Nachdenken mit Hilfe von Fragebögen über die eigenen Muster der Beziehungsgestaltung, über sich selbst, die Therapie und die erzielten Veränderungen kann auch ein wenig anstrengend oder emotional aufwühlend erlebt werden. Wir erwarten keine weiteren Risiken oder unerwünschten Ereignisse aufgrund der Studienteilnahme.

**I. 6. Welche anderen Behandlungsmöglichkeiten gibt es außerhalb der Studie?**

Wenn Sie sich entscheiden, nicht an der Studie teilzunehmen oder die Studie abzubrechen, hat dies keine negativen Konsequenzen für Ihre Psychotherapie.

**I. 7. Wer darf an dieser klinischen Studie nicht teilnehmen?**

Sie können an dieser klinischen Studie nur teilnehmen, wenn Sie nicht gleichzeitig für andere klinische Studien oder Forschungsprojekte zur Verfügung stellen*.*

**I. 8. Entstehen für mich Kosten durch die Teilnahme an der klinischen Studie? Erhalte ich eine Aufwandsentschädigung?**

Durch Ihre Teilnahme an dieser Studie entstehen für Sie keine Kosten*.* Für Ihre Teilnahme an dieser Studie erhalten Sie eine kleine Aufwandsentschädigung entsprechend den folgenden Bedingungen:

- Patienten, die an der Erhebung zum Prüfen der Eignung für die Studie teilgenommen haben (Fragebögen und Telefoninterview) und aufgrund fehlender Eignung nicht in die Studie aufgenommen werden können, erhalten 30 €.
- Patienten, die an den Erhebungen bis einschließlich zur 20. Woche teilnehmen, erhalten weitere 150€.
- Patienten, die an den Erhebungen bis zur 35. Woche teilnehmen, erhalten weitere 50€.
- Patienten, die an den Erhebungen bis zum 20. Monat teilnehmen, erhalten weitere 50 €.

Dies entspricht einer maximalen Gesamtaufwandsentschädigung von **280 €** bei Teilnahme an den Erhebungen bis zum 20. Monat.

- Patienten, die an den Erhebungen bis zum 36. Monat teilnehmen, erhalten eine weitere Summe, die erst zum späteren Zeitpunkt konkretisiert werden kann (nach Beantragung der Fortführung der Studienförderung).

**I. 9. Bin ich während der klinischen Studie versichert?**

Bei der klinischen Studie sind Sie versichert. Es handelt sich um eine Probandenversicherung mit zusätzlicher Wegeunfallversicherung (Schadensersatzhöhe
Probandenversicherung: € 500.000 für die einzelne versicherte Person, Wegeunfallversicherung (lediglich direkte Wege zur und von der Therapie): € 100.000 bei Invalidität, € 100.000 bei Tod.

Versicherungszeitraum: 01.07.2024-01.07.2027

Der Umfang des Versicherungsschutzes ergibt sich aus den Versicherungsunterlagen, die Sie im Anhang dieser Studieninformation finden. Wenn Sie vermuten, dass durch die Teilnahme an der klinischen Studie Ihre Gesundheit geschädigt oder vorher bestehende Leiden verstärkt wurden, müssen Sie dies unverzüglich dem Versicherer innerhalb einer Woche direkt anzeigen, gegebenenfalls mit Unterstützung durch die Studienleiterin, um Ihren Versicherungsschutz nicht zu gefährden.

**Name und Anschrift der Versicherung**:

QBE Europe SA/NV

Direktion für Deutschland
Breite Str. 31
40213 Düsseldorf

Sofern die Studienleiterin Sie dabei unterstützt, erhalten Sie eine Kopie der Meldung. Sofern Sie Ihre Anzeige direkt an den Versicherer richten, informieren Sie bitte zusätzlich Ihre Studienleiterin.

Bei der Aufklärung der Ursache oder des Umfangs eines Schadens müssen Sie mitwirken und alles unternehmen, um weiteren Schaden abzuwenden.

Während der Dauer der klinischen Studie dürfen Sie sich einer anderen medizinischen Behandlung – außer in Notfällen – nur nach vorheriger Rücksprache mit dem Studienteam und der Studienleiterin unterziehen. Von einer erfolgten Notfallbehandlung müssen Sie das Studienteam und die Studienleiterin unverzüglich unterrichten.

Sie erhalten ein Exemplar der Versicherungsbestätigung einschließlich der Versicherungsbedingungen. Bitte beachten Sie die Pflichten, die Sie im eigenen Interesse beachten müssen, um Ihren Versicherungsschutz zu erhalten.

**I. 10. Werden mir neue Erkenntnisse zu der klinischen Studie mitgeteilt?**

Sie werden während Ihrer Teilnahme über neue Erkenntnisse in Bezug auf diese Studie informiert, die für Ihre Bereitschaft zur weiteren Teilnahme wesentlich sein können.

# Nach Beendigung der gesamten Studie wird das Studienteam zusammenfassende Ergebnisse in der Datenbank des Deutschen Registers Klinischer Studien (https://drks.de/search/de/results) bereitstellen. Dies kann von Ihnen unter der oben angegebenen trial number eingesehen werden. Sie können sich nach Studienende zur Information über die Studienergebnisse zudem gerne an das Studienteam wenden.

**I. 11. Wer entscheidet, ob ich aus der klinischen Prüfung ausscheide?**

**Sie können jederzeit, auch ohne Angabe von Gründen, Ihre Teilnahme beenden, ohne dass Ihnen dadurch Nachteile entstehen.**

Es ist auch möglich, dass der Sponsor entscheidet, Ihre Teilnahme an der klinischen Studie vorzeitig zu beenden. Ein möglicher Grund dafür kann sein, dass die gesamte klinische Studie abgebrochen werden muss (z.B. falls ein Zusammenhang zwischen dem Vorkommen schwerwiegender unerwünschter Ereignisse und der Studienteilnahme festgestellt wird).

**I. 12. An wen wende ich mich bei weiteren Fragen?**

Bei offen Fragen oder Schwierigkeiten können sie sich vor und während Studienteilnahme jederzeit an das zentrale Studienzentrum ([studie@phb.de](mailto:studie@phb.de)), die Studienleiterin Prof. A. Gumz ([a.gumz@phb.de](mailto:a.gumz@phb.de)) oder die Studienverantwortlichen an Ihrem Institut (Liste mit Namen und Kontaktdaten beiliegend) wenden.

**Teil II: Informationen zum Datenschutz**

**II. 1. Was geschieht mit den über mich erhobenen Daten?**

### a) Allgemeine Informationen

Während der klinischen Studie werden persönliche Informationen von Ihnen erhoben und ggf. kurzfristig im lokalen Studienzentrum (d.h. in Ihrem Institut) in verschlossenen Umschlägen und dauerhaft im zentralen Studienzentrum (an der Psychologischen Hochschule Berlin) gesichert gelagert (in verschlossenen Schränken). Abgesehen von der Einwilligungserklärung, die Ihren Namen und weitere persönliche Informationen enthält, werden alle anderen Studiendaten nur in pseudonymisierter Form erhoben, verarbeitet und gespeichert. Pseudonymisiert bedeutet, dass keine Angaben, mit denen Sie direkt identifiziert werden können (z.B. Namen, Kontaktinformationen, Geburtsdatum, etc.) verwendet werden, sondern nur ein Nummern- und Buchstabencode*.* Dieser Code, d.h. die Patienten-ID, besteht aus zehn Zeichen und setzt sich folgendermaßen zusammen: a) 2 Ziffern, die das Institut, an dem Sie behandelt werden, kennzeichnen; b) P (für Patient/in); c) eine fortlaufende vierstellige Ziffer beginnend mit 1001; d) die ersten zwei Buchstaben des Vornamens der Mutter, und e) der erste Buchstabe Ihres Geburtsortes. **Wir bitten Sie, die letzten drei Stellen (d und e) auf den beiliegenden Fragebögen dementsprechend zu vervollständigen.**

**Bitte notieren oder merken Sie sich Ihren Code.** Alle weiteren Studienunterlagen, die für Sie persönlich bestimmt sind, werden nur diesen Code enthalten.

Die Liste mit der Zuordnung („Schlüssel“) der Studiencodes zu den tatsächlichen Identitäten, wird während der Studienlaufzeit und bis zu zehn Jahre nach Ende der Studienlaufzeit im zentralen Studienzentrum (Psychologische Hochschule Berlin, PHB) gesichert (d.h. elektronisch verschlüsselt oder im Fall von physischen Kopien in einem verschlossenen Schrank) aufbewahrt. Diese Zuordnungsliste wird jederzeit getrennt von allen anderen Studiendaten gelagert. Die schriftliche Kommunikation zwischen den lokalen Studienzentren (Ausbildungsinstitut, an dem Sie behandelt werden) und dem zentralen Studienteam (PHB) erfolgt ausschließlich über die Studiencodes. In definierten Ausnahmefällen (sollte eine akute Selbst- oder Fremdgefährdung im Rahmen der Studie festgestellt werden, bei Schwierigkeiten bei der Datenerhebung) werden Namen in der Kommunikation zwischen lokalen Studienverantwortlichen und zentralem Studienteam verwendet. Dies geschieht jedoch ausschließlich über telefonische Kontakte und um die Pseudonymisierung zu erhalten ausnahmslos ohne Nennung von Studiencodes.

Zugang zu dem „Schlüssel“, der eine persönliche Zuordnung des Studienteilnehmers, der Studienteilnehmerin ermöglicht, haben nur von der Studienleiterin Prof. Dr. Antje Gumz ausdrücklich dazu autorisierte Projektmitarbeiter, die zum Kernstudienteam gehören und der Schweigepflicht unterliegen. Eine Entschlüsselung erfolgt nur, wenn das Studienteam aus spezifischen studienbezogenen Gründen Kontakt zu Ihnen aufnehmen muss (z.B. zur Erinnerung an studienbezogene Erhebung mit Ihrer Einwilligung, zur Zusendung Ihrer Zugänge zu den Fragebogenerhebungen, für eine Kurzbefragung bei Therapie- und Studienabbruch mit der Einwilligung von Ihnen).

Der individuelle Schlüssel wird zehn Jahre nach Ende der Studienlaufzeit dauerhaft gelöscht. Die sichere Aufbewahrung des Schlüssels über diese Zeit hinweg ist nötig, damit das Studienteam sicherheitsrelevante Daten auch nach dem Ablauf der Studie noch zuordnen kann.

Trotz der Pseudonymisierung lässt sich niemals völlig ausschließen, dass auch ohne den Schlüssel Rückschlüsse auf Ihre Person gezogen werden können. Dies trifft insbesondere auf die Videoaufzeichnungen der Sitzungen zu.

Die videoaufgezeichneten Therapiesitzungen werden von Ihrer Therapeutin bzw. Ihrem Therapeuten wöchentlich verschlüsselt über eine gesicherte Tunnelverbindung (VPN) per Internet an das Rechenzentrum (RZ) der Psychologischen Hochschule Berlin (PHB) verschickt und dort gespeichert. Die VPN-Verbindungen werden vom Studienteam vorbereitet, Ihre Therapeutin bzw. Ihr Therapeut erhält ein individuelles Passwort. Die für die Supervision genutzten Videos, wird Ihr Therapeut an dem zentralen Computer am Institut mit Vera-Crypt verschlüsseln und einzelne Sitzungen auf externen Speicherträgern speichern und für die Supervision nutzen. Nach der Supervision werden die einzelnen Sitzungen durch Ihren Therapeuten von dem verschlüsselten Speicherträger vollständig gelöscht. Sollte die technische Ausstattung zur Videoaufzeichnung (Kamera, ggf. Stativ) an dem Institut, an dem Sie behandelt werden, nicht zur Verfügung gestellt werden können, dürfen andere aufnahmefähige Geräte (Handy oder Laptop) genutzt werden, allerdings ausschließlich unter folgenden Bedingungen: Die Geräte dürfen während der Aufnahme keine Verbindung zum Internet haben und die Verbindung zum Internet darf erst wiederhergestellt werden, wenn die Therapiesitzung auf dem Laptop bzw. Handy vollständig gelöscht wurde. Zugriff auf die Videodaten im RZ der PHB hat nur Ihre Therapeutin bzw. Ihr Therapeut sowie von der Studienleiterin Prof. Dr. Antje Gumz ausdrücklich dazu autorisierte Studienmitarbeiter, die die der Schweigepflicht unterliegen. Weiterhin Mitarbeiter der IT der PHB, die ebenfalls der Schweigepflicht unterliegen. Einzelne Sitzungen aus den Therapieverläufen werden von diesen ausgewählten Studienmitarbeitern ausgewertet. Jedes zur Auswertung bestimmte Video wird von einer Studienmitarbeiterin auf identifizierende Informationen (z.B. Nennung von Namen) geprüft und diese werden entfernt. Diese Entfernung von identifizierenden Informationen übernimmt diejenige Studienmitarbeiterin, die bereits Zugang zu Ihren persönlichen Daten im Rahmen der Durchführung von den Telefoninterviews hatte, sofern dies möglich ist. Zur Auswertung der Videositzungen werden Ratinginstrumente, phonetische, linguistische und Bewegungsmuster-Analysen genutzt. Für die jeweilige Auswertung wird die Datengrundlage genutzt, die Ihre Anonymität am besten schützt. Beispielsweise werden für spezifische Auswertungen lediglich vollständig pseudonymisierte Transkripte der Videositzungen verwendet. Einige Auswertungen beruhen jedoch auch auf Mimik, Gestik und anderen non-verbalten Merkmalen, so dass das Video notwendig ist. Die Ergebnisse dieser Auswertungen werden ausschließlich pseudonymisiert gespeichert.

Die für die Supervision genutzten Videos, die auf verschlüsselten externen Datenspeichern genutzt werden, werden nach der Supervision vollständig gelöscht. Die im RZ der PHB verschlüsselt gespeicherten Videoaufzeichnungen werden während der Studienlaufzeit und bis maximal 10 Jahre nach Ende der Studienlaufzeit so wie der oben beschriebene Datenschlüssel in einem verschlossenen Schrank aufbewahrt und anschließend vollständig vernichtet.

### b) Rechtsgrundlage

Rechtsgrundlage für die Datenverarbeitung ist Ihre informierte Einwilligung gemäß Art. 6 Abs. 1 Buchst. a und Art. 9 Abs. 2 Buchst. a der EU-Datenschutzgrundverordnung (DSGVO).

Die Bereitstellung Ihrer personenbezogenen Daten ist freiwillig. Ohne Ihre ausdrückliche Einwilligung in die Verarbeitung Ihrer Daten können Sie allerdings nicht an dieser klinischen Studie teilnehmen.

### c) Verantwortlichkeit

Verantwortlich im Sinne des Datenschutzrechts ist Prof. Dr. A. Gumz (Prüfer/Sponsor).

Das lokale Studienzentrum (d.h. das Institut, an dem Sie behandelt werden) bleibt davon unabhängig für Ihre Behandlungsdaten verantwortlich (unkodierte Patientendaten).

### d) Zweck(e)

Mit Hilfe der erhobenen Daten soll die Wirksamkeit eines neuen Trainings- und Supervisionsansatzes mit einem spezifischen Fokus auf der therapeutischen Beziehung im Vergleich zur regulären Psychotherapieausbildung für Therapeuten in Ausbildung und die von ihnen behandelten Patienten mit einer depressiven Störung klinisch untersucht werden. Gleichzeitig erforschen wir auch, wie sich therapeutische Veränderung in den Therapieprozessen konkret ereignet, welches therapeutische Vorgehen und welche Patienten- und Therapeutenmerkmale mit besserem Therapieerfolg einhergehen. Wir prüfen, wie sich hilfreiche Sitzungen von weniger hilfreichen Sitzungen unterscheiden. Dabei betrachten wir Merkmale der therapeutischen Beziehungsgestaltung, Eigenschaften von Therapeuten und Patienten, angewandte Techniken sowie sprachliche und nonverbale Merkmale (z.B. Stimme oder Bewegungsverhalten).

### e) Weitergabe/Empfänger

Die von Ihnen erhobenen Daten werden, soweit erforderlich, pseudonymisiert weitergegeben an:

1. vom Prüfer/Sponsor beauftragte Stellen zum Zweck der Durchführung und wissenschaftlichen Auswertung,
2. im Falle unerwünschter Ereignisse: vom Prüfer/Sponsor an zuständige Überwachungsbehörden im Rahmen von Inspektionen oder Beauftragte des Prüfers/Sponsors (s.g. Auditoren oder Monitore)

Die von Ihnen im Rahmen der oben genannten klinischen Studie erhobenen und gespeicherten Daten (auch die originalen Klardaten) können soweit erforderlich und gesetzlich erlaubt, durch Beauftragte des Sponsors/zentralen Studienzentrums (s.g. Monitore) zur Überprüfung der ordnungsgemäßen Durchführung der klinischen Studie im zentralen Studienzentrum eingesehen werden. Diese sind zur Vertraulichkeit verpflichtet, eine Weitergabe der erhobenen Daten erfolgt in diesem Zusammenhang nicht.

Im Rahmen dieser klinischen Studie erfolgt eine Weitergabe Ihrer pseudonymisierten Daten zum Zweck der Datenauswertung, Zulassung und Überwachung nur innerhalb der Europäischen Union und des Europäischen Wirtschaftsraumes.

### f) Ihre Rechte

Sie haben grundsätzlich folgende Rechte bezüglich Ihrer personenbezogenen Daten, sofern dies nicht aufgrund einer zwischenzeitlich vorgenommenen Löschung der identifizierenden Merkmale zur Entschlüsselung technisch oder anderweitig gesetzlich unmöglich ist:

**Recht auf Widerruf ihrer Einwilligung**

So wie die Einwilligung zur Teilnahme an der klinischen Studie können Sie auch Ihre Einwilligung zur Verarbeitung der erhobenen Daten jederzeit widerrufen.

Im Falle eines Widerrufs Ihrer Einwilligung werden Ihre Daten unverzüglich gelöscht.

**Sie haben weiterhin folgende Rechte**

Recht auf Auskunft (inkl. unentgeltlicher Überlassung einer Kopie) über Ihre personenbezogenen Daten, die im Rahmen der klinischen Studie erhoben, verarbeitet oder ggf. an Dritte übermittelt werden.

Recht auf Datenübertragung der zu Ihrer Person erhobenen Daten an Sie oder eine von Ihnen bestimmte Stelle.

Recht auf Berichtigung unrichtiger personenbezogener Daten, auf Einschränkung der Verarbeitung und auf Widerspruch gegen die Nutzung der Daten.

**Wahrnehmung Ihrer Rechte**

Wollen Sie von einem oder mehreren der genannten Rechten Gebrauch machen, kontaktieren Sie bitte Ihren Prüfer/Sponsor. Bei Anliegen zur Datenverarbeitung und zur Einhaltung der datenschutzrechtlichen Anforderungen können Sie sich auch an folgende Datenschutzbeauftragte wenden:

Datenschutzbeauftragter des zentralen Studienzentrums / Sponsors:

Herr Marko Walther,

Psychologische Hochschule Berlin

Am Köllnischen Park 2

10179 Berlin

*************

Sie haben außerdem ein Beschwerderecht bei einer Datenschutzaufsichtsbehörde. Sollten Sie Bedenken hinsichtlich des Umgangs mit Ihren personenbezogenen Daten haben, können Sie sich an folgende Stellen wenden:

DSB Münster GmbH

André Korte

Martin-Luther-King-Weg 42-44

48155 Münster

Tel.: +49 251 71879-110

E-Mail: **************

Eine Liste aller in Deutschland und der Europäischen Union zuständigen Datenschutzaufsichtsbehörden finden Sie hier:

<https://www.bfdi.bund.de/DE/Infothek/Anschriften_Links/anschriften_links-node.html>

### g) Dauer der Speicherung der Daten:

Die erhobenen Daten werden vom zentralen Studienzentrum / Sponsor für die Dauer von 10 Jahren nach Beendigung oder Abbruch der klinischen Studie gespeichert.

### h) Veröffentlichung

Wissenschaftliche Veröffentlichungen von Ergebnissen (auch open data) erfolgen in einer Form, die keine direkten Rückschlüsse auf Ihre Person zulässt. Alle personenbezogenen Informationen (wie z.B. Alter, Geschlecht, Pseudonym etc.) werden nicht veröffentlicht.

**Prüfstelle:** Köln-Bonner Akademie für Verhaltenstherapie (KBAV), Wenzelgasse 35

53111 Bonn, Dr. phil. Lisa Miebach, ************

**Zentrales Studienzentrum:** Professur für Psychosomatik und Psychotherapie, Psychologische Hochschule Berlin (PHB), Am Köllnischen Park 2, 10179 Berlin, a.gumz@phb.de

**Prüfer:** Prof. Dr. Antje Gumz

**Sponsor der klinischen Studie:** Psychologische Hochschule Berlin (PHB), Am Köllnischen Park 2, 10179 Berlin

DRKS number: DRKS00014842

**Einwilligungserklärung**

**Zur Teilnahme als Patient/in an der wissenschaftlichen Studie ,,Randomisiert kontrollierte Multicenter-Studie zur Therapieausbildung“.**Projektnummer 504346851

Ich hatte die Gelegenheit, für ein persönliches Gespräch das Studienteam in Berlin oder den Ansprechpartner, die Ansprechpartnerin für die Studie an meinem Institut zu kontaktieren.

Ich bin in einem persönlichen Gespräch ausführlich und verständlich über Wesen, Bedeutung, Risiken und Tragweite der klinischen Studie aufgeklärt worden. Ich hatte die Gelegenheit, über die Durchführung der klinischen Studie zu sprechen. Möglicherweise offene Fragen konnte ich in diesem Rahmen zufriedenstellend klären.

Ich habe darüber hinaus den Text der Studieninformation mit seinen beiden Teilen (Teil I: Informationen zum Studienablauf; Teil II: Informationen zur Verwendung der Daten) gelesen und verstanden.

Ich hatte ausreichend Zeit, mich zu entscheiden.

Mir ist bekannt, dass ich jederzeit und ohne Angabe von Gründen meine Einwilligung zur Teilnahme an der Studie zurückziehen kann (mündlich oder schriftlich), ohne dass mir daraus Nachteile entstehen.

**Datenschutzrechtliche Einwilligung**

Mir ist bekannt, dass bei dieser klinischen Studie **persönliche Informationen** über mich erhoben, gespeichert und ausgewertet werden sollen. Die Verwendung meiner personenbezogenen Daten setzt vor der Teilnahme an der klinischen Studie folgende freiwillig abgegebene Einwilligungserklärung voraus; ohne die nachfolgende Einwilligung kann ich nicht an der klinischen Studie teilnehmen.

1. Ich willige ein, dass im Rahmen dieser klinischen Studie persönliche Informationen, Audiodaten und Videodaten (Aufzeichnungen von Therapiesitzungen) über mich erhoben und in Papierform sowie auf elektronischen Datenträgern gemäß den Angaben in der Informationsschrift Teil II 1. aufgezeichnet, verwendet und weitergegeben werden.

2. Ich willige ein, dass meine Psychotherapeutin bzw. mein Psychotherapeut im Verlauf der Studie Basisinformationen zu meiner Therapie mittels Fragebogen an das zentrale Studienzentrum der Psychologischen Hochschule Berlin weitergeben darf, soweit dies für die ordnungsgemäße Durchführung und Überwachung der Studie notwendig ist. Insoweit entbinde ich diese Psychotherapeutin bzw. diesen Psychotherapeuten von der Schweigepflicht. Zu diesen Basisinformationen gehören: Ausfall von Therapiesitzungen, Abweichung der Dauer der Therapiesitzungen, Therapieabbrüche, Verstärken von Symptomen oder Auftreten neuer Symptome, Einschätzung der therapeutischen Beziehung und unerwünschte Ereignisse. Diese Basisinformationen werden ausschließlich pseudonymisiert, d.h. mit einem Studiencode, jedoch ohne identifizierende Informationen (wie Name, Geburtsdatum etc.) übermittelt. Es werden keine sonstigen Notizen oder Protokolle der Therapeutin bzw. des Therapeuten an das Studienteam übermittelt.

**Ich willige freiwillig ein, an der oben genannten klinischen Studie teilzunehmen.**

**Zugleich willige ich in die Verarbeitung meiner personenbezogenen Daten wie beschrieben und von mir angegeben ein.**

**Ich bin über Bedeutung der Videoaufzeichnung aufgeklärt worden. Ich erkläre mich einverstanden, dass im Rahmen des Projektes Videoaufnahmen der Therapiesitzungen angefertigt und zu Supervisions- und Studienzwecken benutzt wird.**

Ein Exemplar der Studieninformation und -einwilligung sowie die Versicherungsunterlagen habe ich erhalten. Ein Exemplar verbleibt im zentralen Studienzentrum

...........................................................................................................................

Name des **Patienten**, **der Patientin** in Druckbuchstaben

........................................

geb. am

.................................... ..............................................................................................

Ort/Datum Unterschrift des **Patienten**, **der Patientin**

Ich habe das Aufklärungsgespräch geführt und die Einwilligung des Patienten eingeholt.

...........................................................................................................................

Name **der aufklärenden Mitarbeiterin, des Mitarbeiters** am behandelnden Institut in Druckbuchstaben

.................................... ...............................................................................

Ort/Datum Unterschrift der **aufklärenden Mitarbeiterin, des Mitarbeiters**

**Einverständniserklärung zur Kontaktaufnahme**

bei studienbezogenen Anliegen

Sehr geehrte Patientin, sehr geehrter Patient,

während der Studie geben Sie regelmäßig Auskunft über den Verlauf Ihrer Symptome und über den Verlauf Ihrer Therapie in Form von Fragebögen und durch die Teilnahme an Telefoninterviews. Um eine Aussage darüber treffen zu können, wie sich die Symptome im Verlauf entwickeln und ob erhoffte Verbesserungen auch über einen längeren Zeitraum nach der Therapie anhalten, wird im Rahmen der Studie eine Erhebung nicht nur vor Therapiebeginn, sondern auch nach 20 Wochen, 35 Wochen, 20 Monate und 36 Monate nach der Randomisierung in die Kontroll- oder Interventionsgruppe durchgeführt.

Zur Durchführung der Telefoninterviews muss das Studienteam Sie kontaktieren, um einen Termin für ein Telefoninterview zu vereinbaren. Das Studienteam kontaktiert Sie für die Terminvereinbarung nur auf den Wegen, die Sie auf diesem Formular als Möglichkeit angeboten haben.

Zur Durchführung des Telefoninterviews benötigen wir von Ihnen zwingend mindestens eine Telefonnummer, unter der wir Sie erreichen können, um das Telefoninterview mit Ihnen durchzuführen.

An die Fragebogenerhebungen würden wir Sie mit Ihrer Einwilligung gern jeweils zwei Wochen vorher erinnern. Diese Fragebogenerhebungen finden unabhängig von Ihren Therapiesitzungen auch nach dem Ende Ihrer Psychotherapie statt und Sie können sie an einem Ort Ihrer Wahl durchführen.

Wenn Sie die Fragebogenerhebungen online am Tablet oder PC durchführen könnten, benötigen wir von Ihnen zwingend eine Emailadresse, um Ihnen jeweils für jede neue Erhebung einen persönlichen Link zusenden zu können. Wenn Sie die Fragebogenerhebungen auf Papier durchführen möchten, benötigen wir zwingend Ihre Postanschrift.

Mit diesem Schreiben möchten wir Sie um Ihr **Einverständnis a) zur erneuten Kontaktaufnahme, b) zur Durchführung der Telefoninterviews, c) zur Erinnerung an die Fragebogenerhebungen und um die entsprechend benötigten Kontaktinformationen** bitten. Dieses Einverständnis verpflichtet Sie nicht zur Teilnahme an der Erhebung. Das Einverständnis kann jederzeit ohne Begründung zurückgezogen werden.

**Hiermit erkläre ich mich damit einverstanden, dass ein Mitarbeiter, eine Mitarbeiterin der Psychologischen Hochschule Berlin mich zur Terminvereinbarung zur Durchführung der Telefoninterviews per E-Mail oder Telefon und für die Durchführung der Telefoninterviews telefonisch kontaktiert (Für eine Studienteilnahme ist diese Einwilligung zwingend.)**

ο **ja** ο **nein**

**Bitte geben Sie mindestens eine Telefonnummer an**.

............................................................ ........................................................

Telefonnummer 1 Telefonnummer 2

Bitte geben Sie Zeiten an, zu denen Sie bevorzugt zu erreichen sind:

............................................................

............................................................

............................................................

............................................................

Ich bevorzuge eine Terminabsprache auf folgendem Weg:

ο E-Mail ο SMS ο telefonisch

............................................................

E-Mail-Adresse

**Hiermit erkläre ich mich damit einverstanden, dass ein Mitarbeiter, eine Mitarbeiterin der Psychologischen Hochschule Berlin mir entweder den Link zur Durchführung von Fragebogenerhebungen per E-Mail zusendet (bei Online Bearbeitung der Fragebögen) oder mir die Fragebögen in Papierform an meine Adresse gesandt werden. (Für eine Studienteilnahme ist diese Einwilligung zwingend.)**

ο **ja** ο **nein**

**Bitte geben Sie mindestens eine E-Mail-Adresse an, wenn Sie die Fragebögen online ausfüllen möchten**.

............................................................

E-Mail-Adresse 1

**Bitte geben Sie Ihre Adresse an, wenn Sie die Fragebögen in Papierform bearbeiten möchten**.

............................................................

............................................................

............................................................

Postanschrift

**Hiermit erkläre ich mich damit einverstanden, dass ein Mitarbeiter, eine Mitarbeiterin der Psychologischen Hochschule Berlin mich per E-Mail oder Telefon kontaktiert, um mich an die Fragebogenerhebungen zu erinnern (Für eine Studienteilnahme ist diese Einwilligung NICHT zwingend.)**

ο **ja** ο **nein**

**Bei abweichenden oder noch fehlenden Kontaktinformationen geben Sie ggf. eine Emailadresse und/oder Telefonnummer dafür an.**

............................................................

E-Mail-Adresse 1

............................................................

Telefonnummer 1

Ich bevorzuge eine Erinnerung oder Terminabsprache auf folgendem Weg:

ο E-Mail ο SMS ο telefonisch

**Hiermit erkläre ich mich damit einverstanden, dass ein Mitarbeiter, eine Mitarbeiterin der Psychologischen Hochschule Berlin mich während der Studienlaufzeit aus anderen studienbezogenen Gründen (z.B. organisatorischen Problemen) kontaktieren kann.**

ο **ja** ο **nein**

....................................................................................
Datum, Unterschrift des **Probanden/der Probandin**

**Auszahlung der Aufwandsentschädigung**

Ich bitte um Auszahlung der Aufwandsentschädigung für die Teilnahme an der Studie auf folgendes Konto:

...........................................................................................................................

**Name und Vorname** der Kontoinhaberin/des Kontoinhabers

...........................................................................................................................

IBAN

...........................................................................................................................

BIC
