# Supplementary material for: Modified Alliance-Focused Training with Doubling as an integrative approach to improve therapists’ competencies in dealing with alliance ruptures and prevent negative outcomes in psychotherapy for depression: study protocol of a randomised controlled multicentre trial
Source: BMJ Open. 2025 Jul 16;15(7):e098343. doi: 10.1136/bmjopen-2024-098343 (PMC12273124; doi:10.1136/bmjopen-2024-098343)
Supplement: online supplemental file 2 [file bmjopen-15-7-s002.docx]

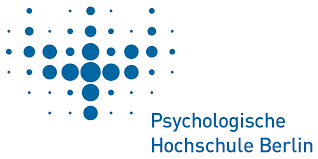
Prof. Dr. Antje Gumz

Professur für Psychosomatik und Psychotherapie

Psychologische Hochschule Berlin (PHB)

Am Köllnischen Park 2

10179 Berlin

**Prüfstelle:** Köln-Bonner Akademie für Verhaltenstherapie (KBAV), Wenzelgasse 35

53111 Bonn, Dr. phil. Lisa Miebach, ************

**Zentrales Studienzentrum:** Professur für Psychosomatik und Psychotherapie, Psychologische Hochschule Berlin (PHB), Am Köllnischen Park 2, 10179 Berlin, a.gumz@phb.de

**Prüfer:** Prof. Dr. Antje Gumz

**Sponsor der klinischen Studie:** Psychologische Hochschule Berlin (PHB), Am Köllnischen Park 2, 10179 Berlin

DRKS number: DRKS00014842

**Studieninformation**

**Randomisiert kontrollierte Multicenter-Studie zur Therapieausbildung**
Projektnummer 504346851

Sehr geehrte Therapeutin, sehr geehrter Therapeut,

wir möchten Sie fragen, ob Sie bereit sind, an der nachfolgend beschriebenen klinischen Studie teilzunehmen.

Die klinische Studie, die wir Ihnen hier vorstellen, wurde gemäß der berufsrechtlichen Vorgabe (§15 der Berufsordnung für nordrheinische Ärztinnen und Ärzte) beraten, und erhebt keine berufsrechtlichen oder berufsethischen Bedenken und stimmt somit der Durchführung der Studie zu.

Diese klinische Studie wird an mehreren Orten und verschiedenen Psychotherapieausbildungsinstituten durchgeführt; es sollen insgesamt ungefähr 240 Patienten und 120 Therapeuten daran teilnehmen. Die Studie wird durch den oben genannten Sponsor veranlasst und finanziert.

**Ihre Teilnahme an dieser klinischen Studie ist freiwillig.** Sie werden in diese Studie also nur dann einbezogen, wenn Sie dazu schriftlich Ihre Einwilligung erklären. Sofern Sie nicht an der klinischen Studie teilnehmen oder später aus ihr ausscheiden möchten, erwachsen Ihnen daraus keine Nachteile.

Der nachfolgende Text soll Ihnen die Ziele und den Ablauf erläutern. Der Text ist in drei Abschnitte gegliedert:

- Kurzdarstellung der Studie.
- Teil I: Informationen zum Studienablauf
- Teil II: spezifische Informationen zum Datenschutz

Neben dieser schriftlichen Information hatten Sie bereits oder haben Sie die Möglichkeit an einer Informationsveranstaltung zur Studie teilzunehmen sowie zu einem persönlichen Gespräch mit dem Studienteam in Berlin oder dem Ansprechpartner, der Ansprechpartnerin für die Studie an Ihrem Institut. Bitte zögern Sie nicht, alle Punkte anzusprechen, die Ihnen unklar sind. Sie können gerne Sätze/Abschnitte markieren, die Sie nicht verstanden haben, um sie zu besprechen. Nehmen Sie sich ausreichend Bedenkzeit, um über Ihre Teilnahme zu entscheiden.

**Kurzdarstellung der Studie**

**Grund für die Studie**: Ein zu hoher Anteil an Patienten mit Depressionen profitiert nicht ausreichend von Psychotherapie. Um die Ergebnisse von Psychotherapie zu verbessern, ist es entscheidend, die Faktoren, die zum Erfolg beitragen, zu kennen und an ihnen anzusetzen. Ausgehend von bereits bekannten Einflussfaktoren, möchten wir in der Studie untersuchen, wie sich Veränderung in Psychotherapieprozessen ereignet, welches therapeutische Vorgehen und welche Patienten- und Therapeuteneigenschaften dazu beitragen. Wir prüfen, wie sich hilfreiche von weniger hilfreichen Sitzungen unterscheiden und betrachten dabei Merkmale der therapeutischen Beziehungsgestaltung, Eigenschaften des Therapeuten bzw. der Therapeutin und des Patienten bzw. der Patientin, angewandte Techniken sowie sprachliche und nonverbale Merkmale (z.B. Stimme oder Bewegungsverhalten). Zudem prüfen wir einen neuen Trainings- und Supervisionsansatz für Therapeuten, der einen spezifischen Fokus auf einen der bekanntesten Einflussfaktoren für Therapieerfolg, die therapeutische Beziehung, legt. Dieser neue Ansatz soll mit der regulären Therapieausbildung verglichen werden. In die Studie werden Patienten mit Depressionen, Therapeuten in Ausbildung und deren Supervisoren eingeschlossen. Mit den Ergebnissen dieser Studie erhoffen wir uns, einen Beitrag zur Verbesserung der Qualität der Therapieausbildung und der ambulanten Depressionsbehandlung zu leisten.

**Studienablauf:** An der klinischen Studie nehmen Patienten mit der Diagnose einer depressiven Störung, Therapeuten in Ausbildung und Supervisoren teil. Die Studientherapien und -supervisionen sind Teil Ihrer Routineausbildung. Die Supervision im Rahmen der Studie wird in Form von Gruppensupervision (3 bis 4 Therapeuten je Gruppe) angeboten und entspricht den für die Psychotherapieausbildung vorgegebenen Rahmenbedingungen in Bezug auf Frequenz und Dosis. Studienpatienten sind erwachsene Patienten mit einer depressiven Störung, die eine ambulante Psychotherapie (Verhaltenstherapie oder tiefenpsychologisch fundierte Therapie) beginnen möchten.

Bei Eignung für die Studie findet das Baseline-Assessment statt (online; Fragebögen und eine Video-Übung zu herausfordernden Therapiesitutionen). Anschließend werden Sie zufällig einer der beiden Studiengruppen (reguläre Therapieausbildung vs. neuer Trainings- und Supervisionsansatz) zugeordnet. Studientherapeuten, die der Interventionsgruppe (neuer Trainings- und Supervisionsansatz) randomisiert zugeordnet wurden, nehmen anschließend an einem zweitägigen Workshop teil (Schulung im neuen Trainings- und Supervisionsansatz). In der Folge werden Ihnen entsprechend dem üblichen Prozedere an Ihrem Ausbildungsinstitut zwei Studienpatienten und eine Supervisionsgruppe, die jeweils derselben Studiengruppe (Interventions- oder Kontrollgruppe) angehören, zugeteilt. Die Aufnahme der beiden Studienpatienten kann zeitgleich oder im Abstand von wenigen Monaten geschehen. Der Abstand zwischen der Aufnahme der beiden Patienten hängt von der Rekrutierung der Patienten sowie von institutsspezifischen Regelungen ab.

Die Therapiesitzungen finden, wie üblich, wöchentlich für 50 Minuten statt. Die Dauer der Therapie (Kurzzeit- oder Langzeittherapie) legen Sie in Absprache mit Ihrer Supervisorin bzw. Ihrem Supervisor und der Patientin bzw. dem Patienten gemäß üblichem Vorgehen fest. Alle Therapiesitzungen werden videoaufgezeichnet. Nach jeder geplanten und durchgeführten Therapiesitzung füllen Sie einen Kurzfragebogen aus. Einmalig nach der 5. Woche enthält der Fragebogen zusätzliche Items. Die Kurzfragebögen können je nach Ihrer vorab angegebenen Präferenz mit PC, Tablet oder auf Papier ausgefüllt werden. Nach 20 Wochen, 35 Wochen, 20 Monaten und 36 Monaten nach der Randomisierung Ihres ersten Studienpatienten, Ihrer ersten -patientin findet eine weitere Erhebung mit Fragebögen statt (online), in der 35. Woche zusätzlich die Video-Übung (ebenfalls online). Die genannten studienbedingten Maßnahmen erfordern einen zusätzlichen Zeitaufwand von (ca. 4-7 Minuten) bei jedem Behandlungstermin.

Auch bei Ihren beiden Studienpatienten werden nach jeder Therapiesitzung Fragebogendaten erhoben und es finden innerhalb von 36 Monaten nach der ersten Erhebung fünf ausführlichere Erhebungen (mittels Fragebögen und z.T. Telefoninterviews) statt.

**Möglicher Nutzen für Sie:** Viele Therapeuten finden es interessant, mit Hilfe von Fragebögen über die eigenen Muster der Beziehungsgestaltung, über sich selbst und über die Therapie und die erzielten Veränderungen nachzudenken. In jedem Falle können die Fragebögen und die Video-Übung zu herausfordernden Therapiesitutionen dabei helfen, die eigene Wahrnehmung zu schärfen und bestimmte Punkte, die mit einer erfolgreichen Therapie in Zusammenhang stehen können, kontinuierlich unter die Lupe zu nehmen. Auch die an der Studie teilnehmenden Patienten füllen Fragebögen zu ihren Symptomen, zur Therapiebeziehung und zur Therapie über den Therapieverlauf hinweg aus. Dies kann die Therapie insgesamt unterstützen und intensivieren. Nichtsdestotzotz wissen wir natürlich, dass der damit einhergehende Aufwand auch als zusätzliche Belastung wahrgenommen werden kann.Für Ihren Aufwand erhalten Sie daher (zusätzlich zur gewohnten Vergütung für die Durchführung der Therapien) bis zu 1.785€ bei Verhaltenstherapien (bei 80 Sitzungen) und bis zu 1904€ bei tiefenpsychologisch fundierten Therapien (bei 100 Sitzungen; jeweils inkl. MwSt.). Details zur Aufwandsentschädigung finden Sie unter I. 7. Darüber hinaus bekommen Sie die Möglichkeit, den neuen Trainings- und Supervisionsansatz kostenlos kennenzulernen (entweder unmittelbar, falls Sie der Interventionsgruppe randomisiert zugeordnet werden oder im Anschluss an die Studie, falls Sie der Kontrollgruppe zugeordnet werden). Die Teilnahme an dem Workshop wird Ihnen für Ihre Ausbildung anerkannt (abhängig von der Absprache mit Ihrem Institut entweder als Teil der Theorieausbildung oder für die freie Spitze). Wenn Sie erst nach Studienabschluss teilnehmen können und bereits approbiert sein sollten, können Sie Fortbildungspunkte erhalten.

Mit Ihrer Studienteilnahme unterstützen Sie Psychotherapie- und Ausbildungsforschung, die langfristig möglicherweise dazu beiträgt, die ambulante Depressionsbehandlung zu verbessern.

**Risiken und Belastungen:** Die studienbedingten Erhebungen und die Datenerhebung können als zeitaufwändig empfunden werden. Das Nachdenken mit Hilfe von Fragebögen über die eigenen Muster der Beziehungsgestaltung, über sich selbst, die Therapie und die erzielten Veränderungen kann auch ein wenig anstrengend oder emotional aufwühlend erlebt werden. Wir erwarten keine weiteren Risiken oder unerwünschten Ereignisse aufgrund der Studienteilnahme.

**Freiwilligkeit:** Es ist Ihre freie Entscheidung, ob Sie an dieser Studie teilnehmen möchten oder nicht. Sie werden nur dann einbezogen, wenn Sie dazu schriftlich Ihre Einwilligung erkläre. Sie können jederzeit, auch ohne Angabe von Gründen, Ihre Einwilligung mündlich oder schriftlich widerrufen.

Zusätzlich zur schriftlichen Information werden Sie im Rahmen von Informationsveranstaltungen mündlich aufgeklärt. Bei offen Fragen oder Schwierigkeiten können sie sich vor und während Studienteilnahme jederzeit an das Studienteam (unter [studie@phb.de](mailto:studie@phb.de)), die Studienleiterin, Prof. A. Gumz ([a.gumz@phb.de](mailto:a.gumz@phb.de)) oder die Studienverantwortlichen an Ihrem Institut (Liste mit Namen und Kontaktdaten beiliegend) wenden. Wenn Sie sich für die Teilnahme entscheiden, füllen Sie bitte die Einwilligungserklärung aus.

# **Teil I: Informationen zum Ablauf der klinischen Studie**

**I. 1. Warum wird diese Studie durchgeführt?**

Depressive Störungen gehören zu den häufigsten Erkrankungen. Der Verlauf der Erkrankung ist häufig wiederkehrend oder chronisch und die Folgen für den Einzelnen und die Gesellschaft können schwerwiegend sein. Verhaltenstherapien und tiefenpsychologisch fundierte Psychotherapien sind wissenschaftlich geprüfte, wirksame Therapieverfahren.

Trotz der generellen guten Wirksamkeit dieser Therapieverfahren, gibt es viele depressive Patienten, die nicht ausreichend von ihrer Psychotherapie profitieren und viele Patienten brechen ihre Psychotherapie vorzeitig ab. Damit noch mehr Patienten von einer Psychotherapie profitieren, ist es wichtig zu untersuchen, welche Faktoren zum Therapieerfolg beitragen. Ob eine Therapie wirksam ist, hängt von verschiedenen therapieprozessbezogenen, patientenseitigen und therapeutenseitigen Faktoren ab. Ein bekanntermaßen entscheidender Faktor ist, ob es gelingt, eine hilfreiche und vertrauensvolle Therapiebeziehung herzustellen und aufrechtzuerhalten.

Wir möchten in unserer Studie daher auch untersuchen, wie ein neuer Trainings- und Supervisionsansatz mit einem spezifischen Fokus auf der Herstellung oder Aufrechterhaltung einer guten therapeutischen Beziehung im Vergleich zur regulären Psychotherapieausbildung wirkt. Gleichzeitig erforschen wir, wie sich therapeutische Veränderung in den Therapieprozessen konkret ereignet, welches therapeutische Vorgehen und welche Patienten- und Therapeutenmerkmale mit besserem Therapieerfolg einhergehen. Wir prüfen, wie sich hilfreiche Sitzungen von weniger hilfreichen Sitzungen unterscheiden. Dabei betrachten wir Merkmale der therapeutischen Beziehungsgestaltung, Eigenschaften und angewandte Techniken der Therapeuten sowie sprachliche und nonverbale Merkmale (z.B. Stimme oder Bewegungsverhalten).

Von der Durchführung der vorgesehenen Studie erhoffen wir uns, einen Beitrag zur Qualität der Psychotherapieausbildung und hierüber zur Verbesserung der ambulanten Depressionsbehandlung zu leisten.

**I. 2. Wie läuft die Randomisierung ab?**

Im Rahmen dieser klinischen Studie wird ein neuer Trainings- und Supervisionsansatz mit einem spezifischen Fokus auf der therapeutischen Beziehung mit regulärer Psychotherapieausbildung verglichen. Zu diesem Zwecke findet eine Randomisierung statt. Sowohl die Studientherapeuten, als auch die Studienpatienten werden randomisiert. Das bedeutet, dass Sie nach einem Zufallsverfahren einer von zwei Studiengruppen zugeordnet werden: In der Studiengruppe 1 befinden sich die Patienten, die eine Therapie bei Therapeuten bekommen, deren Ausbildung einen neuen Ausbildungs- und Supervisionsansatz enthält, in Gruppe 2 befinden sich Patienten, die eine Therapie bei Therapeuten bekommen, deren Ausbildung wie üblich erfolgt. Die Randomisierung wird in Blöcken von 6-8 Therapeuten je Institut durchgeführt, d.h. an Ihrem Institut müssen 6 bis 8 Therapeuten in die Studienteilnahme eingewilligt haben, damit die Randomisierung anschließend stattfinden kann. Sobald Sie randomisiert wurden, werden Sie über das Ergebnis, d.h. Ihre Zuordnung, informiert.

Die Patienten erhalten keine Information über die Zuordnung, d.h. sie bleiben verblindet. Zur objektiven Gewinnung von Studiendaten ist es notwendig, dass die Ihnen zugeordneten Studienpatienten keinesfalls erfahren, in welcher Studienbedingung Sie sich befinden („**Verblindung**“).

Sie erhalten Supervision, bei einer Supervisorin bzw. einem Supervisor der gleichen Studienbedingung, d.h., Supervisoren der Interventionsgruppe (Studienbedingung 1) werden ebenfalls in dem neuen Trainings- und Supervisionsansatz geschult, Supervisoren der Kontrollgruppe supervidieren gemäß üblichem Vorgehen.

Therapeuten, die in die Studienbedingung 1 randomisiert werden, erhalten unmittelbar nach der Randomisierung eine zusätzliche Studieninformation, in der die Intervention (der Trainings- und Supervisionsansatz) beschrieben wird und die Hinweise zum Umgang mit der „Verblindung“ enthält.

**I. 3. Wie ist der Ablauf der Studie und was muss ich bei Teilnahme beachten?**

An der klinischen Studie nehmen Patienten mit der Diagnose einer depressiven Störung, Therapeuten in Ausbildung und Supervisoren teil. Die Studientherapien sind Teil Ihrer Routineausbildung, d.h. sie werden als Ausbildungsfälle anerkannt.

Therapeuten können teilnehmen, wenn sie sich in Ausbildung in tiefenpsychologisch fundierter Psychotherapie oder Verhaltenstherapie für Erwachsene (Dipl.-Psych., M. Sc. Psych. oder ein Staatsexamen Medizin) befinden und die Behandlungserlaubnis erhalten haben. Zudem müssen Sie die zeitliche Kapazität haben, zwei Patienten im Rahmen der Studie aufzunehmen, an einem der angebotenen Workshoptermine teilzunehmen (Schulung im neuen Trainings- und Supervisionsansatz) und die von den Studiensupervisoren vorgegebenen Supervisionszeiten einzurichten.

Die Supervision im Rahmen der Studie wird in Form von Gruppensupervision (3 bis 4 Therapeuten je Gruppe) angeboten und entspricht den für die Psychotherapieausbildung vorgegebenen Rahmenbedingungen in Bezug auf Frequenz und Dosis. Die Rahmenbedingungen wurden mit den Verantwortlichen an Ihrem Insittut abgestimmt und sind identisch für beide Studiengruppen. Je nach Absprache mit dem jeweiligen Institut und den Supervisoren finden die Gruppensupervisionen monatlich, zwei-wöchentlich oder seltener wöchentlich statt. Die Dauer ist so kalkuliert, dass jeder Patient, jede Patientin bzw. jeder Therapieverlauf durchschnittlich 20 bis 25 Minuten pro Monat besprochen werden kann. Die Finanzierung der Gruppensupervision entspricht den üblichen Bedingungen (je nach Institut entweder individuelle Abrechnung zwischen Supervisor/in und Therapeut/in oder Abrechnung zwischen Supervisor/in und Institut bei inkludierten Supervisionskosten).

Studienpatienten sind erwachsene Patienten mit einer depressiven Störung, die eine ambulante Psychotherapie (Verhaltenstherapie oder tiefenpsychologisch fundierte Therapie) beginnen möchten. Das Studienteam prüft die Ein- und Ausschlusskriterien für eine Studienteilnahme bei den Patienten.

Innerhalb einer Zeitspanne von 36 Monaten finden bei den Therapeuten insgesamt fünf Online-Erhebungen statt (Fragebögen und bei zwei Messzeitpunkten zusätzlich eine Video-Übung zu herausfordernden Therapiesitutionen.) Zudem wird nach jeder Therapiesitzung ein Kurzfragebogen (elektronisch) ausgefüllt, in der jeweils 5. Woche nach Randomisierung des Patienten erhält der Fragebogen einmalig einige zusätzliche Items.

Bei den Patienten findet innerhalb der Zeitspanne insgesamt fünf Mal eine ausführlichere Erhebung (mit Fragebögen und Telefoninterviews) statt. Zusätzlich werden auch bei den Patienten nach jeder Therapiesitzung Fragebogendaten erhoben (ca. 6-8 Minuten). Auch die Patienten erhalten in der 5. Woche einen weiteren Fragebogen.

Der Ablauf der Studie erfolgt für Sie in folgenden Schritten:

1. Zunächst prüft das Studienteam, ob Sie die Einschlusskriterien für die Studie erfüllen (anhand eines Kurzfragebogens, der dieser Studieninformation und der Einverständniserklärung beiliegt).
2. Bei Eignung findet die Baseline-Erhebung statt (online). Den genauen Zeitpunkt stimmen wir mit Ihnen ab, da zwischen dieser Erhebung und Ihrer Aufnahme von Studienpatienten nicht allzu viel Zeit vergehen soll. Die Baselinemessung umfasst Fragebögen und eine Video-Übung zu herausfordernden Therapiesitutionen. Bitte planen Sie für die Fragebögen (ca. 60-90 min) und die Video-Übung (ca. 20-30 min.) Zeit an zwei separaten Tagen ein. Beide Erhebungen finden online statt. Während der Beantwortung der Fragebögen und der Video-Übung sollen Sie sich ungestört und unbeobachtet in einem ruhigen Raum befinden. Wenn Sie die Video-Übung durchführen, richten Sie sich bitte selbst im Vorfeld bestmöglich darauf ein, da die Video-Übung zwischendurch nicht unterbrochen werden kann. In der Video-Übung werden Sie 6 herausfordernde Therapiesituationen in kurzen Videoclips sehen. Sie werden gebeten, sich in die Rolle der Therapeutin bzw. des Therapeuten zu versetzen und auf die Clips spontan zu reagieren. Ihre spontanen Antworten an die dargestellten Patienten werden audioaufgezeichnet. Anschließend bewerten Sie Ihre Reaktionen in der Video-Übung selbst mit Hilfe eines kurzen Selbstbeurteilungsbogens. (Die Zugänge zu den Fragebögen und der Video-Übung erhalten Sie per Email. Bitte beachten Sie, dass wir im Laufe der Studie für die Erhebungen **zwei verschiedene Email-Adressen** von Ihnen benötigen werden.)
3. Anschließend findet die Randomisierung statt. Sie werden einer der beiden Studiengruppen zufällig zugeordnet. Nach der Zuordnung werden Sie über das Ergebnis sowie die damit verbundene Supervisionsgruppe (d.h. Supervisor/in, mögliche Zeiten) informiert.
4. Studientherapeuten, die der Interventionsgruppe randomisiert zugeordnet wurden, erhalten weitere Informationen zur Intervention und nehmen anschließend am zweitägigen Workshop teil (Schulung im neuen Trainings- und Supervisionsansatz).
5. Nach Ihrer Randomisierung und - bei Zuordnung zur Interventionsgruppe - der Teilnahme am Workshop, können Ihnen Studienpatienten zugeordnet werden. Wir bitten Sie, Ihre Kapazitäten sowie längere Abwesenheiten mit der/dem Studienverantwortlichen an Ihrem Institut abzusprechen. Die Patientenunterlagen erhalten Sie anschließend auf dem gewohnten Weg. Anschließend können Sie mit dem Patienten, der Patientin verabreden, wann die erste Therapiesitzung stattfinden kann. Die Therapiesitzungen finden, wie üblich, wöchentlich für 50 Minuten statt. Die Dauer der Therapie (Kurzzeit- oder Langzeittherapie) legen Sie in Absprache mit Ihrer Supervisorin bzw. Ihrem Supervisor und der Patientin, dem Patienten gemäß üblichem Vorgehen fest. Bitte beachten Sie, dass innerhalb der ersten 20 Wochen nach Aufnahme der Patientin, des Patienten **mindestens 14 Therapiesitzungen** stattfinden sollen. Wir bitten Sie daher in Absprache mit dem Supervisor bzw. der Supervisorin so gut wie möglich längere Therapieunterbrechungen zu vermeiden (z.B. frühe Beantragung, Beantragung der nicht-berichtspflichtigen KZT im ersten Schritt).
6. Alle Therapiesitzungen werden videoaufgezeichnet. Als Therapeutin bzw. als Therapeut werden Sie für die Videoaufzeichnung verantwortlich sein. Sollte die technische Ausstattung zur Videoaufzeichnung (Kamera, ggf. Stativ) an Ihrem Institut nicht zur Verfügung gestellt werden können, können andere aufnahmefähige Geräte (Handy oder Laptop) genutzt werden, allerdings ausschließlich unter folgenden Bedingungen: Die Geräte dürfen während der Aufnahme keine Verbindung zum Internet haben und die Verbindung zum Internet darf erst wiederhergestellt werden, wenn die Therapiesitzung beendet ist und vom Laptop bzw. Handy vollständig gelöscht wurde. Die aufgezeichneten Videos, die für Forschungszwecke verwendet werden, werden nach der Therapiesitzung durch Sie verschlüsselt über einen virtuellen privaten Tunnel (SSL-VPN) an das Rechenzentrum (RZ) der Psychologischen Hochschule Berlin (PHB) übertragen. Die Installation des VPN-Tunnels, sowie die Einweisung, wie die Übertragung/Verschlüsselung der Daten funktioniert, erfolgt durch das Studienpersonal der PHB. Sie werden außerdem eine leicht verständliche Handreichung dafür erhalten, die spezifisch auf die Bedingungen an Ihrem Institut zugeschnitten ist. Das Vorgehen dabei sowie institutsspezifische Regelungen (z.B. Kamera-Buchungskalender) werden in der Handreichung „Videoaufzeichnungen“ beschrieben. Die Videoaufzeichnungen können von Ihnen zudem in der Supervision genutzt werden. Die Videos, die Sie für die Supervision nutzen, werden von Ihnen an dem zentralen Computer Ihres Instituts mit Vera-Crypt verschlüsselt und anschließend auf externen Speicherträgern gespeichert. Somit können Sie einzelne Videos für die Supervision nutzen. Nach der Supervision werden diese einzelnen Sitzungen durch Sie von dem verschlüsselten Speicherträger vollständig gelöscht. Einige der von Ihnen an die PHB übertragenen Sitzungen aus den Therapieverläufen werden zusätzlich für Forschungszwecke genutzt. Anhand dieser Sitzungen werden wissenschaftliche Mitarbeiter unserer Forschungsgruppe, die der Verschwiegenheit verpflichtet sind, zum einen einschätzen, inwieweit Therapeuten der Interventionsgruppe ihr Vorgehen im Sinne des neuen Ansatzes ausgerichtet haben, d.h., inwieweit sie anders vorgehen als die Therapeuten der Kontrollgruppe. Dies dient der Einschätzung der Qualität der Intervention und der Studie. Außerdem möchten wir einzelne Sitzungen auf Basis der Fragebogenantworten von Ihnen und Ihrem Patienten, Ihrer Patientin auswählen und Merkmale der therapeutischen Beziehungsgestaltung, therapeutische Eigenschaften und Techniken sowie sprachliche und nonverbale Merkmale (z.B. Stimme oder Bewegungsverhalten) untersuchen. Diese Erhebungen dienen dazu, unsere Fragestellungen zu Einflussfaktoren des Therapieerfolgs zu beantworten. Je nach Usus am Ausbildungsinstitut können die Videoaufzeichnungen sowohl für Therapeuten als auch für Patienten zu Beginn ungewohnt sein. Erfahrungsgemäß setzt ein Gewöhnungseffekt recht schnell innerhalb der ersten Stunden ein.

Nach jeder Therapiesitzung füllen Sie einen Kurzfragebogen aus (4 bis 7 Minuten). Einmalig nach der 5. Woche enthält der Fragebogen zusätzliche Items und das Ausfüllen dauert ca. 5 Minuten länger. Die Fragebögen können am PC, mit Tablet oder auf Papier ausgefüllt werden.

7. Jeweils 20 Wochen, 35 Wochen, 20 Monate und 36 Monate nach der Randomisierung Ihres ersten Studienpatienten, der ersten Studienpatientin findet eine weitere Erhebung mit Fragebögen statt (30 bis 45 Minuten). In der 35. Woche findet zusätzlich die zweite Video-Übung zu herausfordernden Therapiesitutionen satt (zusätzlich 15 bis 20 Minuten). Mit Ihrer Einwilligung werden Sie an die Termine vorab erinnert. Diese Erhebungen finden unabhängig von den Therapiesitzungen statt und werden ausschließlich online angeboten. Sie können diese Erhebungen an einem Ort Ihrer Wahl durchführen. Wir bitten Sie sicherzustellen, dass eine ruhige Atmosphäre herrscht und Sie für die Dauer der Erhebungen ungestört sind.

Wichtig ist zu ergänzen: **Die Therapeuten erhalten ohne Ausnahme keinen Einblick in die Daten der Patienten und die Patienten erhalten keinen Einblick in die Daten der Therapeuten!**

**I. 4. Welchen persönlichen Nutzen habe ich von der Teilnahme an der Studie?**

Wir erhoffen uns aufgrund bisheriger Forschungsergebnisse, dass ein neuer Ausbildungs- und Supervisionsansatz im Vergleich zur regulären Psychotherapieausbildung die Therapieergebnisse der Patienten zusätzlich verbessern kann. Wenn Sie mit der neuen Methode ausgebildet werden, so kann dies möglicherweise dazu beitragen, dass Ihre Therapien erfolgreicher verlaufen. Da die Wirksamkeit der Methode noch nicht erwiesen ist, ist es jedoch auch möglich, dass Sie durch Ihre Teilnahme an dieser Stelle keinen erhofften Nutzen haben. Ihre Teilnahme an dem Workshop wird für Ihre Ausbildung anerkannt (je nach Absprache mit dem Institut als Bestandteil der Theorieausbildung oder für die freie Spitze). Sollten Sie erst nach Studienabschluss teilnehmen und bereits apporbiert sein, können Sie Fortbildungspunkte erhalten. Die Workshopteilnahme ist für Sie kostenlos.

Viele Therapeuten in unseren Vorstudien fanden es interessant, mit Hilfe von Fragebögen über die eigenen Muster der Beziehungsgestaltung, über sich selbst und über ihre Therapie und die erzielten Veränderungen nachzudenken. In jedem Falle können die Fragebögen und die Video-Übung zu herausfordernden Therapiesitutionen dabei helfen, die eigene Wahrnehmung zu schärfen und bestimmte Punkte, die mit einer erfolgreichen Therapie in Zusammenhang stehen können, kontinuierlich unter die Lupe zu nehmen. Auch die an der Studie teilnehmenden Patienten füllen Fragebögen zu ihren Symptomen, zur Therapiebeziehung und zur Therapie über den Therapieverlauf hinweg aus. Dies kann die Therapie insgesamt unterstützen und intensivieren.

Für Ihren Aufwand im Zusammenhang mit der Studie erhalten Sie, über die gewohnte Vergütung der durchgeführten Therapiesitzungen hinaus, eine Aufwandsentschädigung (s. I. 7).

Mit Ihrer Studienteilnahme unterstützen Sie Psychotherapie- und Ausbildungsforschung, die langfristig möglicherweise dazu beiträgt, die ambulante Depressionsbehandlung zu verbessern.

**I.** **5. Welche gesundheitlichen Risiken und Belastungen sind mit der Teilnahme an der Studie verbunden?**

Die studienbedingten Erhebungen und die Datenerhebung können als zeitaufwändig empfunden werden. Das Nachdenken mit Hilfe von Fragebögen über die eigenen Muster der Beziehungsgestaltung, über sich selbst, die Therapie und die erzielten Veränderungen kann auch ein wenig anstrengend oder emotional aufwühlend erlebt werden. Wir erwarten keine weiteren Risiken oder unerwünschten Ereignisse aufgrund der Studienteilnahme.

**I. 6. Wer darf an dieser klinischen Studie nicht teilnehmen?**

Sie können an dieser klinischen Studie nur teilnehmen, wenn Sie gesund sind und sich nicht gleichzeitig für andere klinische Studien oder Forschungsprojekte zur Verfügung stellen*.*

**I. 7. Entstehen für mich Kosten durch die Teilnahme an der klinischen Studie? Erhalte ich eine Aufwandsentschädigung?**

Durch Ihre Teilnahme an dieser klinischen Studie entstehen für Sie keine Kosten. Die Finanzierung der Supervision erfolgt entsprechend gewohnter Regelungen an Ihrem Ausbildunginstitut. Die Supervisionen gelten wie üblich als Bestandteil Ihrer Ausbildung. Die Therapeuten der Interventionsgruppe können kostenlos am Workshop teilnehmen und bekommen die Reise- und Übernachtungskosten erstattet, wenn der Workshop an einem Ort außerhalb des Wohnortes bzw. des Ortes, an dem sich das Ausbildungsinstitut befindet, stattfindet (Bahnfahrt 2. Klasse, zzgl. Kosten für eine Übernachtung in Höhe des für den öffentlichen Dienst üblichen Rahmens). Therapeuten der Kontrollgruppe erhalten bei Interesse den Workshop kostenlos nach dem Studienende. Auch der Workshop wird für Ihre Ausbildung anerkannt (für die Theorieausbildung oder die freie Spitze).

Für Ihre Teilnahme an dieser Studie erhalten Sie eine Aufwandsentschädigung (zusätzlich zu Ihrem üblichen Therapeutenhonorar für die durchgeführten Therapiesitzungen) entsprechend den folgenden Bedingungen:

- Therapeuten, die an der Studie mitwirken, erhalten bei bis zur 20. Woche nach Randomisierung des Patienten mind. 95% vollständigen Erhebungen pro Patient/in und bei einer bis max. 20 absolvierten Sitzungen: 595€ inkl. MwSt. Zusätzlich erhalten Sie (jeweils inkl. MwSt):
- bei 40 absolvierten Sitzungen und zu mind. 95% vollständigen Erhebungen: 119€
- bei 60 absolvierten Sitzungen und zu mind. 95% vollständigen Erhebungen: 119€
- bei 80 absolvierten Sitzungen und zu mind. 95% vollständigen Erhebungen: 60€
- bei 100 absolvierten Sitzungen und zu mind. 95% vollständigen Erhebungen: 59€

Dies entspricht einer maximalen Gesamtaufwandsentschädigung von 1904 € für TP Therapeuten TP und 1785 € für VT-Therapeuten bei jeweils zwei Studienpatienten (jeweils inkl. MwSt 19%). Bitte beachten Sie, dass Sie die Aufwandsentschädigung auch erhalten, wenn der Patient, die Patientin die Therapie früh abbrechen sollte (595€ bei einem Abbruch vor der 20. Sitzung und nahezu vollständigen Erhebungen). Sollten Sie und/oder Ihr Patient, Ihre Patientin sich jedoch für einen Therapeutenwechsel innerhalb der Probatorik entscheiden, wird Ihnen diese Summe nicht ausgezahlt. Stattdessen haben Sie jedoch die Möglichkeit, einen weiteren Studienpatienten, weitere Studienpatientin aufzunehmen.

**I. 9. Werden mir neue Erkenntnisse zu der klinischen Studie mitgeteilt?**

Sie werden während Ihrer Teilnahme über neue Erkenntnisse in Bezug auf diese Studie informiert, die für Ihre Bereitschaft zur weiteren Teilnahme wesentlich sein können.

# Nach Beendigung der gesamten Studie wird das Studienteam zusammenfassende Ergebnisse in der Datenbank des Deutschen Registers Klinischer Studien (<https://drks.de/search/de/results>) bereitstellen. Dies kann von Ihnen unter der oben angegebenen trial number eingesehen werden. Sie können sich nach Studienende zur Information über die Studienergebnisse zudem gerne an das Studienteam wenden.

**I. 10. Wer entscheidet, ob ich aus der klinischen Studie ausscheide?**

**Sie können jederzeit, auch ohne Angabe von Gründen, Ihre Teilnahme beenden, ohne dass Ihnen dadurch Nachteile entstehen.**

Es ist auch möglich, dass der Sponsor entscheidet, Ihre Teilnahme an der klinischen Studie vorzeitig zu beenden. Ein möglicher Grund dafür kann sein, dass die gesamte klinische Studie abgebrochen werden muss (z.B. falls ein Zusammenhang zwischen dem Vorkommen schwerwiegender unerwünschter Ereignisse und der Studienteilnahme festgestellt wird).

**I. 11. An wen wende ich mich bei weiteren Fragen?**

Bei offen Fragen oder Schwierigkeiten können sie sich vor und während Studientilnahme jederzeit an das zentrale Studienzentrum ([studie@phb.de](mailto:studie@phb.de)), die Studienleiterin Prof. A. Gumz ([a.gumz@phb.de](mailto:a.gumz@phb.de)) oder die Studienverantwortlichen an Ihrem Institut (Liste mit Namen und Kontaktdaten beiliegend) wenden.

# **Teil II: Informationen zum Datenschutz**

**II. 1. Was geschieht mit den über mich erhobenen Daten?**

### a) Allgemeine Informationen

Während der klinischen Studie werden persönliche Informationen von Ihnen erhoben und ggf. kurzfristig im lokalen Studienzentrum (d.h. in Ihrem Institut) in verschlossenen Umschlägen und dauerhaft beim zentralen Studienzentrum (an der Psychologischen Hochschule Berlin) gesichert gelagert (in verschlossenen Schränken). Abgesehen von der Einwilligungserklärung und der Dokumentaion Ihrer Anwesenheiten bei den Gruppensupervisionssitzungen und Workshops (diese werden von den Supervisions- und Workshopleitern notiert), die Ihren Namen und weitere persönliche Informationen enthalten, werden alle anderen Studiendaten nur in pseudonymisierter Form erhoben, verarbeitet und gespeichert.

Pseudonymisiert bedeutet, dass keine Angaben, mit denen Sie direkt identifiziert werden können (z.B. Namen, Kontaktinformationen, Geburtsdatum, etc.) verwendet werden, sondern nur ein Nummern- und Buchstabencode*.* Dieser Code, d.h. die Therapeuten-ID, besteht aus neun Zeichen und setzt sich folgendermaßen zusammen: a) 2 Ziffern, die Ihr Institut kennzeichnen; b) T (für Therapeut); c) eine fortlaufende dreistellige Ziffer beginnend mit 1001; d) die ersten zwei Buchstaben des Vornamens der Mutter, und e) der erste Buchstabe Ihres Geburtsortes. **Wir bitten Sie, die letzten drei Stellen (d und e) auf dem beiliegenden Kurzbogen dementsprechend zu vervollständigen.**

**Bitte notieren oder merken Sie sich Ihren Code.** Alle weiteren Studienunterlagen, die für Sie persönlich bestimmt sind, werden nur diesen Code enthalten. Zudem bitten wir Sie, die Videoaufzeichnungen Ihrer Therapiesitzungen in verschlüsselten Ordnern zu speichern, die nach Ihrem Studiencode benannt sind und zusätzlich eine Kennzeichnung für den jeweiligen Patienten, die Patientin enthalten.

Der Datenschlüssel, der eine Zuordnung der Studiencodes zu den tatsächlichen Therapeuten-

, Patienten- und Supervisorenidentitäten enthält, wird während der Studienlaufzeit und bis zu zehn Jahre nach Ende der Studienlaufzeit im zentralen Studienzentrum (Psychologische Hochschule Berlin, PHB) gesichert (d.h. elektronisch verschlüsselt oder im Fall von physischen Kopien in einem verschlossenen Schrank) aufbewahrt. Der Schlüssel wird jederzeit getrennt von allen anderen Studiendaten gelagert. Die schriftliche Kommunikation zwischen den lokalen Studienzentren (Ausbildungsinstituten) und dem zentralen Studienteam (PHB) erfolgt ausschließlich über die Studiencodes. In definierten Ausnahmefällen (bei anhaltenden Schwierigkeiten bei der Datenerhebung, bei organisatorischen Problemen) werden Namen in der Kommunikation zwischen lokalen Studienverantwortlichen und zentralem Studienteam verwendet. Dies geschieht jedoch ausschließlich über telefonische Kontakte und, um die Pseudonymisierung zu erhalten, ausnahmslos ohne Nennung von Studiencodes.

Zugang zu dem „Schlüssel“, der eine persönliche Zuordnung des Studienteilnehmers, der Studienteilnehmerin ermöglicht, haben nur von der Studienleiterin Prof. Dr. Antje Gumz ausdrücklich dazu autorisierte Projektmitarbeiter, die zum Kernstudienteam gehören und der Schweigepflicht unterliegen. Eine Entschlüsselung erfolgt nur, wenn das Studienteam aus spezifischen studienbezogenen Gründen Kontakt zu Ihnen aufnehmen muss (z.B. zur Erinnerung an studienbezogene Erhebung mit Ihrer Einwilligung, zur Zusendung Ihrer Zugänge zu den Befragungen, für eine Kurzbefragung bei Therapie- und Studienabbruch des Patienten, der Patientin mit der Einwilligung von Ihnen und des Patienten, der Patientin) sowie zur Übertragung der Anwesenheiten bei den Gruppensupervisionssitzungen und Workshops (diese Anwesenheiten werden von den Supervisions- und Workshopleitern namentlich dokumentiert). Der individuelle Datenschlüssel wird zehn Jahre nach Ende der Studienlaufzeit dauerhaft gelöscht. Die sichere Aufbewahrung des Schlüssels über diese Zeit hinweg ist nötig, damit das Studienteam sicherheitsrelevante Daten auch nach dem Ablauf der Studie noch zuordnen kann.

Trotz der Pseudonymisierung lässt sich niemals völlig ausschließen, dass auch ohne den Datenschlüssel Rückschlüsse auf Ihre Person gezogen werden können. Dies trifft insbesondere auf die von Ihnen im Rahmen der Video-Übung zu herausfordernden Therapiesituationen erhobenen Audiodaten sowie auf die Videoaufzeichnungen der Sitzungen zu.

Zum Schutz Ihrer im Rahmen der Video-Übung erhobenen Audiodaten werden diese nach Abschluss Ihrer Video-Übung mittels gängiger Verfahren stimmlich verfremdet. Mit der so vorgenommenen faktischen Anonymisierung der Audiodaten soll eine Zuordnung zu Ihrer Person ganz erheblich erschwert bis ausgeschlossen werden. Für die Kodierung Ihrer Audio-Antworten durch Studienmitarbeiter und linguistische Analysen werden wir ausschließlich mit den stimmlich verfremdeten Audiodaten arbeiten. Für phonetische Analysen, die ganz vorrangig automatisiert vorgenommen werden, verwenden wir die Originalaufnahmen. Die Originalaufnahmen werden nach Abschluss der phonetischen Analysen gelöscht.

Die originalen und verfremdeten Audioaufzeichnungen werden während der Studienlaufzeit in einem verschlossenen Schrank aufbewahrt und von der jeweiligen Studienmitarbeiterin in einem verschlüsselten Raidsystem gesichert. Die Audioaufzeichnungen werden nach abgeschlossener Auswertung vernichtet. Im Rahmen der Online-Teilnahme werden die Audiodaten zunächst kurzzeitig auf einem sicheren deutschen Server gespeichert. Die stimmliche Verfremdung wird automatisiert nach Aufnahme durchgeführt. Zur weiteren zuvor beschriebenen Aufbewahrung lädt das Studienteam die Aufnahmen in regelmäßigen kurzfristigen Abständen herunter und löscht diese vom Server.

Die videoaufgezeichneten Therapiesitzungen werden von Ihnen wöchentlich verschlüsselt über eine gesicherte Tunnelverbindung (VPN) per Internet an das Rechenzentrum (RZ) der Psychologischen Hochschule Berlin (PHB) verschickt und dort gespeichert. Die VPN-Verbindungen werden vom Studienteam vorbereitet, Sie erhalten ein individuelles Passwort. Sollte die technische Ausstattung zur Videoaufzeichnung (Kamera, ggf. Stativ) an Ihrem Institut nicht zur Verfügung gestellt werden können, können andere aufnahmefähige Geräte (Handy oder Laptop) genutzt werden, allerdings ausschließlich unter folgenden Bedingungen: Die Geräte dürfen während der Aufnahme keine Verbindung zum Internet haben und die Verbindung zum Internet darf erst wiederhergestellt werden, wenn die Therapiesitzung auf die gesicherten, verschlüsselten Datenträger überspielt wurde und auf dem Laptop bzw. Handy vollständig gelöscht wurde. Für die Verschlüsselung des Videos erhalten Sie ein individuelles Passwort. Zugriff auf die Videodaten im RZ der PHB hat nur Ihre Therapeutin bzw. Ihr Therapeut sowie von der Studienleiterin Prof. Dr. Antje Gumz ausdrücklich dazu autorisierte Studienmitarbeiter, die die der Schweigepflicht unterliegen. Weiterhin Mitarbeiter der IT der PHB, die ebenfalls der Schweigepflicht unterliegen. Einzelne Sitzungen aus den Therapieverläufen werden von diesen ausgewählten Studienmitarbeitern ausgewertet. Jedes zur Auswertung bestimmte Video wird von einem Studienmitarbeiter auf identifizierende Informationen (z.B. Nennung von Namen) geprüft und diese werden entfernt. Diese Entfernung von identifizierenden Informationen übernimmt derjenige Studienmitarbeiter, der bereits Zugang zu persönlichen Daten des Patienten, der Patientin im Rahmen der Durchführung von Telefoninterviews hatte, sofern dies möglich ist. Zur Auswertung der Videositzungen werden Ratinginstrumente, phonetische, linguistische und Bewegungsmuster-Analysen genutzt. Analog zum Vorgehen bei den Audiodaten wird für die jeweilige Auswertung die Datengrundlage genutzt, die Ihre und die Anoymität des Patienten, der Patientin am besten schützt. Beispielsweise werden für spezifische Auswertungen lediglich vollständig anonymisierte Transkripte der Videositzungen verwendet. Einige Auswertungen beruhen jedoch auch auf Mimik, Gestik und anderen non-verbalten Merkmalen, so dass das Video notwendig ist. Die Ergbnisse dieser Auswertungen werden ausschließlich pseudonymisiert gespeichert.

Die auf dem externen Speicherträger verschlüsselt gespeicherten Videoaufzeichnungen werden nach der Supervision vollständig gelöscht. Die im RZ der PHB verschlüsselt gespeicherten Videoaufzeichnungen werden während der Studienlaufzeit und bis maximal 10 Jahre nach Ende der Studienlaufzeit so wie der oben beschriebene Datenschlüssel in einem verschlossenen Schrank aufbewahrt und anschließend vollständig vernichtet.

### b) Rechtsgrundlage

Rechtsgrundlage für die Datenverarbeitung ist Ihre informierte Einwilligung gemäß Art. 6 Abs. 1 Buchst. a und Art. 9 Abs. 2 Buchst. a der EU Datenschutzgrundverordnung (DSGVO) und Art. 9 Abs. 2 Buchstabe j DSGVO.

Die Bereitstellung Ihrer personenbezogenen Daten ist freiwillig. Ohne Ihre ausdrückliche Einwilligung in die Verarbeitung Ihrer Daten können Sie allerdings nicht an dieser klinischen Studie teilnehmen.

### c) Verantwortlichkeit

Verantwortlich im Sinne des Datenschutzrechts ist Prof. Dr. A. Gumz (Prüfer/Sponsor).

Das lokale Studienzentrum (d.h. Ihr Ausbildungsnstitut) bleibt davon unabhängig für Ihre Behandlungsdaten verantwortlich (unkodierte Patientendaten).

### d) Zweck(e)

Mit Hilfe der erhobenen Daten soll die Wirksamkeit eines neuen Trainings- und Supervisionsansatzes mit einem spezifischen Fokus auf der therapeutischen Beziehung im Vergleich zur regulären Psychotherapieausbildung für Therapeuten in Ausbildung und die von ihnen behandelten Patienten mit einer depressiven Störung klinisch untersucht werden. Gleichzeitig erforschen wir auch, wie sich therapeutische Veränderung in den Therapieprozessen konkret ereignet, welches therapeutische Vorgehen und welche Patienten- und Therapeutenmerkmale mit besserem Therapieerfolg einhergehen. Wir prüfen, wie sich hilfreiche Sitzungen von weniger hilfreichen Sitzungen unterscheiden. Dabei betrachten wir Merkmale der therapeutischen Beziehungsgestaltung, Eigenschaften von Therapeuten und Patienten, angewandte Techniken sowie sprachliche und nonverbale Merkmale (z.B. Stimme oder Bewegungsverhalten).

### e) Weitergabe/Empfänger

Die von Ihnen erhobenen Daten werden, soweit erforderlich, pseudonymisiert weitergegeben an:

1. vom Prüfer/Sponsor beauftragte Stellen zum Zweck der Durchführung und wissenschaftlichen Auswertung,
2. im Falle unerwünschter Ereignisse: vom Prüfer/Sponsor an zuständige Überwachungsbehörden im Rahmen von Inspektionen oder Beauftragte des Prüfers/Sponsors (s.g. Auditoren oder Monitore)

Die von Ihnen im Rahmen der oben genannten klinischen Studie erhobenen und gespeicherten Daten (auch die originalen Klardaten) können soweit erforderlich und gesetzlich erlaubt, durch Beauftragte des Sponsors/zentralen Studienzentrums (s.g. Monitore) zur Überprüfung der ordnungsgemäßen Durchführung der klinischen Studie im zentralen Studienzentrum eingesehen werden. Diese sind zur Vertraulichkeit verpflichtet, eine Weitergabe der erhobenen Daten erfolgt in diesem Zusammenhang nicht.

Im Rahmen dieser klinischen Studie erfolgt eine Weitergabe Ihrer pseudonymisierten Daten zum Zweck der Datenauswertung, Zulassung und Überwachung nur innerhalb der Europäischen Union und des Europäischen Wirtschaftsraumes.

### f) Ihre Rechte

Sie haben grundsätzlich folgende Rechte bezüglich Ihrer personenbezogenen Daten, sofern dies nicht aufgrund einer zwischenzeitlich vorgenommenen Löschung der identifizierenden Merkmale zur Entschlüsselung technisch oder anderweitig gesetzlich unmöglich ist:

**Recht auf Widerruf ihrer Einwilligung**

So wie die Einwilligung zur Teilnahme an der klinischen Studie können Sie auch Ihre Einwilligung zur Verarbeitung der erhobenen Daten jederzeit widerrufen.

Im Falle eines Widerrufs Ihrer Einwilligung werden Ihre Daten unverzüglich gelöscht.

**Sie haben weiterhin folgende Rechte**

Recht auf Auskunft (inkl. unentgeltlicher Überlassung einer Kopie) über Ihre personenbezogenen Daten, die im Rahmen der klinischen Studie erhoben, verarbeitet oder ggf. an Dritte übermittelt werden.

Recht auf Datenübertragung der zu Ihrer Person erhobenen Daten an Sie oder eine von Ihnen bestimmte Stelle.

Recht auf Berichtigung unrichtiger personenbezogener Daten, auf Einschränkung der Verarbeitung und auf Widerspruch gegen die Nutzung der Daten.

**Wahrnehmung Ihrer Rechte**

Wollen Sie von einem oder mehreren der genannten Rechten Gebrauch machen, kontaktieren Sie bitte Ihren Prüfer/Sponsor. Bei Anliegen zur Datenverarbeitung und zur Einhaltung der datenschutzrechtlichen Anforderungen können Sie sich auch an folgende Datenschutzbeauftragte wenden:

DSB Münster GmbH

André Korte

Martin-Luther-King-Weg 42-44

48155 Münster

Tel.: +49 251 71879-110

E-Mail: [**********](mailto:datenschutz@dsb-ms.de)

Sie haben außerdem ein Beschwerderecht bei einer Datenschutzaufsichtsbehörde. Sollten Sie Bedenken hinsichtlich des Umgangs mit Ihren personenbezogenen Daten haben, können Sie sich an folgende Stellen wenden:

Landesbeauftragten für Datenschutz und Informationsfreiheit Nordrhein-Westfalen,

Postfach 20 04 44

40102 Düsseldorf

Tel.: +49 211 38424-0

****************

Eine Liste aller in Deutschland und der Europäischen Union zuständigen Datenschutzaufsichtsbehörden finden Sie hier:

<https://www.bfdi.bund.de/DE/Infothek/Anschriften_Links/anschriften_links-node.html>

### g) Dauer der Speicherung der Daten:

Die erhobenen Daten werden im zentralen Studienzentrum / vom Sponsor für die Dauer von 10 Jahren nach Beendigung oder Abbruch der klinischen Studie gespeichert.

### h) Veröffentlichung

Wissenschaftliche Veröffentlichungen von Ergebnissen (auch open data) erfolgen in einer Form, die keine direkten Rückschlüsse auf Ihre Person zulässt. Alle personenbezogenen Informationen (wie z.B. Alter, Geschlecht, Pseudonym etc.) werden nicht veröffentlicht.

**Prüfstelle:** IFT Psychotherapeutische Ambulanz, Leopoldstr. 175, 80804 München, Dipl.-Psych Sibylle Gmeinwieser, ***********

**Zentrales Studienzentrum:** Professur für Psychosomatik und Psychotherapie, Psychologische Hochschule Berlin (PHB), Am Köllnischen Park 2, 10179 Berlin, a.gumz@phb.de

**Prüfer:** Prof. Dr. Antje Gumz

**Sponsor der klinischen Studie:** Psychologische Hochschule Berlin (PHB), Am Köllnischen Park 2, 10179 Berlin

DRKS number: DRKS00014842

**Einwilligungserklärung**

**Zur Teilnahme an der wissenschaftlichen Studie ,,Randomisiert kontrollierte Multicenter-Studie zur Therapieausbildung“.**Projektnummer 504346851

Ich hatte die Gelegenheit an einer Informationsveranstaltung zur Studie teilzunehmen sowie für ein persönliches Gespräch das Studienteam in Berlin oder den Ansprechpartner, die Ansprechpartnerin für die Studie an meinem Institut zu kontaktieren. Möglicherweise offene Fragen konnte ich in diesem Rahmen zufriedenstellend klären. Ich bin verständlich über Wesen, Bedeutung, Risiken und Tragweite der klinischen Studie aufgeklärt worden. Ich habe darüber hinaus den Text der Studieninformation mit seinen beiden Teilen (Teil I: Informationen zum Studienablauf; Teil II: Informationen zur Verwendung der Daten) gelesen und verstanden. Ich hatte ausreichend Zeit, mich zu entscheiden.

Mir ist bekannt, dass ich jederzeit und ohne Angabe von Gründen meine Einwilligung zur Teilnahme an der Prüfung zurückziehen kann (mündlich oder schriftlich), ohne dass mir daraus Nachteile entstehen.

**Datenschutzrechtliche Einwilligung**

Mir ist bekannt, dass bei dieser klinischen Studie persönliche Informationen über mich erhoben, gespeichert und ausgewertet werden sollen. Die Verwendung meiner personenbezogenen Daten setzt vor der Teilnahme an der klinischen Prüfung folgende freiwillig abgegebene Einwilligungserklärung voraus; ohne die nachfolgende Einwilligung kann ich nicht an der klinischen Studie teilnehmen.

Ich willige ein, dass im Rahmen dieser klinischen Studie persönliche Informationen, Audiodaten und Videodaten (Aufzeichnungen von Therapiesitzungen) über mich erhoben und in Papierform sowie auf elektronischen Datenträgern gemäß den Angaben in der Informationsschrift Teil II 1. aufgezeichnet, verwendet und weitergegeben werden.

**Ich willige freiwillig ein, an der oben genannten klinischen Studie teilzunehmen.**

**Zugleich willige ich in die Verarbeitung meiner personenbezogenen Daten wie beschrieben und von mir angegeben ein.**

Ein Exemplar der Studieninformation und -einwilligung habe ich erhalten. Ein Exemplar verbleibt im zentralen Studienzentrum.

...........................................................................................................................

Name des **Therapeuten**, **der Therapeutin** in Druckbuchstaben

........................................

geb. am

.................................... ..............................................................................................

Ort/Datum Unterschrift des **Therapeuten**, **der Therapeutin**

**Einverständniserklärung zur Kontaktaufnahme**

bei studienbezogenen Anliegen

Sehr geehrte Therapeutin, sehr geehrter Therapeut,

Während der Studie werden Sie mehrmals mittels Fragebögen und zweimalig mittels einer Video-Übung zu herausfordernden Therapiesituationen befragt. Diese Erhebungen finden online statt.

Sie dienen dazu Veränderungen, die durch den neuen Trainings- und Supervisionsansatz im Verlauf entstehen könnten, auch über einen längeren Zeitraum zu beobachten. Die Erhebungen finden 20 Wochen, 35 Wochen, 20 Monate und 36 Monate nach der Randomisierung Ihres ersten Studienpatienten statt (auch wenn die Therapie bereits beendet wurde).

Mit diesem Schreiben möchten wir Sie um Ihr **Einverständnis zur erneuten Kontaktaufnahme und zur Erinnerung an diese Erhebungen**  bitten. Dieses Einverständnis verpflichtet Sie nicht zur Teilnahme an der Erhebung. Das Einverständnis kann jederzeit ohne Begründung zurückgezogen werden.

**Hiermit erkläre ich mich damit einverstanden, dass Mitarbeiter/innen der Psychologischen Hochschule Berlin mich zu den Erhebungszeitpunkten per Email kontaktieren und mir den Zugang dafür schicken können. (Für eine Studienteilnahme ist diese Einwilligung zwingend.)**

ο **ja** ο **nein**

**Hiermit erkläre ich mich damit einverstanden, dass Mitarbeiter/innen der Psychologischen Hochschule Berlin mich jeweils 2 Wochen vor der Erhebung mittels eines kurzen neutralen Textes an den Termin erinnern können.**

ο **ja** ο **nein**

Ich bevorzuge eine Erinnerung auf folgendem Weg:

ο Email ο SMS ο telefonisch

**Hiermit erkläre ich mich damit einverstanden, dass Mitarbeiter/innen der Psychologischen Hochschule Berlin mich während der Studienlaufzeit aus anderen studienbezogenen Gründen (z.B. organisatorischen Probleme) kontaktieren können.**

ο **ja** ο **nein**

....................................................................................
Datum, Unterschrift des **Therapeuten**, **der Therapeutin**

**Bitte geben Sie unbedingt zwei E-Mail-Adressen an**.

............................................................ ........................................................

E-Mail-Adresse 1 E-Mail-Adresse 2

............................................................

Telefonnummer (Angabe freiwillig)

**Auszahlung der Aufwandsentschädigung**

Ich bitte um Auszahlung der Aufwandsentschädigung für die Teilnahme an der Studie auf folgendes Konto:

...........................................................................................................................

**Name und Vorname** der Kontoinhaberin/des Kontoinhabers

...........................................................................................................................

IBAN

...........................................................................................................................

BIC
