# Supplementary material for: Modified Alliance-Focused Training with Doubling as an integrative approach to improve therapists’ competencies in dealing with alliance ruptures and prevent negative outcomes in psychotherapy for depression: study protocol of a randomised controlled multicentre trial
Source: BMJ Open. 2025 Jul 16;15(7):e098343. doi: 10.1136/bmjopen-2024-098343 (PMC12273124; doi:10.1136/bmjopen-2024-098343)
Supplement: online supplemental file 3 [file bmjopen-15-7-s003.docx]

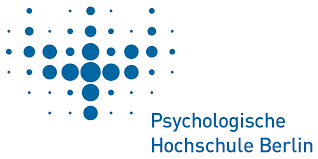
Prof. Dr. Antje Gumz

Professur für Psychosomatik und Psychotherapie

Psychologische Hochschule Berlin (PHB)

Am Köllnischen Park 2

10179 Berlin

**Prüfstelle:** Köln-Bonner Akademie für Verhaltenstherapie (KBAV), Wenzelgasse 35

53111 Bonn, Dr. phil. Lisa Miebach, [************](mailto:l.miebach@mvzpsyche.de)

**Zentrales Studienzentrum:** Professur für Psychosomatik und Psychotherapie, Psychologische Hochschule Berlin (PHB), Am Köllnischen Park 2, 10179 Berlin, [a.gumz@phb.de](mailto:a.gumz@phb.de)

**Prüfer:** Prof. Dr. Antje Gumz

**Sponsor der klinischen Studie:** Psychologische Hochschule Berlin (PHB), Am Köllnischen Park 2, 10179 Berlin

DRKS number: DRKS00014842

**Studieninformation**

**Randomisiert kontrollierte Multicenter-Studie zur Therapieausbildung**
Projektnummer 504346851

Sehr geehrte Supervisorin, sehr geehrter Supervisor,

wir möchten Sie fragen, ob Sie bereit sind, an der nachfolgend beschriebenen klinischen Studie teilzunehmen.

Die klinische Studie, die wir Ihnen hier vorstellen, wurde gemäß der berufsrechtlichen Vorgabe (§15 der Berufsordnung für nordheinische Ärztinnen und Ärtze) beraten, und erhebt keine berufsrechtlichen oder berufsethischen Bedenken und stimmt somit der Durchführung der Studie zu.

Diese klinische Studie wird an mehreren Orten und verschiedenen Psychotherapieausbildungsinstituten durchgeführt; es sollen insgesamt ungefähr 240 Patienten und 120 Therapeuten daran teilnehmen. Die Studie wird durch den oben genannten Sponsor veranlasst und finanziert.

**Ihre Teilnahme an dieser klinischen Studie ist freiwillig.** Sie werden in diese Studie also nur dann einbezogen, wenn Sie dazu schriftlich Ihre Einwilligung erklären. Sofern Sie nicht an der klinischen Studie teilnehmen oder später aus ihr ausscheiden möchten, erwachsen Ihnen daraus keine Nachteile.

Der nachfolgende Text soll Ihnen die Ziele und den Ablauf erläutern. Der Text ist in drei Abschnitte gegliedert:

- Kurzdarstellung der Studie.
- Teil I: Informationen zum Studienablauf
- Teil II: spezifische Informationen zum Datenschutz

Neben dieser schriftlichen Information hatten Sie bereits oder haben Sie die Möglichkeit an einer Informationsveranstaltung zur Studie teilzunehmen sowie zu einem persönlichen Gespräch mit dem Studienteam in Berlin oder dem Ansprechpartner, der Ansprechpartnerin für die Studie an Ihrem Institut. Bitte zögern Sie nicht, alle Punkte anzusprechen, die Ihnen unklar sind. Sie können gerne Sätze/Abschnitte markieren, die Sie nicht verstanden haben, um sie zu besprechen. Nehmen Sie sich ausreichend Bedenkzeit, um über Ihre Teilnahme zu entscheiden.

**Kurzdarstellung der Studie**

**Grund für die Studie**: Ein zu hoher Anteil an Patienten mit Depressionen profitiert nicht ausreichend von Psychotherapie. Um die Ergebnisse von Psychotherapie zu verbessern, ist es entscheidend, die Faktoren, die zum Erfolg beitragen, zu kennen und an ihnen anzusetzen. Ausgehend von bereits bekannten Einflussfaktoren, möchten wir in der Studie untersuchen, wie sich Veränderung in Psychotherapieprozessen ereignet, welches therapeutische Vorgehen und welche Patienten- und Therapeuteneigenschaften dazu beitragen. Wir prüfen, wie sich hilfreiche von weniger hilfreichen Sitzungen unterscheiden und betrachten dabei Merkmale der therapeutischen Beziehungsgestaltung, Eigenschaften des Therapeuten bzw. der Therapeutin und des Patienten bzw. der Patientin, angewandte Techniken sowie sprachliche und nonverbale Merkmale (z.B. Stimme oder Bewegungsverhalten). Zudem prüfen wir einen neuen Trainings- und Supervisionsansatz für Therapeuten, der einen spezifischen Fokus auf einen der bekanntesten Einflussfaktoren für Therapieerfolg, die therapeutische Beziehung, legt. Dieser neue Ansatz soll mit der regulären Therapieausbildung verglichen werden. In die Studie werden Patienten mit Depressionen, Therapeuten in Ausbildung und deren Supervisoren eingeschlossen. Mit den Ergebnissen dieser Studie erhoffen wir uns, einen Beitrag zur Verbesserung der Qualität der Therapieausbildung und der ambulanten Depressionsbehandlung zu leisten.

**Studienablauf:** An der klinischen Studie nehmen Patienten mit der Diagnose einer depressiven Störung, Therapeuten in Ausbildung und Supervisoren teil. Die Studientherapien und -supervisionen sind Teil der Routineausbildung. Die Supervision im Rahmen der Studie wird in Form von Gruppensupervision (3 bis 4 Therapeuten je Gruppe) angeboten und entspricht den für die Psychotherapieausbildung vorgegebenen Rahmenbedingungen in Bezug auf Frequenz und Dosis. Studienpatienten sind erwachsene Patienten mit einer depressiven Störung, die eine ambulante Psychotherapie (Verhaltenstherapie oder tiefenpsychologisch fundierte Therapie) beginnen möchten.

Die Studientherapeuten behandeln je zwei Patienten in der Studie. Die Therapiesitzungen finden, wie üblich, wöchentlich für 50 Minuten statt. Die Dauer der Therapie (Kurzzeittherapie oder Langzeittherapie) wird in Absprache mit der Supervisorin bzw. dem Supervisor gemäß üblichem Vorgehen festgelegt. Die Supervision begleitet die Studientherapeuten über den Therapieverlauf hinweg, sofern keine gegenteiligen institutsspezifischen Regelungen getroffen wurden.

Studiensupervisoren füllen zu Studienbeginn einen Kurzfragebogen (ca. 5 Minuten) aus. Supervisoren der Interventionsgruppe nehmen vor dem Start der Supervisionsgruppe an einem 2-tägigen Workshop zur Schulung der neuen Supervisionsmethode und anschließend monatlich an einem 2-stündigen online Gruppen-Supervisorenmeeting teil.

Patienten und Therapeuten werden den Supervisoren der Interventions- bzw. Kontrollgruppe randomisiert zugeteilt. Supervisoren werden im Hinblick auf zeitliche Passung mit den Workshopterminen und Supervisionszeiten der Interventions- bzw. Kontrollgruppe zugeteilt.

Patienten und Therapeuten füllen zu Studienbeginn und über den Therapieverlauf hinweg Fragebögen aus und nehmen zu ausgewählten Zeitpunkten an einem Telefoninterview (Patienten) bzw. einer Online-Übung zu herausfordernden Therapiesituationen (Therapeuten) teil. Alle Therapiesitzungen werden videoaufgezeichnet. Supervisoren der Interventionsgruppe nutzen die Videos in ihrer Supervision.

**Möglicher Nutzen für Sie:** Wir erhoffen uns aufgrund bisheriger Forschungsergebnisse, dass der neue Ausbildungs- und Supervisionsansatz im Vergleich zur regulären Psychotherapieausbildung die Therapieergebnisse der Patienten zusätzlich verbessern kann. Als Supervisor, Supervisorin der Interventionsgruppe erhalten Sie die Möglichkeit, den neuen Supervisionsansatz kennenzulernen. Als Supervisor, Supervisorin der Kontrollgruppe haben Sie die Möglichkeit, den Supervisionansatz nach Abschluss der Studie kennzulernen. Die Teilnahme am Workshop und dem anschließenden Gruppen-Supervisorenmeeting ist kostenlos für Sie. Fortbildungspunkte werden beantragt. Für Ihren Aufwand im Rahmen der Studie erhalten Sie zusätzlich zum gewohnten Supervisionshonorar 952€ (inkl. MwSt).

**Risiken und Belastungen:** Das Anwenden der neuen Supervisionsmethode kann als ungewohnt und dadurch aufwändig empfunden werden. Das intensive Nachdenken und Nachempfinden der Muster der Beziehungsgestaltung im Rahmen der Supervision kann gelegentlich auch ein wenig anstrengend oder emotional aufwühlend erlebt werden. Wir erwarten keine weiteren Risiken oder unerwünschten Ereignisse aufgrund der Studienteilnahme.

**Freiwilligkeit:** Es ist Ihre freie Entscheidung, ob Sie an dieser Studie teilnehmen möchten oder nicht. Sie werden nur dann einbezogen, wenn Sie dazu schriftlich Ihre Einwilligung erkläre. Sie können jederzeit, auch ohne Angabe von Gründen, Ihre Einwilligung mündlich oder schriftlich widerrufen.

Zusätzlich zur schriftlichen Information werden Sie im Rahmen von Informationsveranstaltungen mündlich aufgeklärt. Bei offen Fragen oder Schwierigkeiten können sie sich vor und während Studienteilnahme jederzeit an das Studienteam (unter studie@phb.de), die Studienleiterin, Prof. A. Gumz (a.gumz@phb.de) oder die Studienverantwortlichen an Ihrem Institut (Liste mit Namen und Kontaktdaten beiliegend) wenden. Wenn Sie sich für die Teilnahme entscheiden, füllen Sie bitte die Einwilligungserklärung aus.

# **Teil I: Informationen zum Ablauf der klinischen Studie**

**I. 1. Warum wird diese Prüfung durchgeführt?**

Depressive Störungen gehören zu den häufigsten Erkrankungen. Der Verlauf der Erkrankung ist häufig wiederkehrend oder chronisch und die Folgen für den Einzelnen und die Gesellschaft können schwerwiegend sein. Verhaltenstherapien und tiefenpsychologisch fundierte Psychotherapien sind wissenschaftlich geprüfte, wirksame Therapieverfahren.

Trotz der generellen guten Wirksamkeit dieser Therapieverfahren, gibt es viele depressive Patienten, die nicht ausreichend von ihrer Psychotherapie profitieren und viele Patienten brechen ihre Psychotherapie vorzeitig ab. Damit noch mehr Patienten von einer Psychotherapie profitieren, ist es wichtig zu untersuchen, welche Faktoren zum Therapieerfolg beitragen. Ob eine Therapie wirksam ist, hängt von verschiedenen therapieprozessbezogenen, patientenseitigen und therapeutenseitigen Faktoren ab. Ein bekanntermaßen entscheidender Faktor ist, ob es gelingt, eine hilfreiche und vertrauensvolle Therapiebeziehung herzustellen und aufrechtzuerhalten.

Wir möchten in unserer Studie daher auch untersuchen, wie ein neuer Trainings- und Supervisionsansatz mit einem spezifischen Fokus auf der Herstellung oder Aufrechterhaltung einer guten therapeutischen Beziehung im Vergleich zur regulären Psychotherapieausbildung wirkt. Geichzeitig erforschen wir, wie sich therapeutische Veränderung in den Therapieprozessen konkret ereignet, welches therapeutische Vorgehen und welche Patienten- und Therapeutenmerkmale mit besserem Therapieerfolg einhergehen. Wir prüfen, wie sich hilfreiche Situngen von weniger hilfreichen Sitzungen unterscheiden. Dabei betrachten wir Merkmale der therapeutischen Beziehungsgestaltung, Eigenschaften und angewandte Techniken der Therapeuten sowie sprachliche und nonverbale Merkmale (z.B. Stimme oder Bewegungsverhalten).

Von der Durchführung der vorgesehenen Studie erhoffen wir uns, einen Beitrag zur Qualität der Psychotherapieausbildung und hierüber zur Verbesserung der ambulanten Depressionsbehandlung zu leisten.

**I. 2. Was muss ich bezüglich der Randomisierung in der Studie zwingend beachten?**

Im Rahmen dieser klinischen Studie wird ein neuer Trainings- und Supervisionsansatz mit einem spezifischen Fokus auf der Herstellung oder Aufrechterhaltung einer guten therapeutischen Beziehung mit regulärer Psychotherapieausbildung verglichen. Zu diesem Zwecke findet eine Randomisierung statt. Sowohl die Studientherapeuten als auch die Studienpatienten werden randomisiert. Die Supervisoren werden nicht randomisiert. Sie werden im Hinblick auf zeitliche Passung mit den Workshopterminen und Supervisionszeiten der Interventions- bzw. Kontrollgruppe zugeordnet. Nach Randomisierung der Therapeutinnen und Therapeuten wird Ihnen als Supervisor bzw. Supervisorin mitgeteilt, wer sich in Ihrer Gruppe befindet.

Zur objektiven Gewinnung von Studiendaten ist es notwendig, dass die Intervention nicht Teilnehmer der Kontrollgruppe erreicht und dass die Studienpatienten keinesfalls erfahren, welcher Studienbedingung Sie zugeordnet wurden (d.h. die Patientin, der Patient muss verblindet bleiben). Um dies sicherzustellen, bitten wir Sie die folgenden Regelungen strengstmöglich einzuhalten.

**Wichtige Regelungen zur Gewährleistung der Studienqualität:**

In der Studie könnten methodische Verzerrungen entstehen, wenn Teilnehmende der Kontrollgruppe (Therapeuten, Supervisoren, Patienten) Informationen über Inhalte und Vorgehen der Intervention erhalten. Das betrifft zum einen schriftliches Material und zum anderen die Kommunikation über die Intervention. Wir bitten Sie daher über die Studienlaufzeit hinweg abgesehen von dem Äußern eines allgemeinen persönlichen Eindrucks (z.B. „*hilfreich, ganz interessant, etwas ungewohnt, passt oder passt nicht so gut zu meinem Stil*“ etc.) **nicht mit Kolleginnen an Ihrem Ausbildungsinstitut über die Intervention zu sprechen**. Zudem ist es wichtig, dass Sie über den genannten Zeitraum **keine Therapeuten der Kontrollgruppe in Supervision nehmen**, wenn Sie selbst in der Interventionsgruppe sind. Dazu erhalten Sie vom Studienteam Listen mit Namen der entsprechenden Studientherapeuten, sodas sie jederzeit bei Anfragen prüfen können, ob eine Supervisandin, ein Supervisand ggf. abgelehnt werden muss.

Alle Therapeuten und Supervisoren der Interventionsgruppe verpflichten sich **generell**, den Inhalt der Intervention (Workshop, Material, Vorgehen, Supervision) bis zum Abschluss der Studie **streng vertraulich** zu behandeln. Dies betrifft bspw. auch die Weiterverbreitung innerhalb von Fort- oder Weiterbildungen, die über die Studienlaufzeit hinweg streng untersagt ist.

Die Therapeuten (und die Supervisoren, sollten diese Kontakt zu den Patienten haben) dürfen über ihre Zuteilung oder über die Intervention **nicht** mit ihren Patienten sprechen.

**Wir bitten die Supervisoren beider Gruppen, Mitverantwortung für die bestmögliche Einhaltung dieser Regelungen zur Verschwiegenheit zu übernehmen, da die Qualität der Studie entscheidend davon abhängt.**

**I. 3. Wie ist der Ablauf der Studie und was muss ich bei Teilnahme beachten?**

An der klinischen Studie nehmen erwachsene Patienten mit der Diagnose einer depressiven Störung, Therapeuten in Ausbildung (Verhaltenstherapie oder tiefenpsychologisch fundierte Therapie) und Supervisoren teil. Die Studientherapien sind Teil der Routineausbildung, d.h. sie werden als Ausbildungsfälle anerkannt. Die Supervision im Rahmen der Studie wird in Form von Gruppensupervision (3 bis 4 Therapeuten je Gruppe, 2 Patienten je Therapeut/in) angeboten und entspricht den für die Psychotherapieausbildung vorgegebenen Rahmenbedingungen in Bezug auf Frequenz und Dosis. Üblicherweise werden 20 bis 25 Minuten pro Monat pro Therapieverlauf bzw. Patientin, Patient eingeplant. Die Supervision kann monatlich, zwei-wöchentlich und in selteneren Fällen auch wöchentlich angeboten werden. Die Supervision begleitet die Studientherapeuten über den Therapieverlauf hinweg, sofern keine gegenteiligen institutsspezifischen Regelungen getroffen wurden. Die spezifischen Rahmenbedingungen für die Gruppensupervision wurden mit Ihrem Institut abgesprochen, um die Anerkennung am Institut zu gewährleisten. Über die hier genannten Rahmenbedingungen hinaus ist es wichtig, dass diese Regelungen, über die Sie vorab informiert wurden, eingehalten werden und dass die Rahmenbedingungen in Bezug auf Frequenz und Dosis in der Interventions- und Kontrollgruppe identisch sind. Sie werden dafür verantwortlich sein, die Anwesenheiten bei den Gruppensupervisionen zu dokumentieren. Bitte beachten Sie, dass die Anwesenheiten jedes Mal doppelt dokumentiert werden müssen (für die Studie und für das Institut).

Patienten und Therapeuten werden den Supervisoren der Interventions- bzw. Kontrollgruppe randomisiert zugeteilt. Die Supervisoren werden im Hinblick auf zeitliche Passung mit den Workshopterminen und Supervisionszeiten der Interventions- bzw. Kontrollgruppe zugeteilt.

Alle Supervisoren füllen zu Studienbeginn einen Kurzfragebogen (ca. 5 Minuten) aus. Supervisoren der Interventionsgruppe nehmen vor dem Start der Supervisionsgruppe an einem 2-tägigen Workshop zur Schulung in der neuen Supervisionsmethode und einem anschließenden monatlichen 2-stündigen online Gruppen-Supervisorenmeeting teil.

Das Studienteam prüft die Ein- und Ausschlusskriterien für eine Studienteilnahme bei den Patienten und Therapeuten. Patienten und Therapeuten absolvieren verschiedene Erhebungen (mittels Fragebögen sowie zusätzlich bei Patienten mittels Telefoninterviews und bei Therapeuten mittels einer Video-Übung zu herausfordernden Therapiesituationen) über den Studienverlauf (36 Monate) hinweg.

Die Therapiesitzungen finden, wie üblich, wöchentlich für 50 Minuten statt. Die Dauer der Therapie (Kurzzeittherapie oder Langzeittherapie) wird in Absprache mit der Supervisorin bzw. dem Supervisor gemäß üblichem Vorgehen festgelegt. Alle Therapiesitzungen werden videoaufgezeichnet. Therapeuten, die der Interventionsgruppe angehören, nutzen die Videoaufzeichnungen regelmäßig in der Supervision. Einige Sitzungen aus dem Therapieverlauf werden zusätzlich für Forschungszwecke genutzt.

**Die Therapeuten erhalten ohne Ausnahme keinen Einblick in die Daten der Patienten und die Patienten erhalten keinen Einblick in die Daten der Therapeuten!**

**I. 4. Welchen persönlichen Nutzen habe ich von der Teilnahme an der Studie?**

Wir erhoffen uns aufgrund bisheriger Forschungsergebnisse, dass ein neuer Ausbildungs- und Supervisionsansatz im Vergleich zur regulären Psychotherapieausbildung die Therapieergebnisse der Patienten zusätzlich verbessern kann. Als Supervisor, Superevisorin der Interventionsgruppe erhalten Sie die Möglichkeit, den neuen Supervisionsansatz kennenzulernen. Als Supervisor, Superevisorin der Kontrollegruppe haben Sie die Möglichkeit, den Supervisionansatz nach Abschluss der Studie kennzulernen. Die Teilnahme am Workshop ist kostenlos für Sie. Fortbildungspunkte werden beantragt. Für Ihren Aufwand im Rahmen der Studie erhalten Sie zusätzlich zu dem gewohnten Supervisionshonorar 952€ (inkl. MwSt).

**I.** **5. Welche gesundheitlichen Risiken und Belastungen sind mit der Teilnahme an der Studie verbunden?**

Das Anwenden der neuen Supervisionsmethode kann als ungewohnt und dadurch aufwändig empfunden werden. Das intensive Nachdenken und Nachempfinden der Muster der Beziehungsgestaltung im Rahmen der Supervision kann gelegentlich auch ein wenig anstrengend oder emotional aufwühlend erlebt werden. Wir erwarten keine weiteren Risiken oder unerwünschten Ereignisse aufgrund der Studienteilnahme.

**I. 6. Wer darf an dieser klinischen Studie nicht teilnehmen?**

Sie können an dieser klinischen Studie nur teilnehmen, wenn Sie gesund sind und sich nicht gleichzeitig für andere klinische Prüfungen oder andere klinische Forschungsprojekte zur Verfügung stellen*.*

**I. 7. Entstehen für mich Kosten durch die Teilnahme an der klinischen Studie? Erhalte ich eine Aufwandsentschädigung?**

Durch Ihre Teilnahme an dieser klinischen Prüfung entstehen für Sie keine Kosten*.*

Für Ihre Teilnahme an dieser Studie erhalten Sie eine Aufwandsentschädigung entsprechend den folgenden Bedingungen: Alle teilnehmenden Supervisoren erhalten bei vollständiger Studienteilnahme 952 € inkl. inkl. MwSt 19%. Die Supervisoren der Interventionsgruppe können kostenlos am Workshop und den anschließenden Online-Meetings teilnehmen und bekommen die Reise- und Übernachtungskosten erstattet, wenn der Workshop an einem Ort außerhalb des Wohnortes bzw. des Ortes, an dem sich das zugehörige Ausbildungsinstitut befindet, stattfindet (Bahnfahrt 2. Klasse, zzgl. Kosten für eine Übernachtung in Höhe des für den öffentlichen Dienst üblichen Rahmens).

Supervisoren der Kontrollgruppe erhalten bei Interesse den Workshop kostenlos nach dem Studienende. Fortbildungspunkte werden beantragt.

**I. 8. Werden mir neue Erkenntnisse zu der klinischen Prüfung mitgeteilt?**

Sie werden während Ihrer Teilnahme über neue Erkenntnisse in Bezug auf diese Studie informiert, die für Ihre Bereitschaft zur weiteren Teilnahme wesentlich sein können.

# Nach Beendigung der gesamten Studie wird das Studienteam zusammenfassende Ergebnisse in der Datenbank des Deutschen Registers Klinischer Studien (<https://drks.de/search/de/results>) bereitstellen. Dies kann von Ihnen unter der oben angegebenen trial number eingesehen werden. Sie können sich nach Studienende zur Information über die Studienergebnisse zudem gerne an das Studienteam wenden.

**I. 9. Wer entscheidet, ob ich aus der klinischen Prüfung ausscheide?**

**Sie können jederzeit, auch ohne Angabe von Gründen, Ihre Teilnahme beenden, ohne dass Ihnen dadurch Nachteile entstehen.**

Es ist auch möglich, dass der Sponsor entscheidet, Ihre Teilnahme an der klinischen Studie vorzeitig zu beenden. Ein möglicher Grund dafür kann sein, dass die gesamte klinische Studie abgebrochen werden muss (z.B. falls ein Zusammenhang zwischen dem Vorkommen schwerwiegender unerwünschter Ereignisse und der Studienteilnahme festgestellt wird).

**I. 10. An wen wende ich mich bei weiteren Fragen?**

Bei offen Fragen oder Schwierigkeiten können sie sich vor und während Studientilnahme jederzeit an das zentrale Studienzentrum ([studie@phb.de](mailto:studie@phb.de)), die Studienleiterin Prof. A. Gumz ([a.gumz@phb.de](mailto:a.gumz@phb.de)) oder die Studienverantwortlichen an Ihrem Institut (Liste mit Namen und Kontaktdaten beiliegend) wenden.

# **Teil II: Informationen zum Datenschutz**

**II. 1. Was geschieht mit den über mich erhobenen Daten?**

###

### a) Allgemeine Informationen

Während der klinischen Studie werden persönliche Informationen von Ihnen erhoben und im zentralen Studienzentrum (an der Psychologischen Hochschule Berlin) gesichert gelagert (in verschlossenen Schränken). Dies trifft auf die Einwilligungserklärung, die Ihren Namen und weitere persönliche Informationen enthält, sowie bei Teilnahme an der Interventionsgruppe auf die Anwesenheitslisten für den Workshop und die Online-Meetings zu. Die namensbasierte Anwesenheitsdokumention wird in eine pseudonymisierte Form übertragen, auf diese Weise elektronisch gespeichert und anschließend werden die namensbezogenen Anwesenheitslisten gelöscht.

Der beiliegende Kurzfragebogen wird in pseudonymisierter Form erhoben, verarbeitet und gespeichert. Pseudonymisiert bedeutet, dass keine Angaben, mit denen Sie direkt identifiziert werden können (z.B. Namen, Kontaktinformationen, Geburtsdatum, etc.) verwendet werden, sondern nur ein Nummern- und Buchstabencode. Dieser Code, d.h. die Supervisoren-ID, besteht aus neun Zeichen und setzt sich folgendermaßen zusammen: a) 2 Ziffern, die Ihr Institut kennzeichnen; b) S (für Supervisor); c) eine fortlaufende dreistellige Ziffer beginnend mit 1001; d) die ersten zwei Buchstaben des Vornamens der Mutter, und e) der erste Buchstabe Ihres Geburtsortes. **Wir bitten Sie, die letzten drei Stellen (d und e) auf dem beiliegenden Kurzbogen dementsprechend zu vervollständigen.**

Der Datenschlüssel, der eine Zuordnung der Studiencodes zu den tatsächlichen Therapeuten-

, Patienten- und Supervisorenidentitäten enthält, wird während der Studienlaufzeit und bis zu zehn Jahre nach Ende der Studienlaufzeit im zentralen Studienzentrum (Psychologische Hochschule Berlin, PHB) gesichert (d.h. elektronisch verschlüsselt oder im Fall von physischen Kopien in einem verschlossenen Schrank) aufbewahrt. Der Schlüssel wird jederzeit getrennt von allen anderen Studiendaten gelagert. Die schriftliche Kommunikation zwischen den lokalen Studienzentren (Ausbildungsinstituten) und dem zentralen Studienteam (PHB) erfolgt ausschließlich über die Studiencodes. In definierten Ausnahmefällen (bei organisatorischen Problemen) werden Namen in der Kommunikation zwischen lokalen Studienverantwortlichen und zentralem Studienteam verwendet. Dies geschieht jedoch ausschließlich über telefonische Kontakte und um die Pseudonymisierung zu erhalten ausnahmslos ohne Nennung von Studiencodes.

Zugang zu dem „Schlüssel“, der eine persönliche Zuordnung des Studienteilnehmers, der Studienteilnehmerin ermöglicht, haben nur von der Studienleiterin Prof. Dr. Antje Gumz ausdrücklich dazu autorisierte Projektmitarbeiter, die zum Kernstudienteam gehören und die der Schweigepflicht unterliegen. Eine Entschlüsselung erfolgt nur, aus spezifischen studienbezogenen Gründen (z.B. um die namensbezogene Anwesenheitsdokumention in eine pseudonymisierte Form zu übertragen, bei Kontaktaufnahmen). Der individuelle Datenschlüssel wird zehn Jahre nach Ende der Studienlaufzeit dauerhaft gelöscht. Die sichere Aufbewahrung des Schlüssels über diese Zeit hinweg ist nötig, damit das Studienteam sicherheitsrelevante Daten auch nach dem Ablauf der Studie noch zuordnen kann.

Trotz der Pseudonymisierung lässt sich niemals völlig ausschließen, dass auch ohne den Datenschlüssel Rückschlüsse auf Ihre Person gezogen werden können.

### b) Rechtsgrundlage

Rechtsgrundlage für die Datenverarbeitung ist Ihre informierte Einwilligung gemäß Art. 6 Abs. 1 Buchst. a und Art. 9 Abs. 2 Buchst. a der EU Datenschutzgrundverordnung (DSGVO) und Art. 9 Abs. 2 Buchstabe j DSGVO.

Die Bereitstellung Ihrer personenbezogenen Daten ist freiwillig. Ohne Ihre ausdrückliche Einwilligung in die Verarbeitung Ihrer Daten können Sie allerdings nicht an dieser klinischen Studie teilnehmen.

### c) Verantwortlichkeit

Verantwortlich im Sinne des Datenschutzrechts ist Prof. Dr. A. Gumz (Prüfer/Sponsor).

Das lokale Studienzentrum (d.h. Ihr Ausbildungsnstitut) bleibt davon unabhängig für die Ausbildungs- und Behandlungsdaten verantwortlich (unkodierte Patientendaten, Ausbildungsdaten der Therapeuten).

### d) Zweck(e)

Mit Hilfe der erhobenen Daten soll die Wirksamkeit eines neuen Trainings- und Supervisionsansatzes mit einem spezifischen Fokus auf der therapeutischen Beziehung im Vergleich zur regulären Psychotherapieausbildung für Therapeuten in Ausbildung und die von ihnen behandelten Patienten mit einer depressiven Störung klinisch untersucht werden. Gleichzeitig erforschen wir auch, wie sich therapeutische Veränderung in den Therapieprozessen konkret ereignet, welches therapeutische Vorgehen und welche Patienten- und Therapeutenmerkmale mit besserem Therapieerfolg einhergehen. Wir prüfen, wie sich hilfreiche Sitzungen von weniger hilfreichen Sitzungen unterscheiden. Dabei betrachten wir Merkmale der therapeutischen Beziehungsgestaltung, Eigenschaften von Therapeuten und Patienten, angewandte Techniken sowie sprachliche und nonverbale Merkmale (z.B. Stimme oder Bewegungsverhalten).

### e) Weitergabe/Empfänger

Die von Ihnen erhobenen Daten werden, soweit erforderlich, pseudonymisiert weitergegeben an vom Prüfer/Sponsor beauftragte Stellen zum Zweck der Durchführung und wissenschaftlichen Auswertung.

Die von Ihnen im Rahmen der oben genannten klinischen Studie erhobenen und gespeicherten Daten (auch die originalen Klardaten) können soweit erforderlich und gesetzlich erlaubt, durch Beauftragte des Sponsors/zentralen Studienzentrums (s.g. Monitore) zur Überprüfung der ordnungsgemäßen Durchführung der klinischen Studie in der Prüfstelle eingesehen werden. Diese sind zur Vertraulichkeit verpflichtet, eine Weitergabe der erhobenen Daten erfolgt in diesem Zusammenhang nicht.

Im Rahmen dieser klinischen Studie erfolgt eine Weitergabe Ihrer pseudonymisierten Daten zum Zweck der Datenauswertung, Zulassung und Überwachung nur innerhalb der Europäischen Union und des Europäischen Wirtschaftsraumes.

### f) Ihre Rechte

Sie haben grundsätzlich folgende Rechte bezüglich Ihrer personenbezogenen Daten, sofern dies nicht aufgrund einer zwischenzeitlich vorgenommenen Löschung der identifizierenden Merkmale zur Entschlüsselung technisch oder anderweitig gesetzlich unmöglich ist:

**Recht auf Widerruf ihrer Einwilligung**

So wie die Einwilligung zur Teilnahme an der klinischen Studie können Sie auch Ihre Einwilligung zur Verarbeitung der erhobenen Daten jederzeit widerrufen.

Im Falle eines Widerrufs Ihrer Einwilligung werden Ihre Daten unverzüglich gelöscht.

**Sie haben weiterhin folgende Rechte**

Recht auf Auskunft (inkl. unentgeltlicher Überlassung einer Kopie) über Ihre personenbezogenen Daten, die im Rahmen der klinischen Studie erhoben, verarbeitet oder ggf. an Dritte übermittelt werden.

Recht auf Datenübertragung der zu Ihrer Person erhobenen Daten an Sie oder eine von Ihnen bestimmte Stelle.

Recht auf Berichtigung unrichtiger personenbezogener Daten, auf Einschränkung der Verarbeitung und auf Widerspruch gegen die Nutzung der Daten.

**Wahrnehmung Ihrer Rechte**

Wollen Sie von einem oder mehreren der genannten Rechten Gebrauch machen, kontaktieren Sie bitte Ihren Prüfer/Sponsor. Bei Anliegen zur Datenverarbeitung und zur Einhaltung der datenschutzrechtlichen Anforderungen können Sie sich auch an folgende Datenschutzbeauftragte wenden:

Datenschutzbeauftragter der Prüfstelle/des Sponsors:

Herr Marko Walther

Psychologische Hochschule Berlin

Am Köllnischen Park 2

10179 Berlin

************

Sie haben außerdem ein Beschwerderecht bei einer Datenschutzaufsichtsbehörde. Sollten Sie Bedenken hinsichtlich des Umgangs mit Ihren personenbezogenen Daten haben, können Sie sich an folgende Stellen wenden:

Institut für Psychotherapie Potsdam (IfP)

Rosa-Luxemburg-Straße 40

14482 Potsdam-Babelsberg

Tel. 0331 27 97 57 27

[info@ifp.potsdam.com](mailto:info@ifp.potsdam.com)

Eine Liste aller in Deutschland und der Europäischen Union zuständigen Datenschutzaufsichtsbehörden finden Sie hier:

<https://www.bfdi.bund.de/DE/Infothek/Anschriften_Links/anschriften_links-node.html>

### g) Dauer der Speicherung der Daten:

Die erhobenen Daten werden von der Prüfstelle und dem Sponsor für die Dauer von 10 Jahren nach Beendigung oder Abbruch der klinischen Studie gespeichert.

### h) Veröffentlichung

Wissenschaftliche Veröffentlichungen von Ergebnissen (auch open data) erfolgen in einer Form, die keine direkten Rückschlüsse auf Ihre Person zulässt. Alle personenbezogenen Informationen (wie z.B. Alter, Geschlecht, Pseudonym etc.) werden nicht veröffentlicht.

**Prüfstelle:** Institut für Psychotherapie Potsdam, Rosa-Luxemburg-Straße 40, 14482

Potsdam, Doreen Ruthenberg, [**************](mailto:DRuthenberg@ifp-potsdam.com)

**Zentrales Studienzentrum:** Professur für Psychosomatik und Psychotherapie, Psychologische Hochschule Berlin (PHB), Am Köllnischen Park 2, 10179 Berlin, [a.gumz@phb.de](mailto:a.gumz@phb.de)

**Prüfer:** Prof. Dr. Antje Gumz

**Sponsor der klinischen Studie:** Psychologische Hochschule Berlin (PHB), Am Köllnischen Park 2, 10179 Berlin

DRKS number: DRKS00014842

**Einwilligungserklärung**

**Zur Teilnahme als Supervisor/in an der wissenschaftlichen Studie ,,Randomisiert kontrollierte Multicenter-Studie zur Therapieausbildung“.**Projektnummer 504346851

Ich hatte die Gelegenheit an einer Informationsveranstaltung zur Studie teilzunehmen sowie für ein persönliches Gespräch das Studienteam in Berlin oder den Ansprechpartner für die Studie an meinem Institut zu kontaktieren. Möglicherweise offene Fragen konnte ich in diesem Rahmen zufriedenstellend klären. Ich bin verständlich über Wesen, Bedeutung, Risiken und Tragweite der klinischen Studie aufgeklärt worden. Ich habe darüber hinaus den Text der Studieninformation mit seinen beiden Teilen (Teil I: Informationen zum Studienablauf; Teil II: Informationen zur Verwendung der Daten) gelesen und verstanden.

Ich hatte ausreichend Zeit, mich zu entscheiden.

Mir ist bekannt, dass ich jederzeit und ohne Angabe von Gründen meine Einwilligung zur Teilnahme an der Studie zurückziehen kann (mündlich oder schriftlich), ohne dass mir daraus Nachteile entstehen.

**Datenschutzrechtliche Einwilligung**

Mir ist bekannt, dass bei dieser klinischen Studie **personenbezogene Daten** über mich erhoben, gespeichert und ausgewertet werden sollen. Die Verwendung meiner personenbezogenen Daten setzt vor der Teilnahme an der klinischen Prüfung folgende freiwillig abgegebene Einwilligungserklärung voraus; ohne die nachfolgende Einwilligung kann ich nicht an der klinischen Prüfung teilnehmen.

Ich willige ein, dass im Rahmen dieser klinischen Studie personenbezogene Daten (z.B. soziodemographische Angaben, Angaben zur klinischen Erfahrung, ggf. Anwesenheit am Workshop) über mich erhoben und in Papierform sowie auf elektronischen Datenträgern gemäß den Angaben in der Informationsschrift Teil II 1. aufgezeichnet, verwendet und weitergegeben werden.

**Ich willige freiwillig ein, an der oben genannten klinischen Studie teilzunehmen.**

**Ich verpflichte mich, den Inhalt der Intervention (Workshop, Material, Vorgehen, Supervision) bis zum Abschluss der Studie streng vertraulich zu behandeln und Stillschweigen darüber zu bewahren. Ich willige ein, über die Studienlaufzeit hinweg keine Ausbildungsteilnehmenden, die der Studienkontrollgruppe angehören, in Supervision zu nehmen, wenn ich selbst in der Interventionsgruppe bin.**

**Zugleich willige ich in die Verarbeitung meiner personenbezogenen Daten wie beschrieben und von mir angegeben ein.**

Ein Exemplar der Studieninformation und -einwilligung habe ich erhalten. Ein Exemplar verbleibt in der Prüfstelle.

...........................................................................................................................

Name des **Supervisor, der Supervisorin** in Druckbuchstaben

........................................

geb. am

.................................... ..............................................................................................

Ort/Datum Unterschrift des **Supervisor, der Supervisorin**

**Einverständniserklärung zur Kontaktaufnahme**

bei studienbezogenen Anliegen

Sehr geehrte Supervisorin, sehr geehrter Supervisor,

mit diesem Schreiben möchten wir Sie um Ihr **Einverständnis zur erneuten Kontaktaufnahme bei studienbezogenen Anliegen oder organisatorischen Problemen** bitten. Dieses Einverständnis verpflichtet Sie nicht zur Teilnahme an der Erhebung. Das Einverständnis kann jederzeit ohne Begründung zurückgezogen werden.

**Hiermit erkläre ich mich damit einverstanden, dass Mitarbeiter/innen der Psychologischen Hochschule Berlin mich per E-Mail kontaktieren (Für eine Studienteilnahme ist diese Einwilligung zwingend.)**

ο **ja** ο **nein**

Ich bevorzuge eine Kontaktaufnahme auf folgendem Weg:

ο E-Mail ο SMS ο telefonisch

....................................................................................
Datum, Unterschrift des **Supervisor, der Supervisorin**

**E-Mail-Adresse**

............................................................

E-Mail-Adresse 1

............................................................

Telefonnummer (Angabe freiwillig)

**Auszahlung der Aufwandsentschädigung**

Ich bitte um Auszahlung der Aufwandsentschädigung für die Teilnahme an der Studie auf folgendes Konto:

...........................................................................................................................

**Name und Vorname** der Kontoinhaberin/des Kontoinhabers

...........................................................................................................................

IBAN

...........................................................................................................................

BIC
