# Supplementary material for: Modified Alliance-Focused Training with Doubling as an integrative approach to improve therapists’ competencies in dealing with alliance ruptures and prevent negative outcomes in psychotherapy for depression: study protocol of a randomised controlled multicentre trial
Source: BMJ Open. 2025 Jul 16;15(7):e098343. doi: 10.1136/bmjopen-2024-098343 (PMC12273124; doi:10.1136/bmjopen-2024-098343)
Supplement: online supplemental file 4 [file bmjopen-15-7-s004.docx]

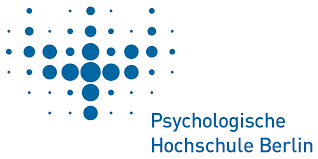
Prof. Dr. Antje Gumz

Professur für Psychosomatik und Psychotherapie

Psychologische Hochschule Berlin (PHB)

Am Köllnischen Park 2

10179 Berlin

**Prüfstelle:** Köln-Bonner Akademie für Verhaltenstherapie (KBAV), Wenzelgasse 35

53111 Bonn, Dr. phil. Lisa Miebach, *************

**Zentrales Studienteam:** Professur für Psychosomatik und Psychotherapie, Psychologische Hochschule Berlin (PHB), Am Köllnischen Park 2, 10179 Berlin, [a.gumz@phb.de](mailto:a.gumz@phb.de)

**Prüfer:** Prof. Dr. Antje Gumz

**Sponsor der klinischen Studie:** Psychologische Hochschule Berlin (PHB), Am Köllnischen Park 2, 10179 Berlin

DRKS number: DRKS00014842

**Studieninformation**

**Randomisiert kontrollierte Multicenter-Studie zur Therapieausbildung**
Projektnummer 504346851

Sehr geehrte Studientherapeutin, sehr geehrter Studientherapeut,

wir bedanken uns sehr herzlich für Ihre Bereitschaft an unserer Studie teilzunehmen. Sie haben die Einwilligung zur Studie unterzeichnet und haben bereits an der Überprüfung der Einschlusskriterien für die Studie und an der anschließenden ersten ausführlichen Messungen mittels Fragebögen und Video-Übung teilgenommen.

Die anschließende Randomisierung hat ergeben, dass Sie der **Interventionsgruppe** zugeordnet wurden.

Wie Sie wissen, wird im Rahmen unserer klinischen Prüfung ein neuer Trainings- und Supervisionsansatz mit einem spezifischen Fokus auf der Herstellung oder Aufrechterhaltung einer guten therapeutischen Beziehung mit regulärer Psychotherapieausbildung verglichen. Studientherapeuten der Interventionsgruppe erhalten den neuen Trainings- und Supervisionsansatz. Sie erhalten zudem Supervision, bei einer Supervisorin bzw. einem Supervisor der gleichen Studienbedingung. Supervisoren der Interventionsgruppe werden ebenfalls im neuen Trainings- und Supervisionsansatz geschult.

Mit vorliegendem Informormationsschreiben möchten wir Ihnen zum einen a) den Trainings- und Supervisionsansatz kurz vorstellen und zum anderen b) auf einige wichtige Regelungen hinweisen, die wir Sie als Studientherapeut/in bitten zu beachten, damit die Qualität der Studie gewährleistet bleibt.

**a) Vorstellen des Trainings- und Supervisionsansatzes:**

Der neue Trainings- und Supervisionsansatz, das sogenannte „Modifizierte Allianzfokussierte Training mit Doppeln“ (MAFT-D, Gumz 2024, 2023, 2020, 2019, Gumz et al. 2020, 2018, Safran u. Muran 2000) setzt an am Umgang mit Spannungen und Krisen in der therapeutischen Beziehung (Alliance Ruptures). Alliance Ruptures ereignen sich zwangsläufig im Verlauf jeder Psychotherapie. Sie bergen ein hohes Risiko für vorzeitige Therapieabbrüche und schlechte Therapieergebnisse. Therapeuten gelingt es oft nicht ausreichend, die Spannungen und Krisen sensibel wahrzunehmen oder es fällt ihnen schwer, konstruktiv damit umzugehen.

Im Rahmen des Trainings werden drei therapeutische Skills geschult: das achtsame Wahrnehmen der im Hier und Jetzt der therapeutischen Beziehung erlebten Affekte, die Fähigkeit, schwierig zu handhabende Affekte zu tolerieren und die Fähigkeit, mit den Patienten in einer hilfreichen Art über das Beziehungsgeschehen zu kommunizieren.

MAFT-D besteht aus einem einführenden zweitägigen Workshop und einer anschließenden spezifischen Gruppensupervision. Im Workshop wird der Umgang mit Spannungen und Krisen in der Therapiebeziehung intensiv geübt. Es werden Indikatoren von Spannungen vermittelt sowie wichtige Haltungen und Techniken für den Umgang mit den Spannungen und Krisen. Die Inhalte werden an Videobeispielen veranschaulicht. Im Zentrum des Workshops und der anschließenden Gruppensupevision stehen gemeinsame Rollenspiele mit Perspektivwechsel. In den Rollenspielen spüren die Therapeuten jeweils einer erlebten Spannung oder Krise in der Therapiebeziehung nach, indem sie wechselseitig in die Patienten- oder Therapeutenrolle gehen. Dadurch bekommen sie die Möglichkeit, Einsicht in eigene Beiträge zur Interaktion zu gewinnen und (non-)verbale Handlungsweisen und unbewusste Beteiligungen („blinde Flecke“) wahrzunehmen. Um Zugang zu letzteren zu erhalten, wird das Doppeln (Psychodrama-Technik) eingesetzt. Ausgehend hiervon werden neue Möglichkeiten der Haltung und des Intervenierens erprobt, um die Spannungen und Krisen aufzulösen, indem das Wahrgenommene unter Nutzung von Anregungen und Impulsen der Supervisoren in Worte gefasst wird (Metakommunikation, Gumz 2023 a,b; Gumz 2020a). Vor den Rollenspielen werden zudem einführende Achtsamkeitsübung angeboten, um den Zugang zu den eigenen Affekten zusätzlich zu unterstützen.

- Gumz A. (2024). Messen und Trainieren therapeutischer Kompetenzen. Psychother Psychosom Med Psychol. 2024;74,12, 491-497.
- Gumz A (2023). Trainieren und Messen therapeutischer Beziehungskompetenzen. *PDP*, 22, 204-216.
- Gumz A (2020). *Kompetent mit Spannungen und Krisen in der therapeutischen Beziehung umgehen. Techniken und didaktische Konzepte.* Göttingen: Vandenhoeck & Ruprecht.
- Gumz A, Reuter L, Flückiger C, et al. (2020). Umgang mit Spannungen und Krisen in der therapeutischen Beziehung: Erste Erfahrungen mit einem handlungsorientierten Ausbildungs- und Supervisionskonzept. *Psychother Psych Med, 70*, 122-129.
- Gumz A, Rugenstein K, Munder T (2018). Allianz-Fokussiertes Training (AFT). Schulenübergreifender Weg zum Umgang mit Krisen in der therapeutischen Beziehung. *Psychotherapeut, 63*, 55–61.
- Gumz, A. (2019). Mit Spannungen und Krisen in der Therapiebeziehung kompetent(er) umgehen. PiD,20, 39-44.
- Safran, J.D. & Muran J.C. (2000). Negotiating the therapeutic alliance. A relational treatment guide. NY: Guilford.

**b) Wichtige Regelungen zur Gewährleistung der Studienqualität:**

In der Studie könnten methodische Verzerrungen entstehen, wenn Teilnehmende der Kontrollgruppe (Therapeuten, Supervisoren, Patienten) Informationen über Inhalte der Intervention (MAFT-D) erhalten. Das betrifft zum einen schriftliches Material und zum anderen die Kommunikation über die Intervention. Wichtig ist daher die Beachtung folgender Regelungen:

1. Zur objektiven Gewinnung von Studiendaten ist es notwendig, dass die Ihnen zugeordneten Studienpatienten keinesfalls erfahren, in welcher Studienbedingung Sie sich befinden („Verblindung“). Die Therapeuten werden über die Zuteilung informiert, die Patienten werden verblindet. Die Therapeuten (und die Supervisoren, sollten diese Kontakt zu den Patienten haben) dürfen über ihre Zuteilung oder über MAFT-D **nicht mit ihren Patienten** sprechen. Falls sich Patienten im Studienverlauf bzgl. Ihrer Zuordnung oder der Intervention erkundigen, bitten wir, auf Basis der folgenden standardisierten Antwort darauf zu reagieren:

„*Die Zuordnung darf ich leider nicht verraten, da Ihre Antworten in den Fragebögen und Interviews nicht davon beeinflusst werden sollen. Ihre Antworten sind sehr wertvoll, egal in welcher Gruppe Sie sind. Das liegt daran, dass mit der Studie nicht nur das Training für die Therapeuten (und die Supervisoren) geprüft werden soll, sondern es wird auch untersucht, welche Faktoren zum Erfolg einer Therapie allgemein beitragen. Solches Wissen ist sehr wichtig für die Verbesserung der Ausbildung von Therapeuten und für die Verbesserung von Therapien. Wie Sie wissen beschägtigt sich das Training mit der therapeutischen Beziehung. Das ist ein Thema, das für alle Therapeuten wichtig ist*.“

1. Alle Therapeuten und Supervisoren der Interventionsgruppe verpflichten sich **generell**, den Inhalt der Intervention (Workshop, Material, Vorgehen, Supervision) bis zum Abschluss der Studie **streng vertraulich** zu behandeln und Verschwiegenheit darüber zu bewahren. Dies betrifft die Weiterverbreitung innerhalb von Fort- oder Weiterbildungen, die über die Studienlaufzeit hinweg untersagt ist. Darüber hinaus dürfen Sie, d.h. die Therapeuten der Interventionsgruppe über die Studienlaufzeit hinweg **keinesfalls** **mit** **Therapeuten oder Supervisoren der Kontrollgruppe und auch nicht mit anderen Ausbildungskollegen oder Supervisoren an Ihrem Institut** über Inhalte der Intervention (des Trainings, des Vorgehens in der Supervision etc.) sprechen. (Auch die Supervisoren der Interventionsgruppe verpflichten sich auf diese genannten Vorgaben.) Wenn Sie nach der Intervention gefragt werden sollten, dürfen Sie Ihren allgemeinen persönlichen Eindruck dazu teilen (z.B. „*hilfreich, ganz interessant, passt gut oder nicht so gut zu meinem Stil*“ etc.). Darüber hinaus bitten wir Sie, eine Mitverantwortung für die Einhaltung der Regelungen zur Verschwiegenheit zu übernehmen, da die Qualität der Studie entscheidend davon abhängt.

Hinweis: Patienten, die den Therapeuten bzw. die Therapeutin wechseln, bleiben in der gleichen Studienbedingung.

**Prüfstelle:** Köln-Bonner Akademie für Verhaltenstherapie (KBAV), Wenzelgasse 35

53111 Bonn, Dr. phil. Lisa Miebach, **************

**Zentrales Studienteam:** Professur für Psychosomatik und Psychotherapie, Psychologische Hochschule Berlin (PHB), Am Köllnischen Park 2, 10179 Berlin, [a.gumz@phb.de](mailto:a.gumz@phb.de)

**Prüfer:** Prof. Dr. Antje Gumz

**Sponsor der klinischen Studie:** Psychologische Hochschule Berlin (PHB), Am Köllnischen Park 2, 10179 Berlin

EU trial number: DRKS00014842

**Einwilligungserklärung – zweiter Aufklärungsschritt**

**Zur Teilnahme an der wissenschaftlichen Untersuchung ,,Randomisiert kontrollierte Multicenter-Studie zur Therapieausbildung“.**Projektnummer 504346851

Ich bin in einem persönlichen Gespräch durch ein Mitglied des Studienteams der Psychologischen Hochschule Berlin

.... Prof. Dr. Antje Gumz ....................................................................

Name der aufklärenden Person

ausführlich und verständlich über den neuen Trainings- und Supervisionsansatz, der im Rahmen der klinischen Studie getestet wird, aufgeklärt worden. Ich habe den zugehörigen Text der Probandeninformation gelesen und verstanden. Ich hatte die Gelegenheit, ausreichend über die Durchführung der klinischen Studie zu sprechen. Alle meine Fragen wurden zufrieden stellend beantwortet.

Ich hatte ausreichend Zeit, mich zu entscheiden.

Mir ist bekannt, dass ich jederzeit und ohne Angabe von Gründen meine Einwilligung zur Teilnahme an der Studie zurückziehen kann (mündlich oder schriftlich), ohne dass mir daraus Nachteile entstehen.

**Ich willige ergänzend zu meiner im ersten Aufklärungsschritt gegebenen Einwilligung freiwillig ein, an der klinischen Studie als Mitglied der Interventionsgruppe wie oben beschrieben teilzunehmen.**

**Ich verpflichte mich, den Inhalt der Intervention (Workshop, Material, Vorgehen, Supervision) bis zum Abschluss der Studie streng vertraulich zu behandeln und Stillschweigen darüber zu bewahren.**

Ein Exemplar der ergänzenden Studieninformation und -einwilligung habe ich erhalten. Ein Exemplar verbleibt in der Prüfstelle.

...........................................................................................................................

Name des **Therapeuten bzw. der Therapeutin** in Druckbuchstaben

........................................

geb. am

.................................... ..............................................................................................

Ort/Datum Unterschrift des **Therapeuten bzw. der Therapeutin**
