# Supplementary material for: Modified Alliance-Focused Training with Doubling as an integrative approach to improve therapists’ competencies in dealing with alliance ruptures and prevent negative outcomes in psychotherapy for depression: study protocol of a randomised controlled multicentre trial
Source: BMJ Open. 2025 Jul 16;15(7):e098343. doi: 10.1136/bmjopen-2024-098343 (PMC12273124; doi:10.1136/bmjopen-2024-098343)
Supplement: Supplementary file 5 [file bmjopen-2024-098343.reviewer_comments.pdf]

PEER REVIEW HISTORY

BMJ Open publishes all reviews undertaken for accepted manuscripts. Reviewers are asked to complete a checklist review form and are provided with free text boxes to elaborate on their assessment. These free text comments are reproduced below.

ARTICLE DETAILS

Title (Provisional)

Modified Alliance-Focused Training with Doubling as an integrative approach to improve therapists' competencies in dealing with alliance ruptures and prevent negative outcome in psychotherapy for depression. Study protocol of a randomized controlled multicenter trial

Authors

Gumz, Antje; Kästner, Denise; Reuter, Laurence; Martinez Moura, Carmen; Ehlers, Klarissa; Daubmann, Anne; Eubanks, Catherine F.; Muran, J. Christopher; Anderson, Timothy; Stöckl, Ramona; Schwanitz, Georg; Stegemann, Lena; Rohr, Lana; Willutzki, Ulrike; Jacobi, Frank; Zapf, Antonia

---

VERSION 1 - REVIEW

**Reviewer**	**1**
**Name**	**Trkulja, Vladimir**
**Affiliation**	**University of Zagreb School of Medicine, Pharmacology**
**Date**	**12-Apr-2025**
**COI**	**None**

It was a pleasure to review the manuscript bmjopen-2024-098343. It is a protocol for a very complex randomized trial aimed at evalution of the effects of a specific therapists' (and supervisors') training focused on improving the skills of recognition and correction of potential alliance ruptures that may occur during psychotherapy (CBT or PDT) in patients suffering major depressive disorder(s). Hence the "test" treatment is receiving such a training vs. a control of "training as usual", which is then combined (randomized) with CBT or PDT. There is an extensive list of instruments and measurements planned to be implemented over 3 study years (primary endpoints at 20 weeks, follow-ups at 35 weeks, 20 and 36 months), with the purpose of assessing various factes of (presumed) benefits for the patients, but also for the characteristics/performance/skills of the therapists (and supervisors). I hope that the investigators will indeed be able to conduct it in line with the plan, that is, at some reasonably high level of compliance with the numerous study procedures. The protocol is clearly presented. Since I have no expertise in psychotherapy, my

comments (listed in no specific order) may reflect misunderstandings that might occur in non-expert readers.

1. In the section “Randomization and stratification”, p. 8, the sentence “Patients are assigned to therapists appropriate to the random group” appears a bit vague. In the first randomization step, therapists are randomized to Treatment (MAFT-D) or Control (TAU). Based on the list of 16 centers and two therapeutic methods (CBT and PDT), and the declared stratification process, there will be 32 strata for randomization of the therapists – is this correct ? In the second randomization step, patients are randomized to MAFT-D or TAU. Based on the declared stratification process, there will be 16 centers x 2 therapeutic modalities (CBP/PDT) x 2 comorbid personality disorders (yes/no), hence there will be 64 strata for randomization of the patients – is this a correct understanding? In this context, what does the sentence “Patients are assigned to therapists appropriate to the random group” mean? From the other parts of the text, it may be understood that it is planned/expected to have (preferably) 8 therapists per center, 4 of whom would be MAFT-D trained, and 4 would be TAU trained. Then, 2/4 MAFT-D would practice CBT and 2/4 would practice PDT – and the same for TAU-trained. Let’s assume a patient with a personality disorder who is to be treated by a MAFT-D-trained-CBT therapist – how is it decided to which one of the 2 MAFT-D-trained-CBT practicing therapist within a particular center is the patient assigned? Namely, from what is written, it may be understood that a stratum defined as, for example, “center No 1 – MAFT-D – CBT – personality disorder” would actually have “two options” regarding the therapist. Could it happen , for example, that one MAFT-D-trained-CBT practicing therapist within a particular center treats two patients with a personality disorder, whereas the other one treats two patients without a personality disorder? If such a case is possible, couldn’t there be “aliasing” between “therapist” and “personality disorder”? Does this matter?

2. As in any trial, it seems possible that in some centers the recruitment would go faster than in some other centers. The trial outline rather specifically depicts “2 patients per therapist” (on average) (Figure 1) as an expectation, or even more – as one of the basic elements of the trial. It might be feasible to explicitly state how this “balance” across centers/therapists is to be achived – for example, recruitment in a particular center/therapist will be stopped once the target number of patients per center-therapist is achieved. Could such a declaration contribute to clarity of the trial presentation?

3. Assume that 50% of the patients have a personality disorder. As planned, this would mean that 120/240 have a personality disorder, of those 60 are assigned to MAFT-D and 60 to TAU. Within each treatment group, 30 would undergo CBT and 30 would undergo PDT. The stratified randomization plan intends to “distribute” them comparably across the 16 centers, but obviously, considering the absolute numbers, there will be some minor differences – if recruitment of such patients is comparable across centers. Do authors intend to achieve “similarity” in this respect across centers? This would require – just as in the case of the number of patients per center – that some contraints are imposed.

4. The analytical models for the primary endpoints envisage a rather complex random effects structure: patients nested in therapist as a cluster, and then also “center” or even “supervision group” (cluster nested in the supervision group, and cluster-supervision group within center..or?). But it appears to me that this might be by far too complex and not really needed, and that the models might not converge. For example, all included centers are in Germany, all function within the same general conceptual-historical-educational frame, all therapists and supervisors will undergo a standardized training program. Why would one expect that there would be “correlation within centers”? Hence, there would be “zero variance” across centers, the model would likely not converge, and the variable would need to be removed.. Or, for that matter, “within supperivison groups”? In reality, what is reasonable to expect is variability across therapists, i.e., correlation between patients “within” therapists. If one would like to account for “center” as a stratification variable, it might be included in the model as a fixed effect. The model is more likely to converge, with 15-16 patients per center (as expected) the analysis is meaningful, accounting for stratification factor improves precision. On the other hand, stratification at the patient level for center, modality (CBT/PDT), and personality disorder – i.e., a total of 64 strata for 240 patients in total (i.e, less then 4 per stratum) – might actually be an overstratification. It seems to me that unless constraints are imposed to the number of patients per center, per modality (CBT/PDT), per personality disorder – the expected benefits of stratified randomization are likely to be lost.

5. The two primary outcomes (first – change in BDI vs. baseline; second – drop-out rate) are defined as a single time-point outcomes: (i) BDI change at week 20 vs. baseline; (ii) drop-out up to week 20 (yes-no). The proposed analytical model includes “time” (i.e., “visit”) and treatment*time interaction. But it seems to me that this is not needed for the primary outcomes. I think that a simpler model for the 1st primary outcome could accurately estimate the “main treatment effect” (general linear mixed model; outcome: BDI change; fixed effects: treatment (MAFT-D vs. TAU), modality (CBT vs. PDT), personality disorder, center, baseline bdi; random: patient nested in therapist). In the second and third runs, the model might evaluate homogeneity of effects across treatment modality (CBT/PDT as a moderator?), and across levels of personality disorder, respectively, by including treatment*modality, and treatmet*personality interaction terms, respectively, or, in a fourth run, treatment*modality*personality interaction to generate treatment contrasts across the levels of modality-by-personality disorder. A similar approach would be applicable for the 2nd primary outcome..

6. For follow-up outcomes – 35 weeks, 20 months etc – models with repeated measures could be re-fitted.

7. The fact that BDI is to be evaluated before the 5th session and then considered as a potential mediator of the primary outcomes, in my opinon, has nothing to due with the “primary analysis” of the primary outcomes. (ie. no need for repeated-measures models).

8. I understand that persistence of an effect in treatmet of psychiatric disorders is an important element. However, in this particular trial – as outlined – without a defined specific methodology to deal with intercurrent events (possibly introducing post-randomization bias), or to account for both post-randomization bias and selection bias, that are highly likely to occur over a 20- or 36-month period, assessment of "effect persistence" or assessment of "long-term effects" is questionable (e.g., Hernan MA et al. Ann Intern Med 2013; 159:560-562).

9. Duration of the patient treatment (CBT or PDT) is not explicitly stated. The protocol envisages that it may vary – depending on the severity of the condition, as indicated by the therapist, as covered by the insurance etc. I understand that this is a "real life" fact. I have no idea how psychotherapy functions, i.e., what is the usual "dose", e.g,. 8 weekly sessions? 12, 16..?. In particular, I have no idea how does this function in Germany. On the other hand, the following assumptions seem reasonable to me: a more severly affected patient and/or combination with personality disorder, might require longer treatment. Adjusting for baseline severity and personality disorder is likely to "remove" their confounding effects, but a patient that is scheduled for a longer treatment (i) is longer at risk of dropping out; (ii) might be more likely to benefit (receives a "higher dose"); (iii) might be more likely to have a greater reduction at 20 weeks, since time elapsed since the stop of treatment to 20 weeks is shorter than for a patient who was scheduled for and received less weekly sessions. Overall, it seems reasonable to view discrepancy in the scheduled treatment duration as a potential source of bias. The manuscript would benefit from a text/explanation dealing with this topic.

10. Part on analysis, p. 27, lists a number of presumed mediators. To me, some parts of this section are somewhat unclear. For example, one of the listed presumed mediators is "patient-rated therapeutic alliance across sessions". In my understanding, however, this is likely a collider: (a) Intervention might affect it; (b) outcome (reduction in depressive symptoms) (induced by the intervention through some other path) might affect it. Hence, it is a variable on a path between X and Y and is affected by both X and Y, (or is a descendant of a variable caused by both X and Y) hence, by definition – it is a collider. The causal mediation analysis includes (1) assessing the effect of both the cause and the presumed mediator on the outcome, (2) assessing the effect of the mediator on the outcome. In step (1), one estimates X on Y while conditioning on the presumed mediator. And if a collider – then this will cause (endogenous) selection bias: an association between X and Y will be detected, which is not causal. Ratio of unresolved/resolved alliance ruptures is also one of the presumed mediators (patient-rated). But (a) it could also be a collider; (b) in many patients, this value might be 0 (0 ruptures, or 0 resolved). May not be feasible for modelling. In the discussion on the trial design, its reaches and limitations, the authors emphasize that this is the first RCT to evaluate the effect of this particular intervation on the patient outcomes, as opposed to previous ones that considered "therapists' skills" as outcomes. It seems to me that "therapists skills" (operationalized by variables unlikely to be affected by the outcome) are "natural choices" for mediators.

11. Part of analysis, p.27 – “additional analyses”. Indicates a test of treatment modality as a moderator. It seems to me that the choice of covariates in the model should be based on the general knowledge about potential impact of a particular baseline property on the outcome, like disease severity, comorbid personality disorder, and possibly age or similar. Statistical significance of differences in baseline variables between CBT and PDT patients might not be the adequate criterion for this purpose.

12. Based on inclusion-exclusion criteria, patients to be enrolled will be allowed to have pharmacotherapy in place (limited to specifically depression-targeted pharmacotherapy). To my understanding, there is some evidence that, e.g., CBT+pharmacotherapy provide higher probability of symptom improvement vs. individual components, at least over some shorter treatment period. The manuscript/protocol should to some extent address this potential issue: namely, (existing) pharmacotherapy is not envisaged as a covariate in the data analysis models, and there is no mention about expected numbers of patients using pharmacotherapy at the trial enrollment.

13. To my understanding, the patients are to be recruited from a pool of patients eligible in the included specialized institutions. Is it possible that they tell a difference between CBT and PDT (e.g., based on previous experience), and could this affect the outcomes?

14. The authors have put some accent on the external validity of this trial, where two arguments are apparently perceived as crucial: i) the trial is multicentric, involving 16 centers/specialized institutions in Germany; ii) inclusion-exclusion criteria (for patients) are relatively simple [DSM-V-based diagnosis of a depressive disorder, a certain minimum score on BDI II (at least moderate depression), a lack of suicidal ideation, and a lack of a recent changes (in potential) pre-existing pharmacotherapy], which, presumably should result in “inclusivity” (or “pragmatic nature” of the trial) and representativeness of the sample (for the target population). As mentioned, I have no idea how the mental health care functions in Germany. Some information that could be obtained from the published material (e.g., Engels A et al. Depression treatment in Germany…… BMC Psychiatry 2020; 20:591), suggests that patients to be included in this trial might not be “ideally representative” of a target population defined as “patients with MDD, with of at least moderately severe disease, and no suicidal ideation “ : i) at least the “1st episode-moderate severity depression” patients, to my understanding, are also treated in primary care; ii) patients need to agree to the option of psychotherapy; iii) how important for generalizability of this trial results are cross-cultural perception of MDD, applicability of CBT/PDT, and of the evaluated intervention? Overall, I agree that this trial – should it be executed as planned – is likely to achive a high level of internal validity. I also agree that (in such a case), the trial results would be important practically. It is only that some limitations to generalizability are likely. This, of course, is only a minor point (particularly if viewed vs. all the trial values).

I wish the investigators all the best in the trial execution.

VERSION 1 - AUTHOR RESPONSE

Reviewer: 1
Prof. Vladimir Trkulja, University of Zagreb School of Medicine
Comments to the Author:

It was a pleasure to review the manuscript bmjopen-2024-098343. It is a protocol for a very complex randomized trial aimed at evalution of the effects of a specific therapists' (and supervisors') training focused on improving the skills of recognition and correction of potential alliance ruptures that may occur during psychotherapy (CBT or PDT) in patients suffering major depressive disorder(s). Hence the "test" treatment is receiving such a training vs. a control of "training as usual", which is then combined (randomized) with CBT or PDT. There is an extensive list of instruments and measurements planned to be implemented over 3 study years (primary endpoints at 20 weeks, follow-ups at 35 weeks, 20 and 36 months), with the purpose of assessing various factes of (presumed) benefits for the patients, but also for the characteristics/performance/skills of the therapists (and supervisors). I hope that the investigators will indeed be able to conduct it in line with the plan, that is, at some reasonably high level of compliance with the numerous study procedures. The protocol is clearly presented. Since I have no expertise in psychotherapy, my comments (listed in no specific order) may reflect misunderstandings that might occur in non-expert readers.

Answer: We would like to emphatically thank you for the time and effort that must have gone into reviewing our manuscript. We are convinced that the many helpful comments have enabled us to significantly improve the quality of our work.

1. In the section "Randomization and stratification", p. 8, the sentence "Patients are assigned to therapists appropriate to the random group" appears a bit vague. In the first randomization step, therapists are randomized to Treatment (MAFT-D) or Control (TAU). Based on the list of 16 centers and two therapeutic methods (CBT and PDT), and the declared stratification process, there will be 32 strata for randomization of the therapists – is this correct ? In the second randomization step, patients are randomized to MAFT-D or TAU. Based on the declared stratification process, there will be 16 centers x 2 therapeutic modalities (CBP/PDT) x 2 comorbid personality disorders (yes/no), hence there will be 64 strata for randomization of the patients – is this a correct understanding? In this context, what does the sentence "Patients are assigned to therapists appropriate to the random group" mean? From the other parts of the text, it may be understood that it is planned/expected to have (preferably) 8 therapists per center, 4 of whom would be MAFT-D trained, and 4 would be TAU trained. Then, 2/4 MAFT-D would practice CBT and 2/4 would practice PDT – and the same for TAU-trained. Let's assume a patient with a personality disorder who is to be treated by a MAFT-D-trained-CBT therapist – how is it decided to which one of the 2 MAFT-D-trained-CBT practicing therapist within a particular center is the patient assigned? Namely, from what is written, it may be understood that a stratum defined as, for example, "center No 1 – MAFT-D – CBT – personality disorder" would actually have "two options" regarding the therapist. Could it happen , for example, that one MAFT-D-trained-CBT practicing therapist within a particular center treats two patients with a personality disorder, whereas the other one treats two patients without a personality disorder? If such a case is possible, couldn't there be "aliasing" between "therapist" and "personality disorder"? Does this matter?

Answer: First, therapists are randomized to MAFT-D or TAU, stratified by center and theoretically stratified by the two therapeutic approaches (CBT and PDT). In practice, however, all of the centers offer only one therapeutic approach. We now state this clearly under "Methods against bias" (p. 9) and updated the list of centers provided in the manuscript including the respective therapeutic approach (see p. 14-15). Accordingly, there are a total of 17 strata for the randomization of therapists.

Patients are randomized stratified by center (what is in this case identical with therapeutic approach) and comordbid personality disorder (yes/no). For 17 centers, it results in 34 strata.
The allocation to a specific therapist within the assigned study group is based on several practical factors: scheduling compatibility (for the regular weekly session), patient and therapist preferences (e.g., gender, treatment location), and the therapist's current study case load. The distribution of patients with and without comorbid personality disorders is not controlled at the individual therapist level, due to feasibility constraints and expected variation in prevalence across centers. As a result, imbalances such as one therapist treating more patients with personality disorders than another may occur. While we agree that a perfectly equal distribution across therapists would be ideal, it is not practically achievable under real-world conditions. However, we will statistically control for the presence of personality disorder diagnoses in all relevant analyses. These allocation procedures have now been described more explicitly in the revised manuscript (see p. 16-17).

2. As in any trial, it seems possible that in some centers the recruitment would go faster than in some other centers. The trial outline rather specifically depicts "2 patients per therapist" (on average) (Figure 1) as an expectation, or even more – as one of the basic elements of the trial. It might be feasible to explicitly state how this "balance" across centers/therapists is to be achived – for example, recruitment in a particular center/therapist will be stopped once the target number of patients per center-therapist is achieved. Could such a declaration contribute to clarity of the trial presentation?

Answer: In general, recruitment at a given center will be concluded once the target number of patients per center has been reached (i.e., typically two patients per therapist as outlined in Figure 1). This limit is directly linked to the predefined supervision structure and duration agreed upon with the participating supervisors and therapists. We do not expect slower patient recruitment at individual centers to pose major problems for the trial, because recruitment takes place in waves, centers are not initiated at the same time, and the overall timeline allows for certain delays. Nevertheless, we allow for minor deviations in the number of study patients treated by each therapist (i.e., one or three instead of two), e.g. due to therapist availability and the number of open treatment and supervision slots.

3. Assume that 50% of the patients have a personality disorder. As planned, this would mean that 120/240 have a personality disorder, of those 60 are assigned to MAFT-D and 60 to TAU. Within each treatment group, 30 would undergo CBT and 30 would undergo PDT. The stratified randomization plan intends to "distribute" them comparably across the 16 centers, but obviously, considering the absolute numbers, there will be some minor differences – if recruitment of such patients is comparable across centers. Do authors intend to achieve "similarity" in this respect across centers? This would require – just as in the case of the number of patients per center – that some contraints are imposed.

Answer: Achieving a perfect similarity regarding personality disorders across centers is not intended, as this does not appear feasible under real-world conditions. We expect the prevalence of personality disorders to vary between centers. Additionally, the inclusion and exclusion criteria, along with the screening procedures, are already relatively complex, and recruitment is time-sensitive, as each therapist requires at least one assigned study patient in order to initiate group supervision.
Therefore, stratification for personality disorder within centers is only intended to ensure that MAFT-D and TAU are balanced between these two groups. Minor imbalances due to randomization between the groups are acceptable.

4. The analytical models for the primary endpoints envisage a rather complex random effects structure: patients nested in therapist as a cluster, and then also "center" or even "supervision group" (cluster nested in the supervision group, and cluster-supervision group within center..or?). But it appears to me that this might be by far too complex and not really needed, and that the models might

not converge. For example, all included centers are in Germany, all function within the same general conceptual-historical-educational frame, all therapists and supervisors will undergo a standardized training program. Why would one expect that there would be “correlation within centers”? Hence, there would be “zero variance” across centers, the model would likely not converge, and the variable would need to be removed. Or, for that matter, “within supperivison groups”? In reality, what is reasonable to expect is variability across therapists, i.e., correlation between patients “within” therapists. If one would like to account for “center” as a stratification variable, it might be included in the model as a fixed effect. The model is more likely to converge, with 15-16 patients per center (as expected) the analysis is meaningful, accounting for stratification factor improves precision. On the other hand, stratification at the patient level for center, modality (CBT/PDT), and personality disorder – i.e., a total of 64 strata for 240 patients in total (i.e, less then 4 per stratum) – might actually be an overstratification. It seems to me that unless constraints are imposed to the number of patients per center, per modality (CBT/PDT), per personality disorder – the expected benefits of stratified randomization are likely to be lost.

Answer: Thank you for your comments. Your concerns about convergence problems due to the complex cluster structure are justified. Even if all centers are located in Germany, they can still differ, for example due to their size and different catchment areas (urban vs. rural, etc.), which is why we decided to consider them as a random effect. In this way, we represent the structure of the data generating process with the model structure described. Furthermore, existing research suggests an overestimation of the therapist effect If the institution is not considered (e.g., Deisenhofer, Hehlmann, et al., 2024). If the models do not converge due to the cluster structure and estimate some variance components to zero, we will remove the variance component with a zero estimation from the model without losing information (see p. 31).

5. The two primary outcomes (first – change in BDI vs. baseline; second – drop-out rate) are defined as a single time-point outcomes: (i) BDI change at week 20 vs. baseline; (ii) drop-out up to week 20 (yes-no). The proposed analytical model includes “time” (i.e., “visit”) and treatment*time interaction. But it seems to me that this is not needed for the primary outcomes. I think that a simpler model for the 1st primary outcome could accurately estimate the “main treatment effect” (general linear mixed model; outcome: BDI change; fixed effects: treatment (MAFT-D vs. TAU), modality (CBT vs. PDT), personality disorder, center, baseline bdi; random: patient nested in therapist). In the second and third runs, the model might evaluate homogeneity of effects across treatment modality (CBT/PDT as a moderator?), and across levels of personality disorder, respectively, by including treatment*modality, and treatmet*personality interaction terms, respectively, or, in a fourth run, treatment*modality*personality interaction to generate treatment contrasts across the levels of modality-by-personality disorder. A similar approach would be applicable for the 2nd primary outcome.

Answer: Thanks for your suggestions.
We decided to fit one mixed model including all time points (up to the first follow-up), because it allows for implicit imputation of missing values which results in unbiased estimates under the MAR assumption (CHMP Guideline on Missing Data in Confirmatory Clinical Trials (EMA/CPMP/EWP/1776/99 Rev. 1)). This assumption is verified through sensitivity analysis. We clarified that we will conduct the analysis for the first primary outcome (BDI-II) up to and including the first follow-up and will repeat them later in order to include all measurement points (i.e., follow-up 2+3, after 3 years).

The investigation of the treatment effect on treatment modality is planned and described in the section “Additional analyses”. A further and substantially more complex analysis of subgroups such as personality disorder is not planned.

6. For follow-up outcomes – 35 weeks, 20 months etc – models with repeated measures could be re-fitted.

Answer: We chose the mixed model approach instead of the repeated measures analysis of variance because it is easier to deal with missing data. One data point is sufficient for the patient to be part of the analysis population. Furthermore, it is possible to perform post-hoc tests for the repeated factor. There are more possibilities to model the dependency between the time points. It is also easier to extend the model to a more complex model.

7. The fact that BDI is to be evaluated before the 5th session and then considered as a potential mediator of the primary outcomes, in my opinon, has nothing to due with the "primary analysis" of the primary outcomes. (ie. no need for repeated-measures models).

Answer: There may be a misunderstanding here. We do not use the baseline value of BDI as a mediator. We fit a baseline adjusted model to obtain an estimate unaffected by possible baseline differences. So, we have greater statistical power to detect a treatment effect in comparison to omitting the baseline adjustment.

8. I understand that persistence of an effect in treatmet of psychiatric disorders is an important element. However, in this particular trial – as outlined – without a defined specific methodology to deal with intercurrent events (possibly introducing post-randomization bias), or to account for both post-randomization bias and selection bias, that are highly likely to occur over a 20- or 36-month period, assessment of "effect persistence" or assessment of "long-term effects" is questionable (e.g., Hernan MA et al. Ann Intern Med 2013; 159:560-562).

Answer: Thank you for expressing your concern, we agree that intercurrent events could affect long-term treatment effects of psychological interventions. Due to randomization, we assume that intercurrent events occur equally frequent in both groups, MAFT-D and TAU. To monitor and analyze pre-defined major events (e.g., hospitalizations), we implemented several methods. As outlined in Table 1, therapists can report significant events (e.g., onset of a serious illness) as they become aware of them, patients report on the use of additional treatments (e.g., newly prescribed psychotropic medications) after every session, and we assess healthcare utilization in more detail at the fixed measurement points (baseline, post-treatment, FUP1, FUP2, FUP3) using the adapted CSSRI. We have now provided further clarification in the manuscript regarding which additional treatments will be considered protocol violations (see p. 12) and the methods we will use to address such violations (see p. 32).

9. Duration of the patient treatment (CBT or PDT) is not explicitly stated. The protocol envisages that it may vary – depending on the severity of the condition, as indicated by the therapist, as covered by the insurance etc. I understand that this is a "real life" fact. I have no idea how psychotherapy functions, i.e., what is the usual "dose", e.g,. 8 weekly sessions? 12, 16..?. In particular, I have no idea how does this function in Germany. On the other hand, the following assumptions seem reasonable to me: a more severly affected patient and/or combination with personality disorder, might require longer treatment. Adjusting for baseline severity and personality disorder is likely to "remove" their confounding effects, but a patient that is scheduled for a longer treatment (i) is longer at risk of dropping out; (ii) might be more likely to benefit (receives a "higher dose"); (iii) might be more likely to have a greater reduction at 20 weeks, since time elapsed since the stop of treatment to 20 weeks is shorter than for a patient who was scheduled for and received less weekly sessions. Overall, it seems reasonable to view discrepancy in the scheduled treatment duration as a potential source of bias. The manuscript would benefit from a text/explanation dealing with this topic.

Answer: Thanks for your helpful comment, which prompted us to include crucial information regarding the number of therapy sessions covered by German payers (see p. 17). Accordingly, a regularly completed treatment consists of at least 14 sessions (2 probatory sessions plus 12 sessions of short-term therapy). However, in practice, most therapists utilize all available probatory sessions (i.e., 4 sessions), and it is uncommon for therapy to end after just the first unit of short-term therapy (i.e., 12 sessions). Therefore, we do not expect a higher treatment dose in more severely affected patients during the first 20 weeks.
We agree with the reviewer that adjusting for baseline severity and the presence of comorbid personality disorders should remove potential confounding effects. Furthermore, the aim of randomization is to ensure that patient characteristics are evenly distributed between the two groups (MAFT-D and TAU).

10. Part on analysis, p. 27, lists a number of presumed mediators. To me, some parts of this section are somewhat unclear. For example, one of the listed presumed mediators is "patient-rated therapeutic alliance across sessions". In my understanding, however, this is likely a collider: (a) Intervention might affect it; (b) outcome (reduction in depressive symptoms) (induced by the intervention through some other path) might affect it. Hence, it is a variable on a path between X and Y and is affected by both X and Y, (or is a descendant of a variable caused by both X and Y) hence, by definition – it is a collider. The causal mediation analysis includes (1) assessing the effect of both the cause and the presumed mediator on the outcome, (2) assessing the effect of the mediator on the outcome. In step (1), one estimates X on Y while conditioning on the presumed mediator. And if a collider – then this will cause (endogenous) selection bias: an association between X and Y will be detected, which is not causal. Ratio of unresolved/resolved alliance ruptures is also one of the presumed mediators (patient-rated). But (a) it could also be a collider; (b) in many patients, this value might be 0 (0 ruptures, or 0 resolved). May not be feasible for modelling. In the discussion on the trial design, its reaches and limitations, the authors emphasize that this is the first RCT to evaluate the effect of this particular intervation on the patient outcomes, as opposed to previous ones that considered "therapists' skills" as outcomes. It seems to me that "therapists skills" (operationalized by variables unlikely to be affected by the outcome) are "natural choices" for mediators.

Answer: Thank you for your insightful comment. We agree that patient-rated therapeutic alliance could be a collider, as it may be influenced by both the intervention and the outcome (symptom reduction). However, based on the current state of knowledge in psychotherapy research, we believe that patient-rated therapeutic alliance is a crucial mediator. While it may be influenced by both the intervention and the outcome, we hypothesize at least a medium effect on treatment outcomes. To address the potential collider bias, we will first test for its presence (e.g., using directed acyclic graphs), and, if detected, apply appropriate statistical methods, such as latent variable modeling. Regarding the ratio of unresolved/resolved alliance ruptures, we agree that it could also be a collider, and some patients may have no ruptures. However, we believe that the non-occurrence of ruptures could be just as important as the occurrence of ruptures. If too many patients have no ruptures, which might affect the variability of this variable, we will explore categorizing it (e.g., into three groups: mostly resolved ruptures, mostly unresolved ruptures, no ruptures, or simply with or without ruptures). We also agree with your comments on the relevance of therapist-related mediators. Mediators such as therapist competence in session (empathy), adherence to AFT techniques, and the use of specific interventions were already planned to be included in our analysis, as they may be key to better understanding treatment processes.

11. Part of analysis, p.27 – "additional analyses". Indicates a test of treatment modality as a moderator. It seems to me that the choice of covariates in the model should be based on the general knowledge about potential impact of a particular baseline property on the outcome, like disease

severity, comorbid personality disorder, and possibly age or similar. Statistical significance of differences in baseline variables between CBT and PDT patients might not be the adequate criterion for this purpose.

Answer: We agree with your comment and have added a clarification in the manuscript to address this (see p. 33). Covariates will be selected based on clinical and theoretical relevance, rather than solely on statistical significance.

12. Based on inclusion-exclusion criteria, patients to be enrolled will be allowed to have pharmacotherapy in place (limited to specifically depression-targeted pharmacotherapy). To my understanding, there is some evidence that, e.g., CBT+pharmacotherapy provide higher probability of symptom improvement vs. individual components, at least over some shorter treatment period. The manuscript/protocol should to some extent address this potential issue: namely, (existing) pharmacotherapy is not envisaged as a covariate in the data analysis models, and there is no mention about expected numbers of patients using pharmacotherapy at the trial enrollment.

Answer: Thank you for your comment. Since we assume that various potentially influencing variables, including pharmacotherapy, are equally distributed in the MAFT-D and TAU groups due to randomization, pharmacotherapy is not included as a covariate in the model.

13. To my understanding, the patients are to be recruited from a pool of patients eligible in the included specialized institutions. Is it possible that they tell a difference between CBT and PDT (e.g., based on previous experience), and could this affect the outcomes?

Answer: Since we stratify our randomization for CBT/PDT and include the variable into our model, it should not affect the intervention effect.

14. The authors have put some accent on the external validity of this trial, where two arguments are apparently perceived as crucial: i) the trial is multicentric, involving 16 centers/specialized institutions in Germany; ii) inclusion-exclusion criteria (for patients) are relatively simple [DSM-V-based diagnosis of a depressive disorder, a certain minimum score on BDI II (at least moderate depression), a lack of suicidal ideation, and a lack of a recent changes (in potential) pre-existing pharmacotherapy], which, presumably should result in "inclusivity" (or "pragmatic nature" of the trial) and representativeness of the sample (for the target population). As mentioned, I have no idea how the mental health care functions in Germany. Some information that could be obtained from the published material (e.g., Engels A et al. Depression treatment in Germany…… BMC Psychiatry 2020; 20:591), suggests that patients to be included in this trial might not be "ideally representative" of a target population defined as "patients with MDD, with of at least moderately severe disease, and no suicidal ideation " : i) at least the "1st episode-moderate severity depression" patients, to my understanding, are also treated in primary care; ii) patients need to agree to the option of psychotherapy; iii) how important for generalizability of this trial results are cross-cultural perception of MDD, applicability of CBT/PDT, and of the evaluated intervention? Overall, I agree that this trial – should it be executed as planned – is likely to achive a high level of internal validity. I also agree that (in such a case), the trial results would be important practically. It is only that some limitations to generalizability are likely. This, of course, is only a minor point (particularly if viewed vs. all the trial values).
I wish the investigators all the best in the trial execution.

Answer: Thank you very much for your thoughtful comment. We appreciate your recognition of the trial's internal validity and practical relevance. Regarding generalizability, we would like to clarify that guidelines (e.g., NICE) recommend psychotherapy as the first-line treatment for moderate depression, including first-episode patients. Additionally, patients with acute suicidality, who are excluded in line

with standard clinical practice, are typically not treated in outpatient settings. Overall, we aim to reflect a realistic treatment approach within clinical guidelines. We recognize that factors such as patient willingness to engage in psychotherapy, cross-cultural perceptions of MDD, and the applicability of CBT/PDT are important for generalizability. These aspects will be addressed in the discussion of the trial results.

VERSION 2 - REVIEW

**Reviewer**	**1**
**Name**	**Trkulja, Vladimir**
**Affiliation**	**University of Zagreb School of Medicine, Pharmacology**
**Date**	**20-Jun-2025**
**COI**	

I have no further comments.

And I hope that the trial will go as planned.
